# 1,8-Di(2-ethynylaryl)biphenylenes: Palladium-Catalyzed Intramolecular Cycloisomerization and Subsequent Thermal Rearrangement

**DOI:** 10.1021/acs.orglett.5c04302

**Published:** 2025-11-11

**Authors:** Hsiang-Han Chen, Chih-Hsuan Liu, Wei-Ting Ou, Kuan-Hsun Huang, Chia-Jung Yang, Mu-Jeng Cheng, Yao-Ting Wu

**Affiliations:** Department of Chemistry, 34912 National Cheng Kung University, 70101 Tainan, Taiwan

## Abstract

A cascade reaction for 1,8-dibromobiphenylene A with arylboronic acid B toward an unusual product C was comprehensively investigated. The initially formed Suzuki product D underwent an unprecedented palladium-catalyzed cycloisomerization, yielding E with a unique 5–8–5-membered ring framework. In most cases, E proceeded via thermal rearrangement to form C. This work also examined the scope and limitations of the cascade reaction, probed its mechanism, and verified the structures of C and E by X-ray crystallography.

## Graphical abstract

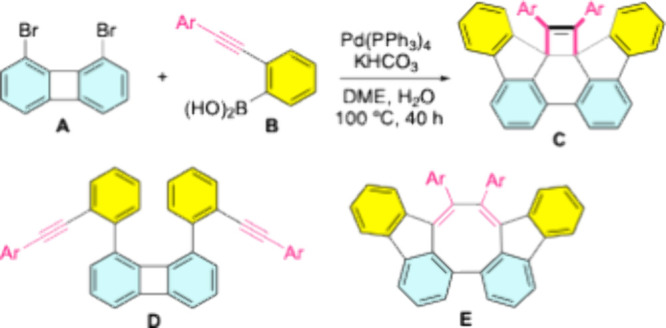

Phenylenes incorporated with four-membered rings are both σ- and π-activated arenes^1^ that serve as promising precursors for various polycyclic aromatic hydrocarbons (PAHs). A notable example is the metal-catalyzed annulation of biphenylene with alkynes, leading to the formation of phenanthrenes (Figure 1).^1a,1d^ (Over)­crowded PAHs, such as [7]­phenacene^2^ and bidibenzo­[*a*,*j*]­anthracene (BDBA),^3^ have been successfully prepared using this method. This synthetic toolbox has been further enriched by the metal-catalyzed intramolecular cycloisomerization of 1-(2-ethynylphenyl)­biphenylene, resulting in the formation of benz­[*e*]­acephenanthrylene (B*e*AP) and benzo­[*b*]­fluoranthene (B*b*F).^4^ Recently, we observed that 1,8-di­(2-ethynylphenyl)­biphenylene derivatives **3** undergo a novel palladium-catalyzed cycloisomerization (PCCI), forming cycloocta­[1,2,3-*jk*:8,7,6-*j′k′*]­difluorene **4** and its thermal rearrangement (TR) product, difluoreno-fused bicyclo[4.2.0]­octa-2,4,7-triene **2** (Scheme 1). This work investigates this new reaction mode for biphenylene and the unconventional TR from **4** to **2** (**4**/**2** TR) in most cases, which proceeds in the reverse direction compared to the typical transformation from bicyclo[4.2.0]­octa-2,4,7-triene (BCO) to cyclooctatetraene (COT, see below). Therefore, the structures and reactivity of **2** and **4** as well as their TR mechanism are explored herein.

**Figure 1:**
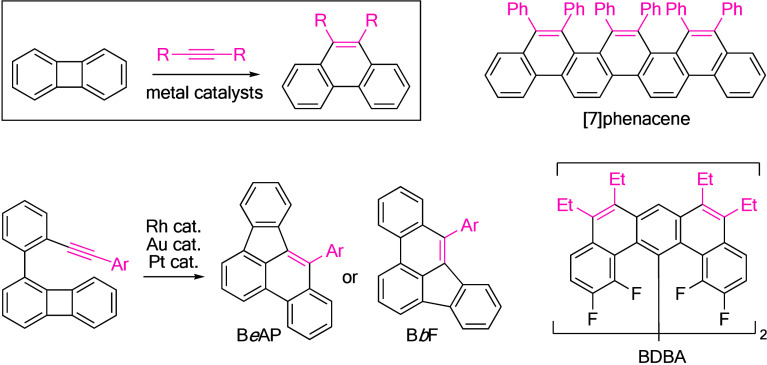
Metal-catalyzed annulations of [*n*]­phenylenes with alkynes.

**Scheme 1:**
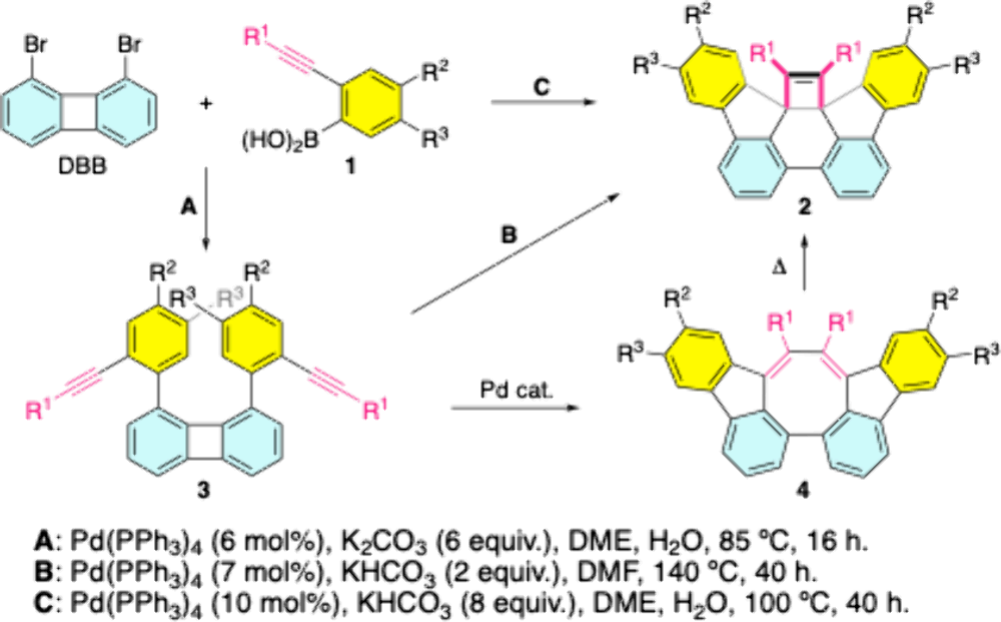
Palladium-Catalyzed Reactions of DBB with **1**

The Suzuki reaction of 1,8-dibromobiphenylene (DBB) with 2-(phenylethynyl)­phenylboronic acid (**1a**) under condition **A** (85 °C) yielded **3a** (79%) alongside a minor, unexpected product **4a** (8%, Scheme 1). Upon performing the reaction of **3a** under condition **A**, a mixture of **2a** and **3a** was obtained in a 77:23 ratio. In contrast, the exclusion of the palladium catalyst and the base led to complete suppression and deceleration of the PCCI, respectively. In the final case (absence of the base), a mixture of **2a**, **3a**, and **4a** in a 15:35:50 ratio was formed; nevertheless, **4a** was successfully isolated in pure form with a yield of 32% due to the purification challenges arising from the close overlap of the three compounds on the TLC plate. Under condition **B** (140 °C), **3a** was completely converted to **2a** with an 81% yield. Therefore, the PCCI step is promoted by base at elevated temperatures. Accordingly, these results support the reaction pathways proposed in Scheme 1 where **3a** undergoes PCCI to afford **4a**, which subsequently transforms into **2a** as a thermally rearranged product. Moreover, the **4a**/**2a** transformation in DMSO-*d*_6_, which was monitored over the 80–120 °C temperature range, revealed an estimated barrier (Δ*G* ^‡^) of 27.4 kcal/mol (Figure S1). Due to the thermal instability of **4a** and difficult separation from **3a** and **2a**, efforts were made to synthesize **2a** directly from DBB and **1a**. Although condition **B** effectively facilitated the **4a**/**2a** TR, it was unsuitable for the Suzuki reaction. Fortunately, condition **C** (100 °C) proved to be effective for producing **2a** with 71% and 56% yields on the 0.1 and 1.0 mmol scales, respectively (entry 1, Table 1). Despite reports of photoisomerization of substituted BCOs to COT derivatives,^5^ **2a** remained unaltered under UV irradiation at 254 and 365 nm.

**Table 1:** Reaction of DBB (0.1 mmol) with **1** under Condition **C**

Entry		R^1^	R^2^	R^3^	Product (yield, %)
1	**1a**	Ph	H	H	**2a** (71, 56^a^)
2	**1b**	4-^*n*^Bu-Ph	H	H	**2b** (76)
3	**1c**	4-OMe-Ph	H	H	**2c** (60)
4	**1d**	4-CF_3_–Ph	H	H	**2d** (64)^b^
5	**1e**	2-tolyl	H	H	—
6	**1f**	2-Cl-Ph	H	H	—
7	**1g**	2-naphthyl	H	H	**2g** (62)
8	**1h**	MN^c^	H	H	**2h** (67)
9	**1i**	Ph	H	*n*Bu	**2i** (66)
10	**1j**	Ph	OMe	H	**2j** (51)


a The reaction was conducted on a 1.0 mmol scale over a period of 50 h.

b The reaction was conducted for 60 h.

c MN = 7-methoxy-2-naphthyl.

The scope and limitations of this synthetic protocol were examined using a series of arylboronic acids **1** under condition **C** (Table 1). Arylboronic acids with 4-*n*-butylphenyl, 4-anisyl, 4-trifluoromethylphenyl, 2-naphthyl and 7-methoxy-2-naphthyl groups at the R^1^ position produced **2b** (76%), **2c** (60%), **2d** (64%), **2g** (62%), and **2h** (67%), respectively (entries 2, 3, 4, 7 and 8, respectively). Notably, the synthesis of **2d** required a long reaction time (60 h) because the electron-withdrawing substituent (CF_3_) slowed down the PCCI. Sterically bulky R^1^ groups, such as 2-tolyl and 2-chlorophenyl, did make the PCCI disfavored. The isolated products exhibited ^1^H NMR chemical shifts ranging from 5.0 to 6.0 ppm, which are resonances characteristic of **4a**, and were thus assigned to **4e** and **4f** [entries 5 and 6, respectively]. Nevertheless, both collected fractions contained inseparable, unidentified byproducts. In other words, pure samples of **4e** and **4f** could not be obtained.

A *n*-butyl group at the R^3^ position and a methoxy group at the R^2^ position slightly reduced the yields of the corresponding products **2i** (66%) and **2j** (51%) [cf. **2a**; entries 9 and 10, respectively]. Unexpectedly, treatment of DBB with [1-(phenylethynyl)­naphth-2-yl]­boronic acid (**1k**) under condition **C** primarily produced Suzuki product **3k** (65%, Scheme 2). Like 2-substituted phenyl groups at the R^1^ position, the steric congestion induced by the aryl moiety (highlighted in yellow) adjacent to the biphenylene core also exerted an unfavorable effect on PCCI. Under the conditions outlined in Scheme 2, **3k** afforded a mixture consisting of colorless **5** (8%), dark-blue **6** (25%), and various unidentified byproducts. Prolonging the reaction duration from 16 to 40 h slightly enhanced the yield of **6** (31%) but led to the disappearance of **5**, suggesting its comparatively lower stability. The disfavored formation and high reactivity of **4k** are most likely due to significant steric strain (see below). Compounds **5** and **6** are presumably derived from the reaction of **4k** with water molecules.

**Scheme 2:**
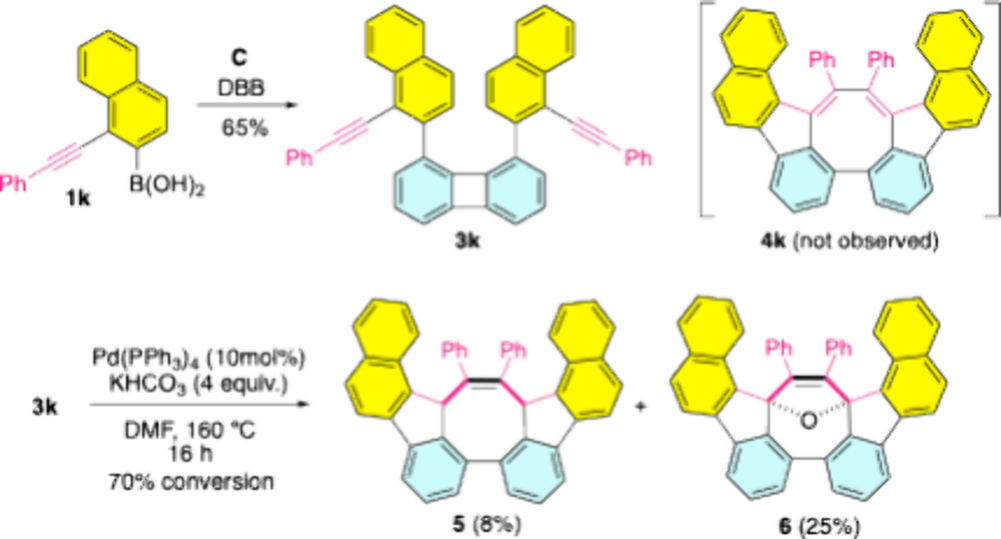
Synthesis of Compounds **5** and **6**

**Scheme 3:**
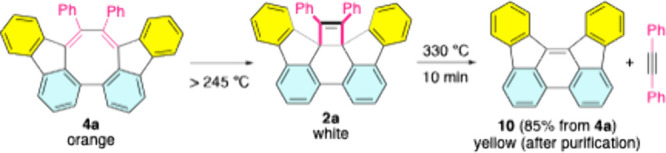
TR of **4a** (Solid Sample) and Thermal Fragmentation of **2a**

Since **4a** (orange) and **2a** (white) exhibit distinct colors (Figure S2), their thermal rearrangement and subsequent fragmentation were tracked using a melting point apparatus (Scheme 3 and Supporting Information). When heated above 245 °C, **4a** underwent direct rearrangement to **2a**. Further heating beyond 286 °C caused the white solid to transition into an orange liquid accompanied by a colorless vapor, which were identified as benz­[*e*]­indeno­[1,2,3-*hi*]­acephenanthrylene (**10**)^6^ and diphenylethyne, respectively. Maintaining the reaction at 330 °C for 10 min afforded **10** with a yield of 85% (from **4a**).

Crystals of **2a**, **4a**, **5**, and **6** were obtained by the slow diffusion of methanol into dichloromethane solutions at room temperature (Table S1). Compound **2a** with a quasi-*C*_S_ point symmetry features a curved backbone fused by a cyclobutene ring (Figure 2). The deviation from ideal geometry is attributed to a short H···H contact (two yellow hydrogen atoms) of 2.14 Å, nearly matching the sum of the van der Waals radii of the two hydrogen atoms (*d*_vdW_ = 2.18 Å).^7^ The carbon–carbon σ bond (pink) is 1.61 Å in length and 1.45 Å above the gray mean-square plane composed of four blue atoms.

**Figure 2:**
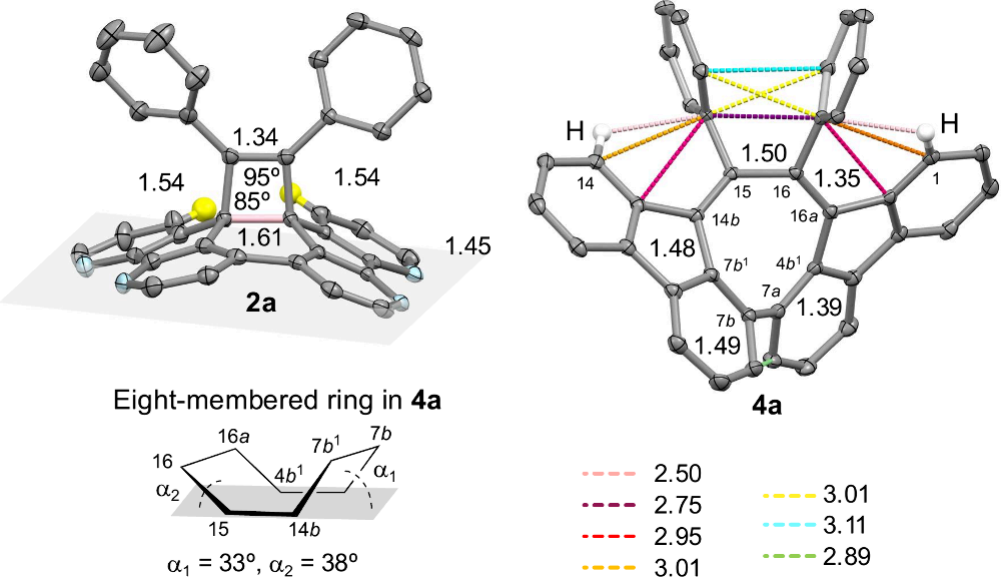
Crystallographic structures (with 50% thermal ellipsoids) and selected structural parameters (in Å) of **2a** and **4a**. Most hydrogen atoms are omitted for clarity.

**4a** adopts a *C*_2_-symmetric twisted structure, where the eight-membered ring takes on a tub shape with bending angles (α_1_ and α_2_) of 33° and 38° (Figure 2). In comparison with *C*_2_-dibenzo­[*a*,*c*]­cyclooctatetraene (DBC; α_1_ = 44°, α_2_ = 47°; bond lengths of 1.32 Å and 1.46 Å),^8^ **4a** is less twisted and has a more extended butadiene moiety (1.35 Å and 1.50 Å). The strain in **4a** is evidenced by several short nonbonded C···C (<3.20 Å vs *d*_vdW_ = 3.50 Å) and C···H (<2.55 Å vs *d*_vdW_ = 2.84 Å) contacts^7^ primarily between two phenyl substituents and nearby atoms (C1, C14, C14a, C16b, 1-H, and 14-H).

Compounds **5** and **6** each contain two independent molecules with comparable structural parameters. Both compounds display quasi-*C*_S_ symmetric geometries with a 1,2-diphenylethenyl moiety oriented perpendicularly to the molecular backbone (Figure 3). The slightly twisted backbones, confirmed by an interplanar angle of approximately 18° (A/A′ in **5** and C/C′ in **6**), can be attributed to short H···H interactions.

**Figure 3:**
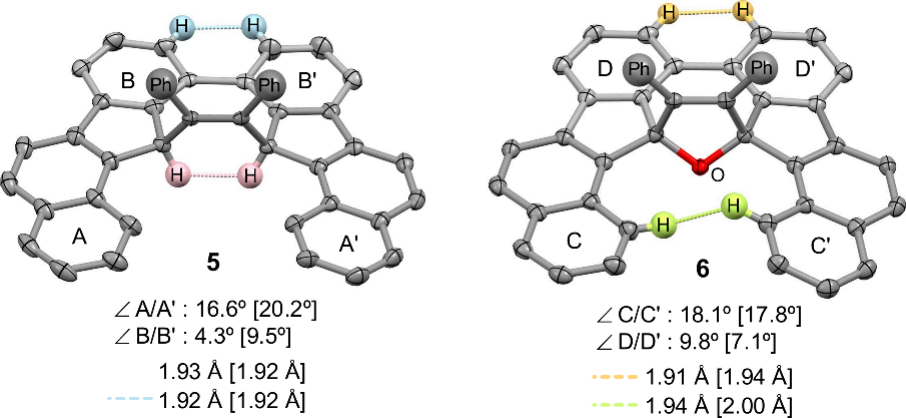
Crystallographic structures (with 50% thermal ellipsoids) and selected structural parameters of **5** and **6**. Phenyl groups and most hydrogen atoms are omitted for clarity. The structural data of the other molecule are presented in the square bracket.

Based on the experimental findings, the proposed PCCI mechanism of **3a** is outlined in Scheme 4. Initially, Pd(0) cleaves the carbon–carbon σ bond in biphenylene to generate 9-palladafluorene **7** (route 1),^4b^ which could simply undergo an alkyne insertion to yield a B*e*AP-type product **11** or proceed through several steps to form **4a**. Because **11** was not observed in this study, the possibility of this route can be eliminated. Alternatively, the two alkynyl groups in **3a** first coordinate with Pd(0), forming palladacyclopentadiene **8** (route 2). This cyclometalation step is analogous to the formation of cobaltacyclopentadiene, a key intermediate in the Vollhardt reaction.^9^ The Pd­(II) species in **8** activates the four-membered ring to yield complex **9**.^10^ Subsequent reductive elimination produces **4a** and regenerates Pd(0). Although the precise roles of carbonate and bicarbonate in promoting the PCCI remain elusive, it is plausible that they contribute by stabilizing Pd­(II) complexes, such as **8** and **9**.

**Scheme 4:**
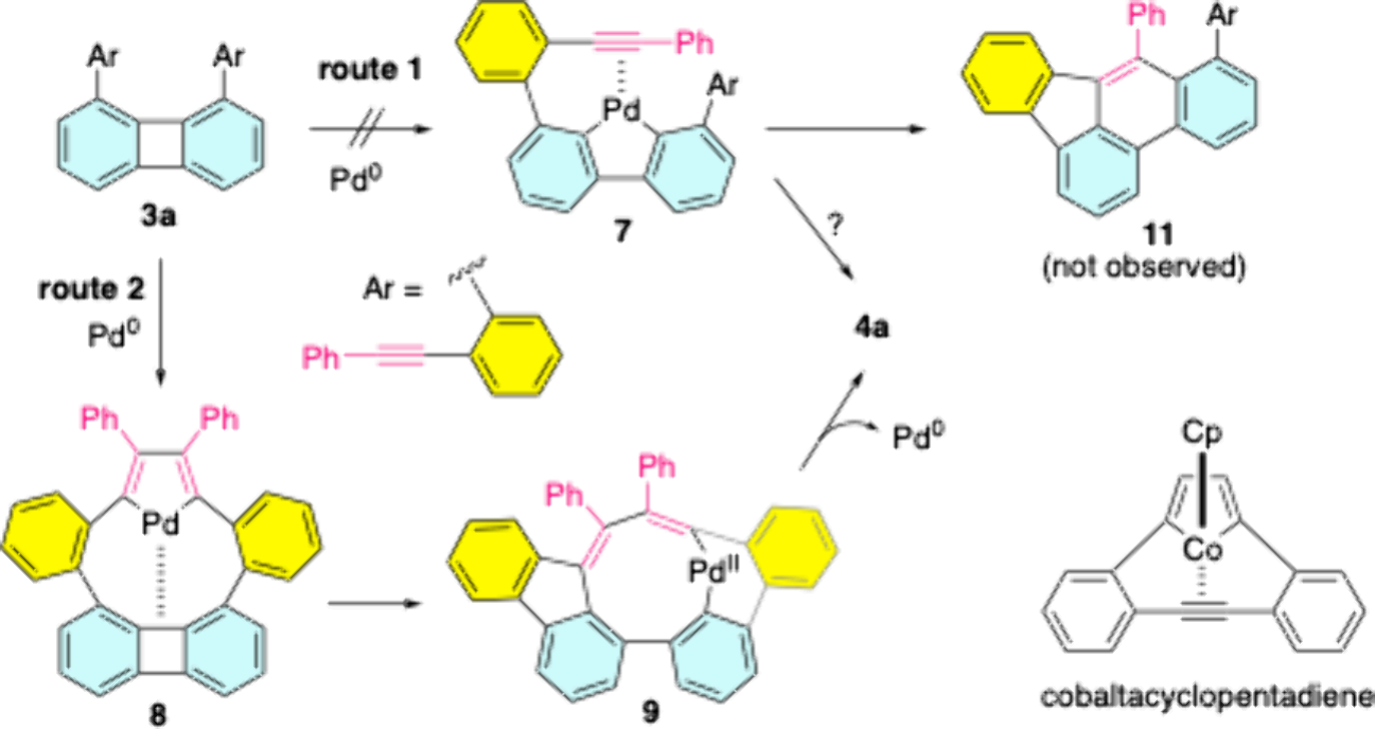
Proposed Mechanism for the PCCI

BCO/COT isomerization depends on thermal stability (or the barrier height) and the relative energies of the two compounds with BCOs typically undergoing unidirectional conversion to COTs (Scheme 5).^11^ In some cases, the thermal stability of BCOs can be enhanced by fusing benzene rings to the backbone^12^ or incorporating bulky^13^ or electron-withdrawing^14^ substituents. For example, pristine BCO exhibits a half-life (*t*_1/2_) of 14 min at 0 °C (*E*_a_ = 18.7 kcal/mol) for the isomerization to COT.^12a^ In contrast, the TR of 2*a*,10*b*-dihydrocyclobuta­[*l*]­phenanthrene (DCP) to DBC requires a significantly higher temperature (350 °C).^12d^ A notable example is 7,8-di-*tert*-butyl-substituted BCO (DBBCO in Scheme 5), which resists TR due to steric clashes between two *tert*-butyl groups in the isomerized product (1,2-di*-tert*-butyl-substituted COT). This assertion is supported by the remarkable instability of 1,2,5,6-tetra-*tert*-butyl-COT (1,2,5,6-TBCOT), which is approximately 25 kcal/mol energetically more unfavorable than its analog 1,4,5,8-TBCOT.^15^ Cyano-substituted cyclophane (CP1-CN) is an unusual case that irreversibly converts to CP2-CN with an activation energy (*E*_a_) of 26.0 kcal/mol.^16^ However, this conversion is not reported for pristine and aldehyde-substituted cyclophanes (CP1-H and CP1-CHO, respectively).

**Scheme 5:**
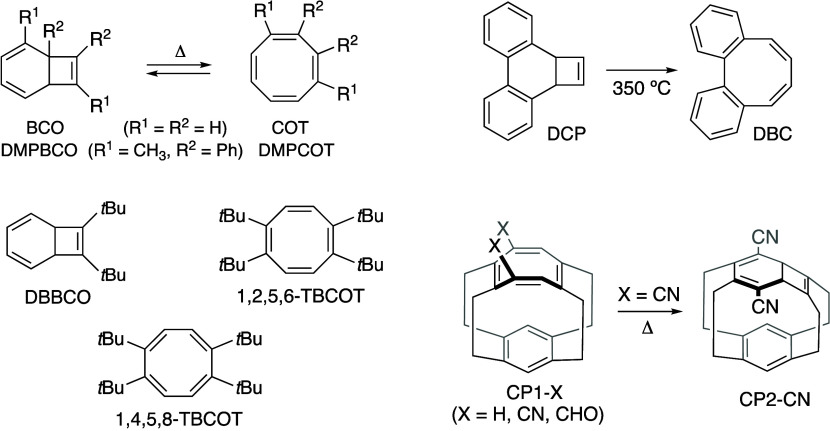
Thermal Rearrangements of BCO and COT Derivatives

Density functional theory^17^ (DFT; PW6B95D3/6–31G** //PW6B95D3/6–311++G**) reveals that **2a** is more stable than **4a** by 2.1 kcal/mol (Figure 4). The energetic instability of **4a** can be attributed to steric congestion induced by the aryl substituents, agreeing well with the interaction region indicator (*IRI,* ^*18*^ Figure 5) and structural analyses (Figure 2). Further evidence is provided by pristine compound **4l** (R^1^ = R^2^ = R^3^ = H in Scheme 1), which lacks R^1^ substituents and is more stable than **2l** by 18.2 kcal/mol. The **4a**/**2a** TR proceeds via a singlet biradical transition state TS-**a** with barriers of 27.7 kcal/mol, which aligns well with the experimental value. The TR mechanism is interpreted by *C*_2_-**4a** to quasi-*C*_S_-**2a**, serving as a representative example. The transition state TS-**a** exhibits characteristics that fulfill the necessary criteria including the alignment of the two phenyl groups in a *cis* configuration, the close distance between C14b and C16a (2.95 Å), and the alternating bond lengths within the eight-membered ring. The formal C=C and C–C bonds within the butadienyl fragment (C14b–C15–C16-C16a) undergo elongation and contraction, respectively, with the most pronounced change observed in the C15–C16 bond (from 1.48 Å to 1.39 Å).

**Figure 4:**
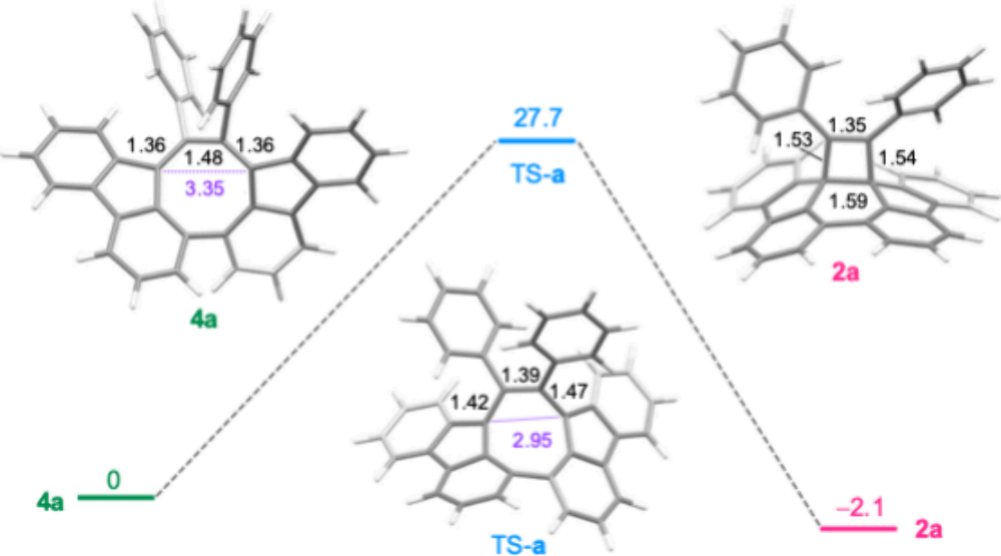
Energy profiles for **4a**/**2a** TRs (Δ*G* in kcal/mol).

**Figure 5:**
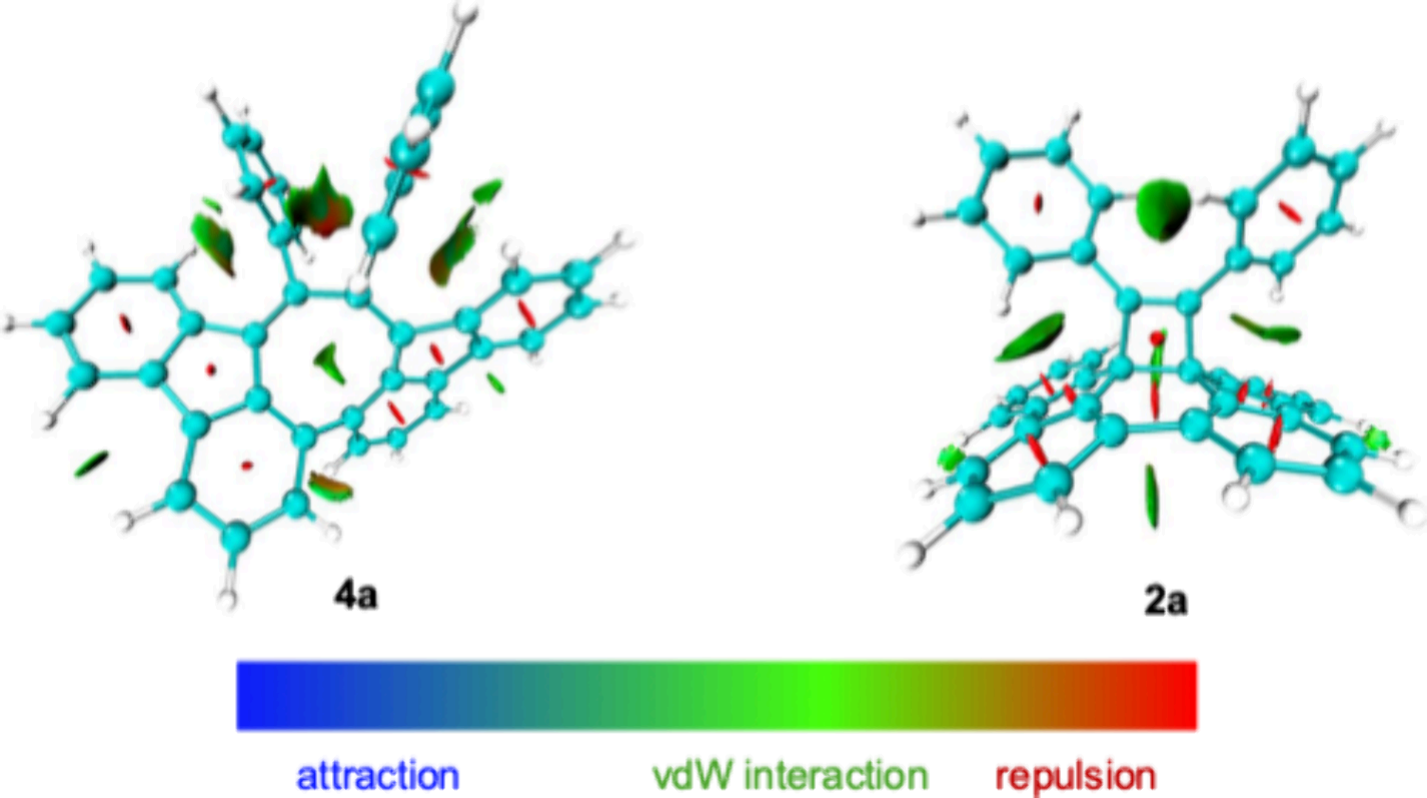
IRI isosurface plots for **2a** and **4a**. These figures were generated using Multiwfn.^18^

To explore the instability of **4k**, its structure was determined by DFT calculations and exhibited a twisted conformation with *C*_2_ symmetry. Compared to **4a**, the steric congestion in **4k** manifests in a more pronounced distortion of the eight-membered ring, larger bending angles (α_1_, α_2_: 39° and 45° versus 34° and 40°, respectively), and an increased torsion angle (C14–C14a–C14b–C15: 44° versus 30°, see Table S2). The formation of **5** and **6** likely serves as a simple method to relieve the strain of **4k**.

In conclusion, this study unveils a novel reaction pathway for biphenylene and substantiates the unconventional **4**/**2** TR governed by the steric influence of aryl substituents. The palladium-catalyzed cascade reaction of DBB and **1** initially formed Suzuki product **3**, which consecutively underwent the PCCI to yield **4**. The progression of the subsequent **4**/**2** TR via a singlet diradical transition state is feasible for most cases due to the greater energetic stability of **2** compared to **4**. The PCCI process was rendered unfavorable by steric congestion stemming from bulky R^1^ substituents, such as 2-tolyl or 2-chlorophenyl groups, as well as the aryl moiety flanking the biphenylene core.

## Supplementary Material



## Data Availability

The data underlying this study are available in the published article and its Supporting Information.

## Notes

Experimental procedures, characterization data, computational studies, and X-ray crystallography data (PDF)
