# Supplementary material for: 1,8-Di(2-ethynylaryl)biphenylenes: Palladium-Catalyzed Intramolecular Cycloisomerization and Subsequent Thermal Rearrangement
Source: Org Lett. 2025 Nov 11;27(46):12886–91. doi: 10.1021/acs.orglett.5c04302 (PMC12645571; doi:10.1021/acs.orglett.5c04302)

Supporting Information

for

1,8-Di(2-ethynylaryl)biphenylenes: Palladium-Catalyzed Intramolecular Cycloisomerization and Subsequent Thermal Rearrangement

Hsiang-Han Chen, Chih-Hsuan Liu, Wei-Ting Ou, Kuan-Hsun Huang, Chia-Jung Yang, Mu-Jeng Cheng, Yao-Ting Wu*

Department of Chemistry, National Cheng Kung University, 70101 Tainan, Taiwan.

Contents

**A**	General	SI-1
**B**	Preparations	
**B1**	1-Bromo-2-(arylethynyl)arenes **S1**	SI-1
**B2**	2-(Arylethynyl)arylboronic Acids **1**	SI-4
**B3**	Compounds **2–6**, **10**	SI-7
**C**	Thermal Rearrangement of **4a**	SI-13
**D**	Structural Analyses	SI-17
**E**	Computational Details	SI-20
**F**	NMR spectra	SI-31

A. General

^{1}H NMR spectra were recorded on Brucker 400, 500 and 700 MHz spectrometers. ^{13}C NMR spectra were recorded on Brucker 125 and 175 MHz NMR spectrometers. High-resolution mass spectra (HRMS) were obtained on JEOL JMS-700, JEOL AccuTOF GCx-plus or Bruker New ultrafleXtreme Mass Spectrometers. Melting points were determined with a Büchi melting point apparatus B545 and are uncorrected. Single-crystal X-ray diffraction was performed on a Bruker D8 Venture Single-Crystal X-Ray Diffractometer, and the data were collected and processed by using a PHOTON III detector. Steady-state absorption spectra are measured by a Perkin Elmer (Lambda 950) spectrophotometer.

B. Preparations

Unless otherwise specified, an oil bath was employed as the thermal source for a heating reaction.

B1. 1-Bromo-2-(arylethynyl)arenes **S1**

General procedure for synthesis of 1-bromo-2-(arylethynyl)arenes **S1** (GP1): A mixture of 1-bromo-2-iodoarene (15.9 mmol), $Pd(PPh_3)_2Cl_2$ (361 mg, 0.5 mmol, 3.1 mol %), CuI (0.2 g, 1.0 mmol, 6.3 mol %), THF (14 mL) and triethylamine (7 mL) in a Pyrex bottle was degassed with nitrogen for 5 min. Subsequently, an alkyne (20.8 mmol, 1.3 equiv.) was added to the solution. The sealed bottle was kept at room temperature for 16 h. Hydrochloric acid (2 N, 20 mL) was carefully added to the solution in an ice bath and the aqueous phase was extracted with ethyl acetate (2×20 mL). The combined extracts were dried over anhydrous $MgSO_4$ and the solvent of filtrate was removed under reduced pressure. The residue was subjected to chromatography on silica gel.

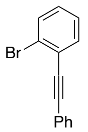

1-bromo-2-(phenylethynyl)benzene (**S1a**). Following GP1, **S1a** (3.8 g, 93%) was prepared from 2-bromo-1-iodobenzene (4.5 g, 15.9 mmol) and phenylacetylene (2.1 g, 20.8 mmol), and purified by column chromatography (SiO_2, *n*-hexane). Pale yellow oil. R_f = 0.8 (*n*-hexane). ^{1}H NMR (400 MHz, $CDCl_3$, ppm): δ = 7.64–7.55 (m, 4H), 7.38–7.35 (m, 3H), 7.30 (t, *J* = 7.6 Hz, 1H), 7.18 (td, *J* = 7.6, 1.6 Hz, 1H). Spectral data match those reported previously.[S1]

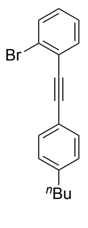

1-bromo-2-[(4-*n*-butylphenyl)ethynyl]benzene (**S1b**). Following GP1, **S1b** (4.5 g, 90%) was prepared from 2-bromo-1-iodobenzene (4.5 g, 15.9 mmol) and 4-*n*-butylphenylethyne (3.3 g, 20.8 mmol), and purified by column chromatography (SiO_2, *n*-hexane). Pale yellow oil. R_f = 0.75 (*n*-hexane). ^{1}H NMR (400 MHz, $CDCl_3$, ppm): δ = 7.61 (d, *J* = 7.8 Hz, 1H), 7.55 (dd, *J* = 7.7, 1.6 Hz, 1H), 7.49 (d, *J* = 8.1 Hz, 2H), 7.28 (t, *J* = 7.4 Hz, 1H), 7.19–7.14 (m, 3H), 2.63 (t, *J* = 7.6 Hz, 2H), 1.61 (quint, *J* = 7.2 Hz, 2H), 1.36 (sex, *J* = 7.5 Hz, 2H), 0.93 (t, *J* = 7.3 Hz, 3H). Spectral data match those reported previously.[S2]

(S1) Liu, S.; Chen, W.; Yan, C.; Zhou, F.; Zhou, Z.; Wang, M.; Zhou, G., A Comprehensive Study on Redox Behavior and Halogen Exchange in Telluropyran Derivatives. *Eur. J. Org. Chem.* **2023**, *26*, e20230033.

(S2) Huang, H. C.; Hsieh, Y. C.; Lee, P. L.; Lin, C. C.; Ho, Y. S.; Shao, W. K.; Hsieh, C. T.; Cheng, M. J.; Wu, Y. T. Highly Distorted Multiple Helicenes: Syntheses, Structural Analyses, and Properties. *J. Am. Chem. Soc.* **2023**, *145*, 10304.

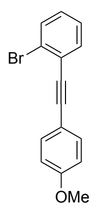

1-bromo-2-[(4-methoxyphenyl)ethynyl]benzene (**S1c**). Following GP1, **S1c** (4.1 g, 90%) was prepared from 2-bromo-1-iodobenzene (4.5 g, 15.9 mmol) and 4-(methoxyphenyl)-ethyne (2.7 g, 20.8 mmol), and purified by column chromatography (SiO_2, *n*-hexane to *n*-hexane/CH_2Cl_2 8:1). White solid. R_f = 0.6 (*n*-hexane/CH_2Cl_2 8:1). ^{1}H NMR (400 MHz, $CDCl_3$): δ = 7.60 (dd, J = 8.0, 1.2 Hz, 1H), 7.55–7.50 (m, 3H), 7.28 (td, J = 7.6, 1.2 Hz, 1H), 7.15 (td, J = 7.6, 2.0 Hz, 1H), 6.91–6.87 (m, 2H), 3.84 (s, 3H). Spectral data match those reported previously.[S3]

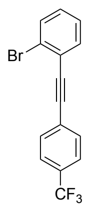

1-bromo-2-{[4-(trifluoromethyl)phenyl]ethynyl)}benzene (**S1d**). Following GP1, **S1d** (4.6 g, 88%) was prepared from 2-bromo-1-iodobenzene (4.5 g, 15.9 mmol) and 4-(trifluoro-methyl)phenylethyne (3.5 g, 20.8 mmol), and purified by column chromatography (SiO_2, *n*-hexane to *n*-hexane/CH_2Cl_2 8:1). Pale yellow solids. R_f = 0.65 (*n*-hexane/CH_2Cl_2 8: 1). ^{1}H NMR (400 MHz, $CDCl_3$): δ = 7.68 (d, J = 7.9 Hz, 2H), 7.65–7.60 (m, 3H), 7.58 (d, J = 7.6 Hz, 1H), 7.31 (t, J = 7.4 Hz, 1H), 7.21 (t, J = 7.7 Hz, 1H). Spectral data match those reported previously.[S4]

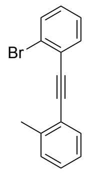

1-bromo-2-[(2-tolyl)ethynyl]benzene (**S1e**) Following GP1, **S1e** (3.9 g, 91%) was prepared from 2-bromo-1-iodobenzene (4.5 g, 15.9 mmol) and 2-tolylethyne (2.4 g, 20.8 mmol), and purified by column chromatography (SiO_2, *n*-hexane). White solid. R_f = 0.6 (*n*-hexane). ^{1}H NMR (400 MHz, $CDCl_3$): δ = 7.62 (dd, J = 8.0, 1.2 Hz, 1H), 7.57 (dd, J = 7.6, 1.6 Hz, 1H), 7.55 (d, J = 7.6 Hz, 1H), 7.30 (td, J = 7.6, 1.2 Hz, 1H), 7.27–7.24 (m, 2H), 7.18 (td, J = 7.6, 1.6 Hz, 2H), 2.58 (s, 3H). Spectral data match those reported previously.[S5]

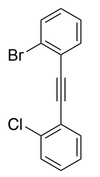

1-bromo-2-[(2-chlorophenyl)ethynyl]benzene (**S1f**). Following GP1, **S1f** (4.3 g, 93%) was prepared from 2-bromo-1-iodobenzene (4.5 g, 15.9 mmol) and 2-chlorolphenylethyne (2.8 g, 20.8 mmol), and purified by column chromatography (SiO_2, *n*-hexane). White solid. R_f = 0.6 (*n*-hexane). ^{1}H NMR (400 MHz, $CDCl_3$): δ = 7.64–7.59 (m, 3H), 7.44 (d, J = 7.6 Hz, 1H), 7.33–7.23 (m, 3H), 7.21 (t, J = 7.2 Hz, 1H). Spectral data match those reported previously.[S6]

(S3) Chen, M.; Su, N.; Deng, T.; Wink, D. J.; Zhao, Y.; Driver, T. G. Controlling the Selectivity Patterns of Au-Catalyzed Cyclization-Migration Reactions. *Org. Lett.* **2019**, *21*, 1555.

(S4) Ammon, E.; Khomutetckaia, A.; Villinger, A.; Ehlers, P.; Langer, P. Serendipitous Discovery of Pd-Catalyzed Intra-molecular Cyclization of ortho-Bromo(hetero)aryl-Substituted (Hetero)Aryl-1,2-diketones: Applications in The Synthesis of Carba- and Heterocyclic Benzoin Derivatives. *Tetrahedron* **2023**, *135*, 133335.

(S5) Mackenroth, A. V.; Antoni, P. W.; Shiri, F.; Bendel, C.; Mayer, C.; Gross, J. H.; Rominger, F.; Rudolph, M.; Ariafard, A.; Hashmi, A. S. K. Gold-Catalysed Intramolecular Reaction of Alkynes with Sulfoximines Acting as N-and O-Transfer Reagents. *Angew. Chem. Int. Ed.* **2025**, *64*, e202420360

(S6) Orita, A.; Miyamoto, K.; Nakashima, M.; Ye, F.; Otera, J. Double Elimination Protocol for Convenient Synthesis of Dihalodiphenylacetylenes: Versatile Building Blocks for Tailor-Made Phenylene-Ethynylenes. *Adv. Synth. Catal.* **2004**, *346*, 767.

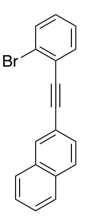

2-[(2-bromophenyl)ethynyl]naphthalene (**S1g**). Following GP1, **S1g** (4.0 g, 82%) was prepared from 2-bromo-1-iodobenzene (4.5 g, 15.9 mmol) and 2-ethynylnaphthalene (3.2 g, 20.8 mmol), and purified by column chromatography (SiO_2, *n*-hexane to *n*-hexane/CH_2Cl_2 5:1). Pale yellow solids. R_f = 0.6 (*n*-hexane/CH_2Cl_2 5:1). ^{1}H NMR (400 MHz, $CDCl_3$): δ = 8.17–8.12 (m, 1H), 7.88–7.82 (m, 3H), 7.70–7.60 (m, 3H), 7.57–7.49 (m, 2H), 7.32 (td, *J* = 7.6, 1.8 Hz, 1H), 7.20 (td, *J* = 7.6, 1.3 Hz, 1H). Spectral data match those reported previously.[S7]

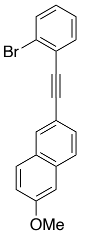

2-[(2-bromophenyl)ethynyl]-6-methoxynaphthalene (**S1h**). Following GP1, **S1h** (4.8 g, 89%) was prepared from 2-bromo-1-iodobenzene (4.5 g, 15.9 mmol) and 2-ethynyl-6-methoxy-naphthalene (3.8 g, 20.8 mmol), and purified by column chromatography (SiO_2, *n*-hexane to *n*-hexane/CH_2Cl_2 8:1). Pale yellow solids. R_f = 0.6 (*n*-hexane/CH_2Cl_2 5:1). ^{1}H NMR (400 MHz, $CDCl_3$): δ = 8.03 (s, 1H), 7.72 (t, *J* = 8.2 Hz, 2H), 7.64 (dd, *J* = 8.0, 1.2 Hz, 1H), 7.59 (d, *J* = 8.0 Hz, 2H), 7.31 (td, *J* = 7.6, 1.2 Hz, 1H), 7.18 (t, *J* = 7.6 Hz, 2H), 7.13 (d, *J* = 2.4 Hz, 1H), 3.94 (s, 3H). Spectral data match those reported previously.[S8]

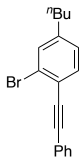

2-bromo-4-*n*-butyl-1-(phenylethynyl)benzene (**S1i**). Following GP1, **S1i** (4.3 g, 82%) was prepared from 2-bromo-4-*n*-butyl-1-iodobenzene (5.39 g, 15.9 mmol) and phenylacetylene (2.1 g, 20.8 mmol), and purified by column chromatography (SiO_2, *n*-hexane). A white solid. R_f = 0.75 (*n*-hexane/CH_2Cl_2 8:1). Note: The isolated product contained a minor impurity of 1,4-diphenylbutadiyne (5%), which could not be removed from **S1i** by column chromatography. Consequently, the collected material was employed directly in the subsequent transformation. ^{1}H NMR (400 MHz, $CDCl_3$): δ = 7.62–7.58 (m, 2H), 7.47 (s, 1H), 7.40–7.36 (m, 4H), 7.13 (dd, *J* = 8.0, 1.2 Hz, 1H), 2.62 (t, *J* = 7.6 Hz, 2H), 1.62 (quint, *J* = 7.6 Hz, 2H), 1.38 (sex, *J* = 7.2 Hz, 2H), 0.96 (t, *J* = 7.2 Hz, 3H). ^{13}C NMR (125 MHz, $CDCl_3$, plus DEPT, ppm): δ = 145.1 (C_{quat}), 133.0 (CH), 132.4 (CH), 131.6 (CH), 128.4 (CH), 128.3 (CH), 127.3 (CH), 125.5 (C_{quat}), 123.2 (C_{quat}), 122.5 (C_{quat}), 93.1 (C_{quat}), 88.2 (C_{quat}), 35.2 (CH_2), 33.1 (CH_2), 22.2 (CH_2), 13.9 (CH_3). HRMS (EI) *m/z*: $[M]^+$ calcd for $C_{18}H_{17}Br$, 312.0508; found: 312.0507.

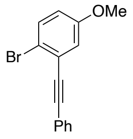

1-bromo-4-methoxy-2-(phenylethynyl)benzene (**S1j**). Following GP1, **S1j** (4.0 g, 88%) was prepared from 1-bromo-2-iodo-4-methoxybenzene (5.0 g, 16.0 mmol) and phenylacetylene (2.1 g, 20.8 mmol), and purified by column chromatography (SiO_2, *n*-hexane to *n*-hexane/CH_2Cl_2 5:1). Pale yellow Oil. R_f = 0.70 (*n*-hexane/CH_2Cl_2 5:1). ^{1}H NMR (400 MHz, $CDCl_3$): δ = 7.61–7.57 (m, 2H), 7.48 (d, *J* = 9.2 Hz, 1H), 7.39–7.34 (m, 3H), 7.10 (d, *J* = 3.2 Hz, 1H), 6.77 (dd, *J* = 8.8, 3.2 Hz, 1H), 3.81 (s, 3H). Spectral data match those reported previously.[S3]

(S7) Shinde, P. S.; Shaikh, A. C.; Patil, N. T. Efficient Access to Alkynylated Quinalizinones via The Gold(I)-Catalyzed Aminoalkynylation of Alkynes. *Chem. Comm.* **2016**, *52*, 8152.

(S8) Verma, A. K.; Jha, R. R.; Chaudhary, R.; Tiwari, R. K.; Reddy, K. S.; Danodia, A. Copper-Catalyzed Tandem Synthesis of Indolo-, Pyrrolo[2,1-*a*]isoquinolines, Naphthyridines and Bisindolo/pyrrolo[2,1-*a*]isoquinolines via Hydroamination of ortho-Haloarylalkynes Followed by C-2 Arylation. *J. Org. Chem.* **2012**, *77*, 8191.

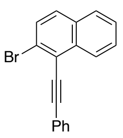

2-bromo-1-(phenylethynyl)naphthalene (**S1k**). A mixture of 2-bromonaphthalen-1-yl trifluoromethanesulfonate (1.0 g, 2.87 mmol), $Pd(PPh_3)_2Cl_2$ (101 mg, 5 mol %), CuI (54.5 mg, 10 mol %), DMF (5.7 mL) and diisopropylamine (5.7 mL) in a Pyrex bottle was degassed with nitrogen for 5 min. Subsequently, phenylacetylene (0.4 g, 3.9 mmol) was added to the solution. The sealed bottle was kept at room temperature for 16 h. Hydrochloric acid (2 N, 10 mL) was carefully added to the solution in an ice bath and the aqueous phase was extracted with ethyl acetate (2×10 mL). The combined extracts were dried over anhydrous $MgSO_4$ and the solvent of filtrate was removed under reduced pressure. The residue was purified by chromatography on silica gel, eluting with hexane to give **S1k** (0.43 g, 49%) as a pale yellow oil. R_f = 0.6 (hexane). ^{1}H NMR (500 MHz, $CDCl_3$): δ = 8.43 (d, *J* = 8.0 Hz, 1H), 7.83 (d, *J* = 8.0 Hz, 1H), 7.72–7.70 (m, 2 H), 7.68 (s, 2H), 7.62 (ddd, *J* = 8.0, 7.0, 1.5 Hz, 1H), 7.54 (ddd, *J* = 8.0, 6.5, 1.5 Hz, 1H), 7.44–7.38 (m, 3 H). ^{13}C NMR (125 MHz, $CDCl_3$, plus DEPT, ppm): δ = 134.4 (C_{quat}), 131.8 (CH), 129.6 (CH), 129.3 (CH), 128.8 (CH), 128.5 (CH×2), 128.3 (CH), 127.7 (CH), 126.6 (CH), 126.4 (CH), 124.8 (C_{quat}), 123.1 (C_{quat}), 122.4 (C_{quat}), 99.3 (C_{quat}), 86.5 (C_{quat}). HRMS (FAB) *m/z*: $[M]^+$ calcd for $C_{18}H_{11}Br$, 306.0039; found: 306.0051.

B2. 2-(Arylethynyl)arylboronic Acids **1**

General procedure for preparation of 2-(arylethynyl)arylboronic acid **1** (GP2). To a solution of 1-bromo-2-(arylethynyl)arene **S1** (5.0 mmol) in THF (25 mL) at −78 °C was added *n*-butyllithium (2.8 mL, 2.50 M in hexane, 7.0 mmol, 1.4 equiv.). After being stirred at the same temperature for 1 h, the solution was treated with trimethyl borate (1.7 mL, 15 mmol, 3.0 equiv.). The reaction mixture was warmed up to room temperature and stirred overnight. The solution was quenched by hydrochloric acid (2 N, 20 mL) at 0 °C. The organic layer was separated, and the aqueous phase was extracted with ethyl acetate (3×20 mL). The combined organic layers were dried over anhydrous $MgSO_4$. The solvent of the filtrate was removed under reduced pressure, and the residue was triturated with hexane (3×10 mL) to give compound **1**.

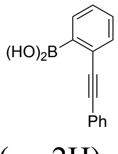

2-(phenylethynyl)phenylboronic acid (**1a**). Following GP2, **1a** (1.6 g, 93%) was prepared from **S1a** (2.0 g, 7.8 mmol). White solid. m.p. 109–110 °C. ^{1}H NMR (400 MHz, $CDCl_3$, ppm): δ = 8.01 (dd, *J* = 7.2, 1.6 Hz, 1H), 7.60 (ddd, *J* = 7.6, 1.2, 0.4 Hz, 1H), 7.58–7.53 (m, 2H), 7.45 (td, *J* = 7.6, 1.6 Hz, 1H), 7.43–7.38 (m, 4H), 5.68 (s, 2H). Spectral data match those reported previously.[S9]

(S9) Benhamou, L.; Walker, D. W.; Bucar, D. K.; Aliev, A. E.; Sheppard, T. D. Synthesis of Substituted Benzooxaborinin-1-ols via Palladium-Catalysed Cyclisation of Alkenyl- and Alkynyl-boronic acids. *Org. Biomol. Chem.*, **2016**, *14*, 8039.

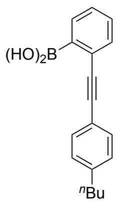

2-[(4-*n*-butylphenyl)ethynyl]phenylboronic acid (**1b**). Following GP2, **1b** (1.3 g, 90%) was prepared from **S1b** (1.6 g, 5.0 mmol). White solid, m.p. 75–76 °C. ^{1}H NMR (400 MHz, $CDCl_3$, ppm): δ = 8.01 (d, *J* = 7.6 Hz, 1H), 7.58 (d, *J* = 7.6 Hz, 1H), 7.47–7.43 (m, 3H), 7.39 (td, *J* = 7.6, 1.6 Hz, 1H), 7.20 (d, *J* = 8.5 Hz, 2H), 5.67 (s, 2H), 2.64 (t, *J* = 7.6 Hz, 2H), 1.61 (quint, *J* = 7.6 Hz, 2H), 1.36 (sex, *J* = 7.6 Hz, 2H), 0.94 (t, *J* = 7.6 Hz, 3H). Spectral data match those reported previously.[S2]

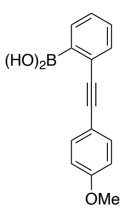

2-(4-anisylethynyl)phenylboronic acid (**1c**). Following GP2, **1c** (1.2 g, 92%) was prepared from **S1c** (1.4 g, 5.0 mmol). White solid, m.p. 113−114 °C. ^{1}H NMR ($CDCl_3$, 500 MHz): δ = 8.00 (dd, *J* = 7.6 Hz, 1.2 Hz, 1H), 7.57 (dd, *J* = 7.6, 1.2 Hz, 1H), 7.50–7.47 (m, 2H), 7.45 (td, *J* = 7.6, 1.6 Hz, 1H), 7.38 (td, *J* = 7.6, 1.2 Hz, 1H), 6.93−6.89 (m, 2H), 5.73 (s, 2H), 3.85 (s, 3H). Spectral data match those reported previously.[S10]

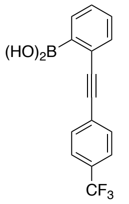

2-[4-(trifluoromethyl)phenylethynyl]phenylboronic acid (**1d**). Following GP2, **1d** (1.3 g, 88%) was prepared from **S1d** (1.6 g, 5.0 mmol). White solid, m.p. 112−113 ºC. ^{1}H NMR (400 MHz, $CDCl_3$, ppm): δ = 8.01 (dd, *J* = 7.6, 1.6 Hz, 1H), 7.65 (s, 4H), 7.61 (dd, *J* = 7.6, 1.6 Hz, 1H), 7.49 (td, *J* = 7.2, 1.6 Hz, 1H), 7.44 (td, *J* = 7.2, 1.6 Hz, 1H), 5.60 (s, 2H). ^{13}C NMR (125 MHz, $CDCl_3$, plus DEPT, ppm): δ = 135.7 (CH), 132.8 (CH), 131.8 (CH), 130.9 (CH), 128.9 (CH), 128.4 (C_{quat}), 125.9 (C_{quat}), 125.6 (q, *J* = 3.6 Hz, CH), 92.0 (C_{quat}), 91.8 (C_{quat}). Due to insufficient signal intensity, two C_{quat} cannot be assigned. HRMS (EI) *m*/*z*: $[M]^+$ calcd for $C_{15}H_{10}BF_3O_2$, 290.0720; found: 290.0728.

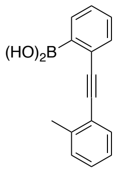

2-(2-tolylethynyl)phenylboronic acid (**1e**). Following GP2, **1e** (1.1 g, 91%) was prepared from **S1e** (1.4 g, 5.0 mmol). White solid, m.p. 101−102 °C. ^{1}H NMR (400 MHz, $CDCl_3$, ppm): δ = 8.01 (dd, *J* = 7.6, 1.2 Hz, 1H), 7.60 (dd, *J* = 7.6, 1.2 Hz, 1H), 7.51 (d, *J* = 7.2 Hz, 1H), 7.47 (td, *J* = 7.2, 1.6 Hz, 1H), 7.41 (td, *J* = 7.2, 1.6 Hz, 1H), 7.25−7.32 (m, 2H), 7.21 (td, *J* = 7.6, 2.0 Hz, 1H), 5.68 (s, 2H), 2.53 (s, 3H). Spectral data match those reported previously.[S11]

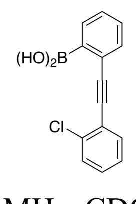

2-[(2-chlorophenyl)ethynyl]phenylboronic acid (**1f**). Following GP2, **1f** (1.2 g, 91%) was prepared from **S1f** (1.5 g, 5.0 mmol). White solid, m.p. 103−104 ºC. ^{1}H NMR (400 MHz, $CDCl_3$, ppm): δ = 8.04 (dd, *J* = 7.6, 1.2 Hz, 1H), 7.64 (d, *J* = 7.6 Hz, 1H), 7.60 (dd, *J* = 7.6, 2.4 Hz, 1H), 7.35−7.26 (m, 2H), 7.50−7.41 (m, 3H), 5.78 (s, 2H). ^{13}C NMR (125 MHz, $CDCl_3$, plus DEPT, ppm): δ = 135.8 (CH), 135.5 (C_{quat}), 133.5 (CH), 133.1 (CH), 130.8 (CH), 130.0 (CH), 129.4 (CH), 128.7 (CH), 126.76 (CH), 126.3 (C_{quat}), 122.2 (C_{quat}), 94.7 (C_{quat}), 90.2

(S10) Körner, C.; Starkov, P.; Sheppard, T.D. An Alternative Approach to Aldol Reactions: Gold-Catalyzed Formation of Boron Enolates from Alkynes. *J. Am. Chem. Soc.* **2010**, *132*, 5968.

(S11) Hsieh, Y. C.; Wu, C. F.; Chen, Y. T.; Fang, C. T.; Wang, C. S.; Li, C. H.; Chen, L. Y.; Cheng, M. J.; Chueh, C. C.; Chou, P. T.; Wu, Y. T. 5,14-Diaryldiindeno[2,1-*f*:1',2'-*j*]picene: A New Stable [7]Helicene with a Partial Biradical Character. *J. Am. Chem. Soc.* **2018**, *140*, 14357.

(C_{quat}). HRMS (FAB) *m*/*z*: $[M]^+$ calcd for $C_{14}H_{10}BClO_2$, 256.0457; found: 256.0465.

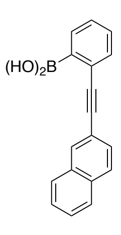

2-[(2-naphthyl)ethynyl]phenylboronic acid (**1g**). Following GP2, **1g** (1.3 g, 88%) was prepared from **S1g** (1.7 g, 5.0 mmol). White solid, m.p. 192−193 °C. ^{1}H NMR (400 MHz, $CDCl_3$, ppm): δ = 8.08 (d, *J* = 1.6 Hz, 1H), 8.03 (dd, *J* = 7.6, 1.6 Hz, 1H), 7.86−7.82 (m, 3H), 7.64 (dd, *J* = 7.6, 0.8 Hz, 1H), 7.58 (dd, *J* = 8.4, 1.6 Hz, 1H), 7.55−7.51 (m, 2H), 7.49 (td, *J* = 7.6, 1.6 Hz, 1H), 7.42 (td, *J* = 7.6, 1.6 Hz, 1H), 5.75 (s, 2H). Spectral data match those reported previously.[S7]

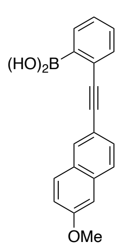

2-[(7-methoxy-2-naphthyl)ethynyl]phenylboronic acid (**1h**). Following GP2, **1h** (1.3 g, 88%) was prepared from **S1h** (1.7 g, 5.0 mmol). White solid, m.p. 195−196 °C. ^{1}H NMR (400 MHz, $CDCl_3$, ppm): δ = 8.02 (d, *J* = 7.2, 0.8 Hz, 1H), 8.00 (s, 1H), 7.74 (d, *J* = 8.8 Hz, 2H), 7.62 (d, *J* = 7.6 Hz, 1H), 7.54 (dd, *J* = 8.8, 2.0 Hz, 1H), 7.48 (td, *J* = 7.6, 1.6 Hz, 1H), 7.41 (td, *J* = 7.6, 1.2 Hz, 1H), 7.19 (dd, *J* = 9.0, 2.4 Hz, 1H), 7.14 (d, *J* = 2.4 Hz, 1H), 5.76 (s, 2H), 3.94 (s, 3H). ^{13}C NMR (125 MHz, $CDCl_3$, plus DEPT, ppm): δ = 158.7 (C_{quat}), 135.6 (CH), 134.5 (C_{quat}), 128.4 (C_{quat}), 132.5 (CH), 131.5 (CH), 130.8 (CH), 129.5 (CH), 128.5 (CH), 128.2 (CH), 127.2 (CH), 127.0 (C_{quat}), 119.7 (CH), 116.7 (C_{quat}), 105.9 (CH), 94.2 (C_{quat}), 89.4 (C_{quat}), 55.4 (CH_3). HRMS (FAB) *m*/*z*: $[M]^+$ calcd for $C_{19}H_{15}BO_3$, 302.1109; found: 302.1117.

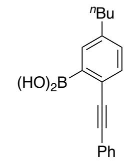

5-*n*-butyl-2-(phenylethynyl)phenylboronic acid (**1i**). Following GP2, **1i** (1.3 g, 90%) was prepared from **S1i** (1.6 g, 5.0 mmol). White solid, m.p. 78−79 °C. ^{1}H NMR (400 MHz, $CDCl_3$, ppm): δ = 7.82 (d, *J* = 2.0 Hz, 1H), 7.55−7.50 (m, 3H), 7.39−7.36 (m, 3H), 7.28 (dd, *J* = 8.0, 2.0 Hz, 1H), 5.68 (s, 2H), 2.65 (t, *J* = 7.6 Hz, 2H), 1.62 (quint, *J* = 7.2 Hz, 2H), 1.36 (sex, *J* = 7.2 Hz, 2H), 0.93 (t, *J* = 7.2 Hz, 3H). ^{13}C NMR (125 MHz, $CDCl_3$, plus DEPT, ppm): δ = 143.4 (C_{quat}), 135.7 (CH), 132.6 (CH), 131.5 (CH), 131.0 (CH), 128.9 (CH), 128.6 (CH), 123.8 (C_{quat}), 122.1 (C_{quat}), 92.9 (C_{quat}), 90.0 (C_{quat}), 35.6 (CH_2), 33.4 (CH_2), 22.4 (CH_2), 13.9 (CH_3). HRMS (EI) *m*/*z*: $[M]^+$ calcd for $C_{18}H_{19}BO_2$, 278.1473; found: 278.1477.

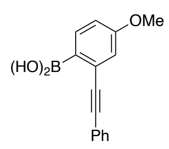

4-methoxy-2-(phenylethynyl)phenylboronic acid (**1j**). Following GP2, **1j** (1.2 g, 92%) was prepared from **S1j** (1.4 g, 5.0 mmol). White solid, m.p. 102−103°C. ^{1}H NMR (400 MHz, $CDCl_3$, ppm): δ = 7.93 (d, *J* = 8.4 Hz, 1H), 7.57−7.54 (m, 2H), 7.41−7.38 (m, 3H), 7.10 (d, *J* = 2.8 Hz, 1H), 6.95 (dd, *J* = 8.4, 2.8 Hz, 1H), 5.60−5.58 (br, 2H), 3.87 (s, 3H). Spectral data match those reported previously.[S3]

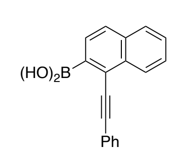

(1-(phenylethynyl)naphthalen-2-yl)boronic acid (**1k**). Following GP2, **1k** (1.1 g, 80%) was prepared from **S1k** (1.5 g, 5.0 mmol). White solid, m.p. 105−106 °C. ^{1}H NMR (400 MHz, $CDCl_3$, ppm): δ = 8.50 (d, *J* = 7.6 Hz, 1H), 8.06 (d, *J* = 8.0 Hz, 1H), 7.88 (d, *J* = 8.4 Hz, 2H), 7.69−7.67 (m, 2H), 7.65−7.57 (m, 2H), 7.46−7.44 (m, 3H). ^{13}C NMR (125 MHz, $CDCl_3$, plus DEPT, ppm): δ = 134.5 (C_{quat}), 133.3 (C_{quat}), 131.6 (CH), 130.8 (CH), 129.3 (CH), 128.7 (CH), 128.26 (CH), 128.25 (CH), 127.5 (CH), 127.0 (CH), 126.5 (CH), 125.6

(C_{quat}), 122.0 (C_{quat}), 99.0 (C_{quat}), 87.8 (C_{quat}). HRMS (FAB) *m*/*z*: $[M]^+$ calcd for $C_{18}H_{13}BO_2$: 272.1003; found: 272.1003.

B3. Compounds **2–6**, **10**

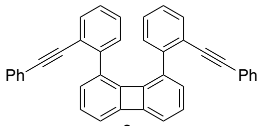

3a

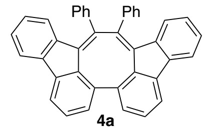

4a

1,8-bis[2-(phenylethynyl)phenyl]biphenylene (**3a**) and 15,16-diphenylcycloocta[1,2,3-*jk*:8,7,6-*j'k'*]difluorene (**4a**) [Condition A in Scheme 1]. A mixture of DBB[S12] (31 mg, 0.1 mmol), phenylboronic acid **1a** (66.6 mg, 0.3 mmol), $Pd(PPh_3)_4$ (5 mg, 4.0 μmol), K_2CO_3 (82.9 mg, 0.6 mmol), DME (0.75 mL) and water (0.25 mL) was placed in a thick-walled Pyrex tube and purged with nitrogen for 5 mins. The reaction vessel was sealed and kept at 85 ºC for 16 h. After being cooled to room temperature, water (5 mL) was added to the reaction mixture. The resulting solution was extracted with ethyl acetate (2×10 mL), and the combined organic layers were dried over anhydrous $MgSO_4$. The solvents of the filtrate were removed under reduced pressure, and the residue was purified by chromatography on silica gel, eluting with hexane/CH_2Cl_2 (8:1) to give **3a** (40 mg, 79%) and **4a** (4 mg, 8%). Crystals of **4a** were obtained by slow diffusion of methanol into its solution in DCM. **3a**: A yellow solid, m. p. 112−113 ºC. $R_f = 0.56$ (hexane/CH_2Cl_2 5:1). ^1H NMR (700 MHz, $CDCl_3$, ppm): δ = 7.14–7.09 (m, 10H), 7.04 (dd, *J* = 7.0, 1.4 Hz, 2H), 7.01 (td, *J* = 7.7, 1.4 Hz, 2H), 6.92–6.85 (m, 8H), 6.74 (dd, *J* = 7.0, 0.7 Hz, 2H). ^{13}C NMR (175 MHz, $CDCl_3$, plus DEPT, ppm): δ = 150.8 (C_{quat}×2), 140.6 (C_{quat}), 131.8 (C_{quat}), 131.4 (CH), 131.3 (CH), 130.1 (CH), 128.1 (CH), 127.9 (CH), 127.8 (CH), 127.6 (CH), 127.2 (CH), 126.6 (CH), 123.6 (C_{quat}), 121.5 (C_{quat}), 115.7 (CH), 92.3 (C_{quat}), 88.6 (C_{quat}). HRMS (FAB) *m*/*z*: $[M]^+$ calcd for $C_{40}H_{24}$, 504.1873; found: 504.1876. **4a**: Yellow solid. $R_f = 0.55$ (hexane/CH_2Cl_2 5:1). ^1H NMR (500 MHz, $CDCl_3$, ppm): δ = 7.60 (dd, *J* = 7.0, 1.0 Hz, 2H), 7.56 (d, *J* = 7.5 Hz, 2H), 7.46 (t, *J* = 7.5 Hz, 2H), 7.32 (br, 4H), 7.23–7.20 (m, 2H), 7.12 (td, *J* = 7.5, 1.0 Hz, 2H), 6.87 (br, 2H), 6.74 (dd, *J* = 8.0, 1.0 Hz, 2H), 6.64 (td, *J* = 8.0, 1.5 Hz, 2H), 5.91 (br, 2H), 5.52 (d, *J* = 8.0 Hz, 2H). ^{13}C NMR (125 MHz, $CDCl_3$, plus DEPT, ppm): δ = 143.2 (C_{quat}), 142.6 (C_{quat}), 142.5 (C_{quat}), 140.7 (C_{quat}), 140.5 (C_{quat}), 140.4 (C_{quat}), 139.08 (C_{quat}), 139.04 (C_{quat}), 131.8 (CH), 130.7 (CH), 130.1 (CH), 129.6 (CH), 127.9 (CH), 127.6 (CH), 127.3 (CH), 127.2 (CH), 125.5 (CH), 124.7 (CH), 119.2 (CH), 118.3 (CH). HRMS (FAB) *m*/*z*: $[M]^+$ calcd for $C_{40}H_{24}$, 504.1873; found: 504.1872.

General procedure for synthesis of **2** directly from DBB and arylboronic acid **1** (GP3, Condition C in Scheme 1). A mixture of DBB (31 mg, 0.1 mmol), arylboronic acid **1** (0.3 mmol, 3.0 equiv.), $Pd(PPh_3)_4$ (11 mg, 0.01 mmol, 10 mol %) and $KHCO_3$ (80 mg, 0.8 mmol, 8.0 equiv.), DME (0.75 mL) and water (0.25 mL) in a thick-walled Pyrex tube was purged with nitrogen for 5 min. The sealed tube was kept in an oil bath at 100 ºC for 40 h. After being cooled to room temperature, water (3 mL)

(S12) Kabir, S. H.; Iyoda, M. Selective Synthesis of Tetraphenylenes and Biphenylenes using Copper-Catalyzed Coupling of Zincacyclopentadienes. *Synthesis* **2000,** *2000*, 1839.

was added. The solution was extracted with ethyl acetate (2×10 mL). The combined extracts were dried over anhydrous $MgSO_4$. The solvents of the filtrate were removed under reduced pressure, and the residue was subjected to chromatography on silica gel.

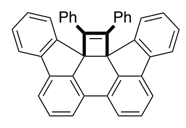

Compound **2a**. Following GP3, **2a** (36 mg, 71%) was prepared from DBB (31 mg, 0.1 mmol) and **1a** (67 mg, 0.3 mmol). The crude was purified by column chromatography (SiO_2, hexane/CH_2Cl_2 8:1). [1 mmol scale] **2a** (283 mg, 56%) was prepared from DBB (310 mg, 1 mmol) and **1a** (666 mg, 3 mmol). The crude was purified by column chromatography (SiO_2, hexane/CH_2Cl_2 6:1). Crystals of **2a** were obtained by slow diffusion of methanol into its solution in DCM. A white solid, m. p. 286–287 ºC. R_f = 0.56 (hexane/CH_2Cl_2 5:1). ^{1}H NMR (500 MHz, $CDCl_3$, ppm): δ = 8.28 (dd, J = 6.5, 1.5 Hz, 2H), 7.83 (d, J = 7.5 Hz, 2H), 7.75–7.73 (m, 2H), 7.60 (d, J = 7.5 Hz, 2H), 7.44 (t, J = 7.5 Hz, 2H), 7.39 ("t"d, J = 7.5, 1.5 Hz, 2H), 7.36 ("t"d, J = 7.5, 1.5 Hz, 2H), 7.01 (t, J = 7.0 Hz, 2H), 6.95–6.91 (m, 4H), 6.66–6.62 (m, 4H). ^{13}C NMR (125 MHz, $CDCl_3$, plus DEPT, ppm): δ = 147.0 (C_{quat}), 146.9 (C_{quat}), 145.2 (C_{quat}), 141.9 (C_{quat}), 139.2 (C_{quat}), 132.7 (C_{quat}), 131.7 (C_{quat}), 128.6 (CH), 128.1 (CH), 127.8 (CH), 127.7 (CH), 127.5 (CH), 127.1 (CH), 125.6 (CH), 122.3 (CH), 120.8 (CH), 119.0 (CH), 66.3 (C_{quat}). HRMS (FAB) m/z: $[M]^+$ calcd for $C_{40}H_{24}$, 504.1873; found: 504.1876.

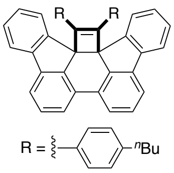

Compound **2b**. Following GP3, **2b** (47 mg, 76%) was prepared from DBB (31 mg, 0.1 mmol) and **1b** (83 mg, 0.3 mmol). The crude was purified by column chromatography (SiO_2, hexane/CH_2Cl_2 8:1). A white solid, m. p. 291−292 ºC. R_f = 0.55 (hexane/CH_2Cl_2 5:1). ^{1}H NMR (500 MHz, $CDCl_3$, ppm): δ = 8.27–8.26 (m, 2H), 7.81 (d, J = 7.5 Hz, 2H), 7.74–7.72 (m, 2H), 7.59 (dd, J = 7.5, 0.5 Hz, 2H), 7.42 (t, J = 7.5 Hz, 2H), 7.39–7.34 (m, 4H), 6.74 (d, J = 8.5 Hz, 4H), 6.57 (d, J = 8.5 Hz, 4H), 2.39 (t, J = 7.5 Hz, 4H), 1.43 (quint, J = 7.5 Hz, 4H), 1.24 (sext, J = 7.5 Hz, 4H), 0.84 (t, J = 7.5 Hz, 6H). ^{13}C NMR (125 MHz, $CDCl_3$, plus DEPT, ppm): δ = 147.3 (C_{quat}), 146.3 (C_{quat}), 145.4 (C_{quat}), 142.3 (C_{quat}), 141.9 (C_{quat}), 139.3 (C_{quat}), 131.8 (C_{quat}), 130.2 (C_{quat}), 128.5 (CH), 128.0 (CH), 127.7 (CH), 127.5 (CH), 126.9 (CH), 125.7 (CH), 122.2 (CH), 120.8 (CH), 118.9 (CH), 66.2 (C_{quat}), 35.5 (CH_2), 33.3 (CH_2), 22.5 (CH_2), 14.0 (CH_3). HRMS (EI) m/z: $[M]^+$ calcd for $C_{48}H_{40}$, 616.3125; found: 616.3123.

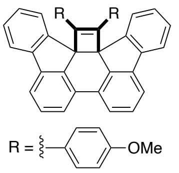

Compound **2c**. Following GP3, **2c** (34 mg, 60%) was prepared from DBB (31 mg, 0.1 mmol) and **1c** (76 mg, 0.3 mmol). The crude was purified by column chromatography (SiO_2, hexane to hexane/CH_2Cl_2 5:1). A white solid, m. p. 293−294 ºC. R_f = 0.6 (hexane/CH_2Cl_2 3:1). ^{1}H NMR (500 MHz, $CDCl_3$, ppm): δ = 8.27−8.26 (m, 2H), 7.82 (d, J = 7.5 Hz, 2H), 7.75–7.73 (m, 2H), 7.59 (dd, J = 7.5, 0.5 Hz, 2H), 7.43 (t, J = 7.5 Hz, 2H), 7.40–7.34 (m, 4H), 6.58 (d, J = 9.5 Hz, 4H), 6.47 (d, J = 9.5 Hz, 4H), 3.63 (s, 6H). ^{13}C NMR (125 MHz, $CDCl_3$, plus DEPT, ppm): δ = 158.7 (C_{quat}), 147.1 (C_{quat}), 145.7 (C_{quat}), 145.3 (C_{quat}), 145.1 (C_{quat}), 141.7 (C_{quat}), 139.0 (C_{quat}), 131.5 (C_{quat}), 128.3 (CH), 128.2 (CH), 127.5 (CH), 127.3 (CH), 125.4 (C_{quat}), 125.3 (CH), 122.1 (CH), 120.6 (CH), 118.7 (CH), 113.3 (CH), 65.9 (C_{quat}), 54.9 (CH_3). HRMS (EI) m/z: $[M]^+$ calcd for $C_{42}H_{28}O_2$, 564.2084; found: 564.2085.

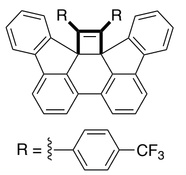

Compound **2d**: Following the modified GP3 (reaction for 60 hours), **2d** (41 mg, 64%) was prepared from DBB (31 mg, 0.1 mmol) and **1d** (87 mg, 0.3 mmol). The crude was purified by column chromatography (SiO_2, hexane to hexane/CH_2Cl_2 5:1). A white solid, m. p. 292–293 ºC. R_f = 0.54 (hexane/CH_2Cl_2 5:1). ^1H NMR (500 MHz, $CDCl_3$, ppm): δ = 8.27 (m, 2H), 7.85 (d, J = 8.0 Hz, 2H), 7.76 (m, 2H), 7.62 (d, J = 7.5 Hz, 2H), 7.48 (t, J = 7.5 Hz, 2H), 7.41 (m, 4H), 7.21 (d, J = 8.5 Hz, 4H), 6.70 (d, J = 8.5 Hz, 4H). ^{13}C NMR (125 MHz, $CDCl_3$, plus DEPT, ppm): δ = 147.1 (C_{quat}), 145.9 (C_{quat}), 144.1 (C_{quat}), 141.6 (C_{quat}), 139.0 (C_{quat}), 135.3 (C_{quat}), 131.4 (C_{quat}), 128.9 (CH), 128.1 (CH), 127.7 (CH), 127.2 (q, J = 39 Hz, C_{quat}), 127.1 (CH), 125.4 (CH), 125.1 (q, J = 3.4 Hz, CH), 122.4 (CH), 122.1 (q, J = 258 Hz, CF_3), 121.0 (CH), 119.1 (CH). HRMS (FAB) m/z: $[M + H]^+$ calcd for $C_{42}H_{23}F_6$, 641.1698; found: 641.1698.

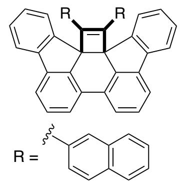

Compound **2g**. Following GP3, **2g** (37 mg, 62%) was prepared from DBB (31 mg, 0.1 mmol) and **1g** (82 mg, 0.3 mmol). The crude was purified by column chromatography (SiO_2, *n*-hexane to hexane/CH_2Cl_2 5:1). A white solid, m. p. 287–288 ºC. R_f = 0.65 (hexane/CH_2Cl_2 3:1). ^1H NMR (500 MHz, $CDCl_3$, ppm): δ = 8.39 (dd, J = 6.5, 1.5 Hz, 2H), 7.86 (d, J = 7.5 Hz, 2H), 7.79–7.77 (m, 2H), 7.63 (dd, J = 7.5, 0.5 Hz, 2H), 7.60 (d, J = 8.5 Hz, 2H), 7.45 (t, J = 7.5 Hz, 2H), 7.42–7.37 (m, 8H), 7.34–7.28 (m, 4H), 7.02 (s, 2H), 6.89 (dd, J = 8.5, 1.5 Hz, 2H). ^{13}C NMR (125 MHz, $CDCl_3$, plus DEPT, ppm): δ = 147.3 (C_{quat}), 147.0 (C_{quat}), 145.3 (C_{quat}), 142.1 (C_{quat}), 139.3 (C_{quat}), 133.1 (C_{quat}), 132.8 (C_{quat}), 131.7 (C_{quat}), 130.1 (C_{quat}), 128.8 (CH), 128.4 (CH), 127.9 (CH), 127.6 (CH), 127.52 (CH), 127.47 (CH), 126.3 (CH), 126.1 (CH), 125.9 (CH), 125.8 (CH), 125.4 (CH), 122.4 (CH), 120.9 (CH), 119.0 (CH), 66.5 (C_{quat}). HRMS (EI) m/z: $[M]^+$ calcd for $C_{48}H_{28}$, 604.2186; found: 604.2184.

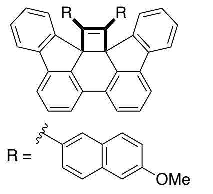

Compound **2h**. Following GP3, **2h** (46 mg, 67%) was prepared from DBB (31 mg, 0.1 mmol) and **1h** (91 mg, 0.3 mmol). The crude was purified by column chromatography (SiO_2, hexane to hexane/CH_2Cl_2 3:1). A white solid, m. p. 292–293 ºC. R_f = 0.42 (hexane/CH_2Cl_2 3:1). ^1H NMR (500 MHz, $CDCl_3$, ppm): δ = 8.35 (dd, J = 7.0, 2.0 Hz, 2H), 7.84 (d, J = 7.5 Hz, 2H), 7.78–7.76 (m, 2H), 7.62 (dd, J = 7.5, 0.5 Hz, 2H), 7.44 (t, J = 7.5 Hz, 2H), 7.42–7.37 (m, 4H), 7.27 (dd, J = 8.5, 4.0 Hz, 4H), 6.96 (dd, J = 9.0, 2.5 Hz, 2H), 6.94 (d, J = 1.0 Hz, 2H), 6.90 (d, J = 2.5 Hz, 2H), 6.87 (dd, J = 8.5, 1.5 Hz, 2H), 3.82 (s, 6H). ^{13}C NMR (125 MHz, $CDCl_3$, plus DEPT, ppm): δ = 157.8 (C_{quat}), 147.0 (C_{quat}), 146.6 (C_{quat}), 145.3 (C_{quat}), 141.9 (C_{quat}), 139.2 (C_{quat}), 133.8 (C_{quat}), 131.6 (C_{quat}), 129.8 (CH), 128.5 (CH), 128.4 (C_{quat}), 128.0 (C_{quat}), 127.7 (CH), 127.4 (CH), 126.1 (CH), 125.9 (CH), 125.6 (CH), 122.2 (CH), 120.7 (CH), 118.8 (CH), 118.5 (CH), 105.5 (CH), 66.2 (C_{quat}), 55.3 (CH_3). HRMS (EI) m/z: $[M]^+$ calcd for $C_{50}H_{32}O_2$, 664.2397; found: 664.2394.

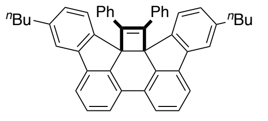

Compound **2i**. Following GP3, **2i** (41 mg, 66%) was prepared from DBB (31 mg, 0.1 mmol) and **1i** (83 mg, 0.3 mmol). The crude was purified by column chromatography (SiO_2, hexane/CH_2Cl_2 8:1). A white solid, m. p. 290–291 ºC. R_f = 0.52 (hexane/CH_2Cl_2 5:1). ^1H NMR (500 MHz, $CDCl_3$, ppm): δ = 8.15 (d, J = 8.0 Hz, 2H), 7.79

(dd, J = 7.5, 0.5 Hz, 2H), 7.56 (dd, J = 7.5, 0.5 Hz, 2H), 7.54 (d, J = 1.5 Hz, 2H), 7.41 (t, J = 7.5 Hz, 2H), 7.19 (dd, J = 7.5, 1.5 Hz. 2H), 7.00 (td, J = 7.5, 1.5 Hz, 2H), 6.92 (t, J = 7.5 Hz, 4H), 6.65 (dd, J = 7.5, 1.5 Hz, 4H), 2.69 (t, J = 7.5 Hz, 4H), 1.64 (quint, J = 7.5 Hz, 4H), 1.35 (sext, J = 7.5 Hz, 4H), 0.93 (t, J = 7.5 Hz, 6H). ^{13}C NMR (125 MHz, $CDCl_3$, plus DEPT, ppm): δ = 147.3 (C_{quat}), 146.7 (C_{quat}), 142.4 (C_{quat}×2), 141.8 (C_{quat}), 139.3 (C_{quat}), 132.8 (C_{quat}), 131.7 (C_{quat}), 128.5 (CH), 128.0 (CH), 127.8 (CH), 127.7 (CH), 127.4 (CH), 125.2 (CH), 122.9 (CH), 120.7 (CH), 118.6 (CH), 66.0 (C_{quat}), 35.9 (CH_2), 33.8 (CH_2), 22.2 (CH_2), 14.0 (CH_3). HRMS (EI) m/z: $[M]^+$ calcd for $C_{48}H_{40}$, 616.3125; found: 616.3121.

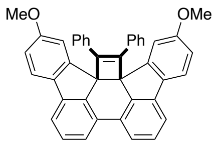

Compound **2j**. Following GP3, **2j** (29 mg, 51%) was prepared from DBB (31 mg, 0.1 mmol) and **1j** (76 mg, 0.3 mmol). The crude was purified by column chromatography (SiO_2, n-hexane to n-hexane/CH_2Cl_2 5:1). A white solid, m. p. 293–294 ºC. R_f = 0.6 (hexane/CH_2Cl_2 3:1). ^{1}H NMR (500 MHz, $CDCl_3$, ppm): δ = 7.82 (d, J = 2.5 Hz, 2H), 7.75 (dd, J = 8.0, 0.5 Hz, 2H), 7.63 (d, J = 8.0 Hz, 2H), 7.49 (dd, J = 7.5, 1.0 Hz, 2H), 7.40 (t, J = 7.5 Hz, 2H), 7.02 (tt, J = 7.5, 1.5 Hz, 2H), 6.96–6.93 (m, 4H), 6.91 (dd, J = 8.5, 2.5 Hz, 2H), 6.69–6.67 (m, 4H), 3.92 (s, 6H). ^{13}C NMR (125 MHz, $CDCl_3$, plus DEPT, ppm): δ = 159.6 (C_{quat}), 147.0 (C_{quat}), 146.9 (C_{quat}), 146.4 (C_{quat}), 139.2 (C_{quat}), 134.9 (C_{quat}), 132.6 (C_{quat}), 131.6 (C_{quat}), 128.6 (CH), 128.1 (CH), 127.7 (CH), 127.2 (CH), 121.5 (CH), 121.3 (CH), 118.1 (CH), 113.0 (CH), 112.1 (CH), 66.2 (C_{quat}), 55.8 (CH_3). HRMS (EI) m/z: $[M]^+$ calcd for $C_{42}H_{28}O_2$, 564.2084; found: 564.2081.

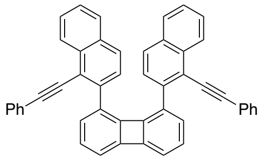

1,8-bis[1-(phenylethynyl)naphth-2-yl]biphenylene (**3k**). Following GP3, **3k** (33 mg, 65%) was prepared from DBB (31 mg, 0.1 mmol) and **1k** (82 mg, 0.3 mmol). The crude was purified by column chromatography (SiO_2, hexane to hexane/CH_2Cl_2 5:1). A white solid, m. p. 290–291 ºC. R_f = 0.55 (hexane/CH_2Cl_2 3:1). ^{1}H NMR (400 MHz, $CDCl_3$, ppm): δ = 7.67 (d, J = 8.2 Hz, 2H), 7.28–7.10 (m, 16H), 7.06 (t, J = 7.0 Hz, 2H), 7.02 (t, J = 8.0 Hz, 2H), 6.96 (d, J = 8.8 Hz, 2H), 6.91 (t, J = 8.0 Hz, 2H), 6.81 (d, J = 6.8 Hz, 2H). ^{13}C NMR (125 MHz, $CDCl_3$, plus DEPT, ppm): δ = 150.9 (C_{quat}), 139.2 (C_{quat}), 132.53 (C_{quat}), 132.47 (C_{quat}), 131.8 (C_{quat}), 130.4 (CH), 130.2 (CH), 128.4 (C_{quat}), 125.8 (CH), 128.0 (CH), 127.7 (CH), 127.2 (CH), 126.9 (CH), 126.0 (CH), 125.9 (CH), 125.8 (CH), 125.7 (CH), 123.9 (C_{quat}), 118.8 (C_{quat}), 115.6 (CH), 97.8 (C_{quat}), 86.6 (C_{quat}). HRMS (FAB) m/z: $[M]^+$ calcd for $C_{48}H_{28}$, 604.2186; found: 604.2190.

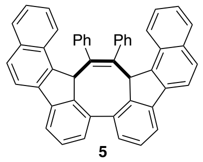

5

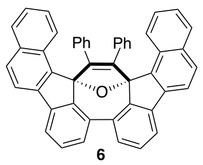

6

Compounds **5** and **6**. A mixture of **3k** (41 mg, 0.067 mmol), $Pd(PPh_3)_4$ (8 mg, 0.0067 mmol), $KHCO_3$ (27 mg, 0.27 mmol), and DMF (0.67 mL) in a thick-walled Pyrex tube was purged with nitrogen for 5 min. The sealed tube was kept in an oil bath at 160 ºC for 16 h. After being cooled to room temperature, water (5 mL) was added. The solution was extracted with ethyl acetate (2×10 mL). The combined extracts were dried over anhydrous $MgSO_4$. The solvent of the filtrate was removed under reduced pressure, and the residue was subjected to chromatography on silica gel. Eluting with hexane/CH_2Cl_2 (5:1) gave **3k** (12 mg, 30%), **5** (3.2 mg, 8%) and **6** (10 mg, 25%). When the reaction was conducted with **3k** (29 mg) for 40 h, a mixture of **3k** (8.1 mg, 20%) and **6** (12.6 mg, 31%) was obtained. Crystals of **5** and **6** were obtained by slow diffusion of methanol into their solutions in DCM. **5**: A white solid, m. p. >300 ºC. $R_f = 0.46$ (hexane/CH_2Cl_2 3:1). ^{1}H NMR (400 MHz, $CDCl_3$, ppm): δ = 8.45 (d, J = 8.0 Hz, 2H), 7.96 (d, J = 8.0 Hz, 2H), 7.84 (d, J = 8.0 Hz, 2H), 7.76–7.65 (m, 8H), 7.54 (t, J = 7.6 Hz, 2H), 7.47 (ddd, J = 8.0, 6.8, 1.2 Hz, 2H), 7.11 (s, 2H), 6.50 (t, J = 7.6 Hz, 2H), 6.37 (t, J = 7.6 Hz, 4H), 6.15 (d, J = 7.6 Hz, 4H). ^{13}C NMR (125 MHz, $CDCl_3$, plus DEPT, ppm): δ = 147.2 (C_{quat}), 142.9 (C_{quat}), 140.6 (C_{quat}), 139.7 (C_{quat}), 139.0 (C_{quat}), 137.9 (C_{quat}), 136.4 (C_{quat}), 133.1 (C_{quat}), 130.6 (CH), 129.2 (CH), 129.1 (CH), 128.22 (CH), 128.19 (CH), 126.5 (CH), 125.9 (CH), 125.5 (CH), 125.2 (CH), 124.2 (CH), 119.4 (CH), 118.3 (CH), 53.1 (CH). Due to overlapping signals, one C_{quat} was not observed. HRMS (FAB) m/z: [M^+] calcd for $C_{48}H_{30}$, 606.2342; found: 606.2346. **6**: A purple solid, m. p. >300 ºC. $R_f = 0.35$ (hexane/CH_2Cl_2 5:1). ^{1}H NMR (500 MHz, $CDCl_3$, ppm): δ = 9.27 (d, J = 8.4 Hz, 2H), 8.08 (d, J = 8.0 Hz, 2H), 7.92 (t, J = 8.0 Hz, 4H), 7.72 (d, J = 8.0 Hz, 2H), 7.67 (t, J = 7.0 Hz, 2H), 7.60 (d, J = 7.5 Hz, 2H), 7.53 (d, J = 9.6 Hz, 2H), 7.50 (d, J = 9.6 Hz, 2H), 6.81 (t, J = 7.0 Hz, 2H), 6.67 (t, J = 7.5 Hz, 4H), 6.32 (d, J = 8.5 Hz, 4H). ^{13}C NMR (125 MHz, $CDCl_3$, plus DEPT, ppm): δ = 149.7 (C_{quat}), 142.4 (C_{quat}), 139.8 (C_{quat}), 139.6 (C_{quat}), 137.3 (C_{quat}), 133.8 (C_{quat}), 132.6 (C_{quat}), 132.4 (C_{quat}), 130.9 (C_{quat}), 130.8 (CH), 129.3 (CH), 129.0 (CH), 128.6 (CH), 127.2 (CH), 126.9 (CH), 126.5 (CH), 125.6 (CH), 125.4 (CH), 125.2 (CH), 119.0 (CH), 118.1 (CH), 100.2 (C_{quat}). HRMS (FAB) m/z: [M^+] calcd for $C_{48}H_{28}O$, 620.2135; found: 620.2142.

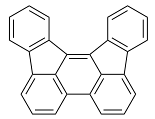

Benz[*e*]indeno[1,2,3-*hi*]acephenanthrylene (**10**): Compound **2a** (8.3 mg, 16.4 μmol) was distributed into five melting point capillary tubes. These were placed in a Büchi B545 melting point apparatus and heated to 330 °C, where the temperature was sustained for 10 minutes. Upon cooling to ambient temperature, the tubes were fractured and immersed in dichloromethane (20 mL). After filtration, the filtrate was concentrated under reduced pressure. The residue was subjected to chromatography on silica gel. Eluting with hexane/CH_2Cl_2 (10:1) gave the title compound (4.6 mg, 85%) as a yellow solid. ^{1}H NMR (400 MHz, $CDCl_3$, ppm): δ = 8.62–8.59 (m, 2H), 8.48 (dd, J = 8.0, 0.8 Hz, 2H), 8.06–8.02 (m, 4H), 7.80 (dd, J = 7.2, 6.8 Hz, 2H), 7.58–7.50 (m, 4H). Spectral data match those reported previously.[S13]

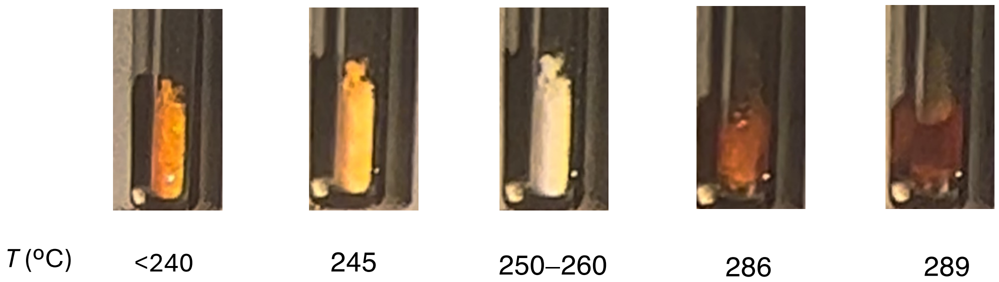

(S13) Mittal, K.; Pham, A. V.; Davis, A. G.; Richardson, A. D.; De Hoe, C.; Dean, R.T.; Baird, V.; McDonald, A. R.; Frantz, D. K. Intramolecular Diels–Alder Reaction of a Biphenyl Group in a Strained meta-Quaterphenylene Acetylene. *J. Org. Chem.* **2024**, *89*, 9620.

C. Thermal rearrangement of **4a** in DMSO-d_6.

353 K								
t (s)	0	900	1200	1500	1800	2100	2400	
(**4a**)$_t$	1	0.9769	0.9687	0.9551	0.9481	0.9385	0.9294	
ln[(**4a**)$_t$/(**4a**)$_0$]	0	-0.0234	-0.0318	-0.0559	-0.0633	-0.0734	-0.0792	

363 K								
t (s)	0	600	900	1200	1500	1800	2100	2400
(**4a**)$_t$	1	0.9569	0.9259	0.9132	0.8929	0.8696	0.8475	0.8264
ln[(**4a**)$_t$/(**4a**)$_0$]	0	-0.04406	-0.07699	-0.0908	-0.11328	-0.13972	-0.16546	-0.19068

373 K									
t (s)	0	300	600	900	1200	1500	1800	2100	2400
(**4a**)$_t$	1	0.9260	0.8772	0.8403	0.7874	0.7407	0.6757	0.6289	0.6024
ln[(**4a**)$_t$/(**4a**)$_0$]	0	-0.07688	-0.13102	-0.174	-0.23902	-0.30016	-0.39201	-0.46378	-0.50683

383 K									
t (s)	0	300	600	900	1200	1500	1800	2100	2400
(**4a**)$_t$	1	0.8929	0.7874	0.6667	0.5618	0.4717	0.3876	0.3135	0.25
ln[(**4a**)$_t$/(**4a**)$_0$]	0	-0.11328	-0.23902	-0.40542	-0.57661	-0.75141	-0.94778	-1.15996	-1.38629

393 K									
t (s)	0	300	600	900	1200	1500	1800	2100	2400
(**4a**)$_t$	1	0.6545	0.3679	0.2401	0.1491	0.0972	0.0751	0.0525	0.0313
ln[(**4a**)$_t$/(**4a**)$_0$]	0	-0.4239	-0.9999	-1.4267	-1.9031	-2.3310	-2.5889	-2.9469	-3.4641

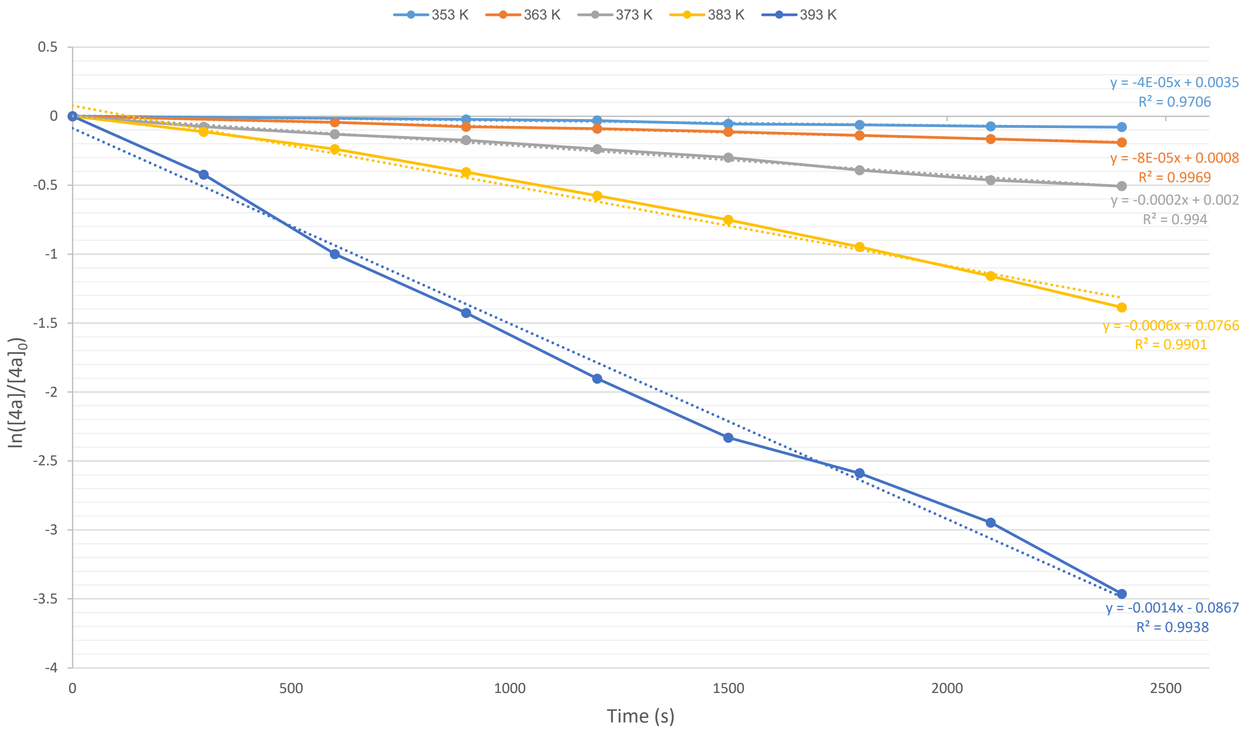

353 K
363 K
373 K
383 K
393 K
y = -4E-05x + 0.0035
R² = 0.9706
y = -8E-05x + 0.0008
R² = 0.9969
y = -0.0002x + 0.002
R² = 0.994
y = -0.0006x + 0.0766
R² = 0.9901
y = -0.0014x - 0.0867
R² = 0.9938
ln([4a]/[4a]0)
Time (s)

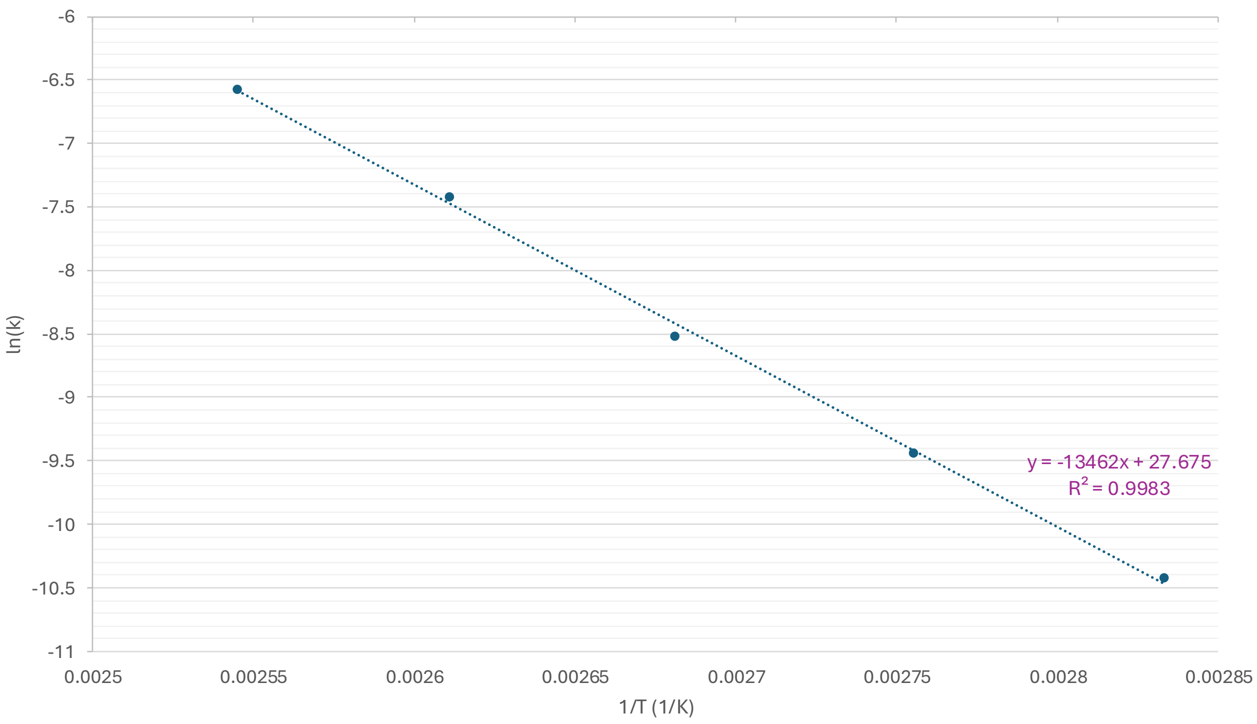

y = -13462x + 27.675
R² = 0.9983
ln(k)
1/T (1/K)

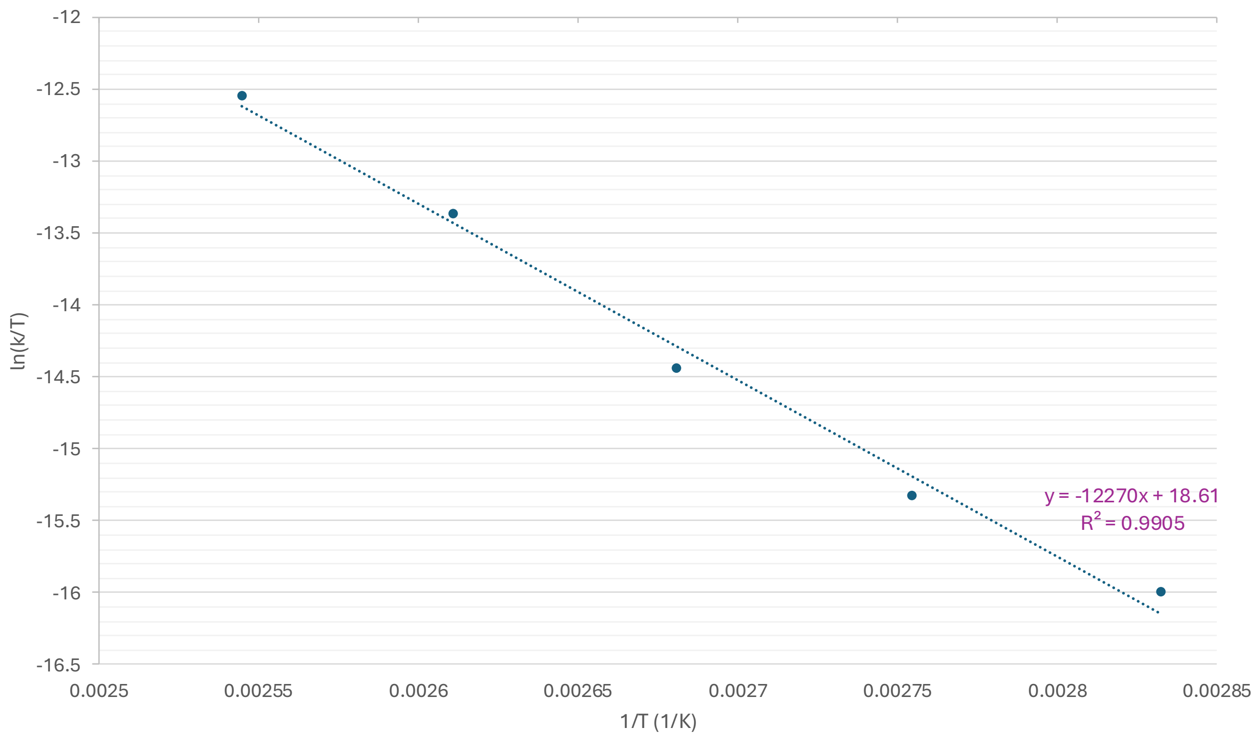

T (K)	k	ln$k$	1/T
353	0.00004	-10.4144	0.002833
363	0.00008	-9.43348	0.002755
373	0.0002	-8.51719	0.002681
383	0.0006	-7.41858	0.002611
393	0.0014	-6.57128	0.002545

E_a	26.75 kcal•mol^{-1}
$\Delta H^{\ddagger}$	24.38 kcal•mol^{-1}
ΔS	−10.23 cal•mol^{-1}•K^{-1}
$\Delta G^{\ddagger}$ (298K)	27.43 kcal•mol^{-1}

Figure S1. Kinetic data for the **4a**/**2a** TR.

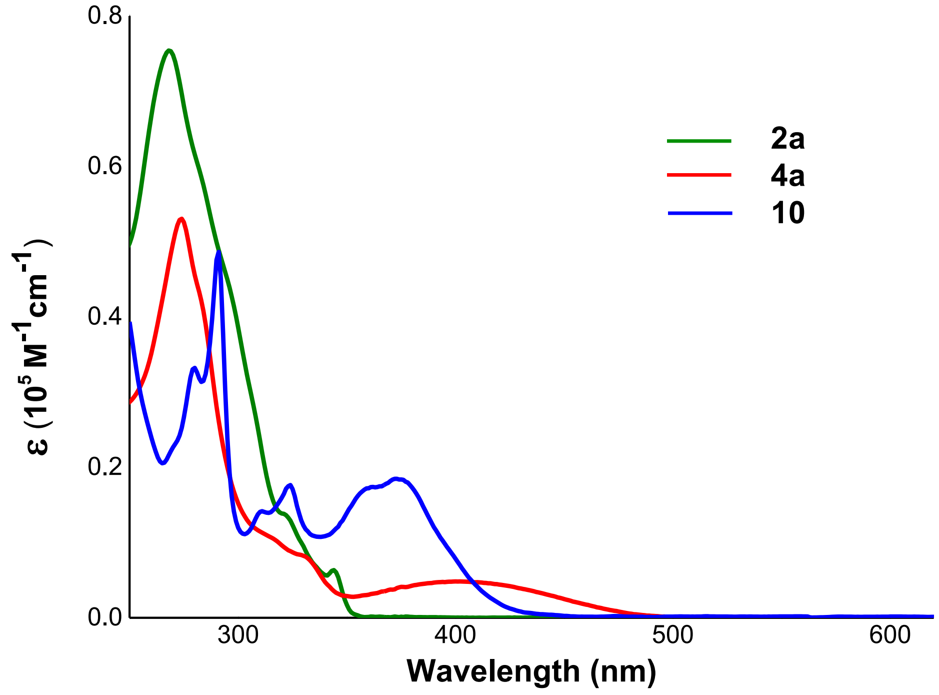

Figure S2. Absorption spectra of studied compounds (10 μM in dichloromethane).

D. Structural Analysis

Table S1. Crystallographic data and structure refinement details

	**2a**	**4a**	**5**	**6**
CCDC	2415662	2415663	2415666	2415667
formula	$C_{40}H_{24}$	$C_{40}H_{24}$	$C_{48}H_{30}$	$C_{48}H_{28}O$
formula weight	504.59	504.59	606.72	620.70
temperature (K)	100(2)	100(2)	100.00(10)	99.99(14)
wavelength (Å)	0.71073	0.71073	1.54184	1.54184
crystal system	Triclinic	Orthorhombic	triclinic	triclinic
space group	*P*-1	*Pbcn*	*P*-1	*P*-1
a (Å)	9.5932(5)	16.2727(7)	12.8602(3)	12.7649(2)
b (Å)	11.1792(6)	11.2704(5)	14.0229(3)	13.1005(2)
c (Å)	13.4230(7)	14.3338(6)	19.8320(3)	20.7670(3)
α (deg)	104.9980(10)	90	88.494(2)	89.1458(13)
β (deg)	108.7670(10)	90	71.710(2)	73.3508(15)
γ (deg)	97.1010(10)	90	67.131(2)	67.9212(16)
Volume (Å^3)	1282.45(12)	2628.8(2)	3110.08(12)	3066.32(10)
Z	2	4	4	4
d_{calc} (g/cm^3)	1.307	1.275	1.296	1.345
F(000)	528	1056	1272.0	1296.0
crystal size (mm)	0.764×0.645×0.328	0.196×0.134×0.058	0.21×0.15×0.07	0.18×0.17×0.15
θ range (deg)	2.995 to 25.442	2.618 to 28.320	3.878 to 76.928	3.661 to 77.106
reflns collected	29412	23031	40653	38403
indep reflns/R_{int}	4659/0.0199	3264/0.0502	12447/0.0258	1237/0.0236
parameters	361	181	866	884
GOF on F^2	1.032	1.091	1.075	1.037
R_1, wR_2 [I>2σ(I)]	0.0589, 0.1445	0.0502, 0.1066	0.0491, 0.1270	0.0391, 0.0977
R_1, wR_2 (all data)	0.0592, 0.1446	0.0586, 0.1106	0.0521, 0.1290	0.0428, 0.1002

Table S2. Structural parameters for **4a**, **4k** and **4l**

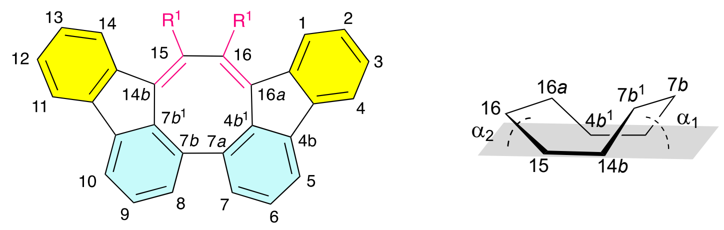

	4a		4k	4l
	X-ray	DFT	DFT	DFT
Bond length (Å)				
C15–C16	1.495(2)	1.481	1.492	**1.435**
C16–C16*a*	1.352(2)	1.358	1.359	1.353
C16*a*–C4*b*^{1}	1.483(2)	1.471	1.471	1.472
C4*b*^{1}–C7*a*	1.391(2)	1.390	1.385	1.396
C7*a*–C7*b*	1.492(2)	1.484	1.481	1.492
C14*a*–C14*b*	1.496(2)	1.487	1.490	1.472
C10*a*–C10*b*	1.467(2)	1.460	1.462	1.460
Torsion angle (deg)				
C14*b*-C15-C16-C16*a*	51.3	52.3	**55.8**	**25.9**
C15-C16-C16*a*-C4*b*^{1}	3.7	3.1	1.4	6.4
C16-C16*a*-C4*b*^{1}-C7*a*	41.7	42.9	**51.8**	**30.9**
C16*a*-C4*b*^{1}-C7*a*-C7*b*	9.4	10.5	6.4	16.7
C15-C16-C7a-C7*b*	55.9	58.3	**64.8**	**41.1**
C16-C16*a*-C7*b*-C7*b*^{1}	3.2	3.2	2.1	2.9
C16*a*-C4*b*^{1}-C7*b*^{1}-C14*b*	50.1	52.3	**61.8**	**36.0**
C4*b*^{1}-C7*a*-C14*b*-C15	3.2	3.4	2.1	2.5
C7-C7a-C7*b*-C8	41.7	44.8	48.1	41.1
C14-C14a-C14*b*-C15	29.7.2	30.2	**43.8**	22.4
Bond angle (deg)				
C15-C16-C16*a*	130.0(1)	129.9	128.6	134.1
C16-C16*a*-C4*b*^{1}	128.3(1)	127.5	124.5	131.3
C16*a*-C4*b*^{1}-C7*a*	131.4(1)	131.1	130.3	132.9
C4*b*^{1}-C7*a*-C7*b*	126.1(1)	125.2	123.4	127.5
C(Ar)-C15-C16	110.9(1)	112.5	110.1	—
α_1, α_2 (deg)	33.3, 38.2	34.1, 40.2	38.5, 45.0	33.0, 20.3

Table S3. Selected structural parameters for **2a** and **2l**.

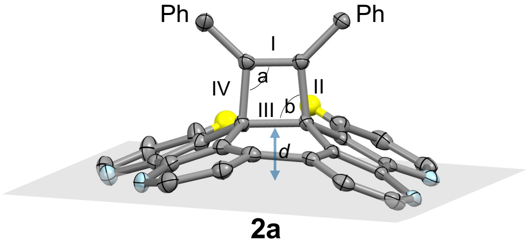

2a

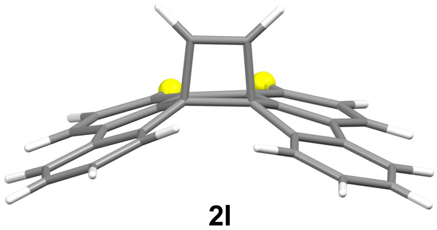

2l

	**2a**		**2l**
	X-ray	DFT	DFT
Bond length (Å)			
I	1.343(3)	1.345	1.328
II	1.541(2)	1.534	1.526
III	1.608(3)	1.588	1.601
IV	1.541(2)	1.539	1.523
Bond angle (deg)			
a	95	95	95
b	85	85	85
H···H (Å)	2.138	2.060	1.984
d (Å)	1.454	1.342	1.316

E. Computational Details

Density functional theory (DFT) calculations were performed using the Gaussian 16 software package. Geometry optimizations and subsequent vibrational frequency analyses were carried out with the PW6B95 functional and the 6-31G** basis set. To obtain more accurate electronic energies, single-point energy calculations were conducted using the same functional but with the larger 6-311++G** basis set. Van der Waals interactions were described by Grimme's D3 empirical dispersion correction.

$$G = E_{\mathrm{elec}} + \mathrm{ZPE} + 4\mathrm{RT} + H_{\mathrm{vib}} - \mathrm{T}(S_{\mathrm{trans}} + S_{\mathrm{rot}} + S_{\mathrm{vib}})$$

E_{elec}, ZPE, and H_{vib} are the electronic energy, zero-point energy correction, and vibrational enthalpy, respectively. T = 298 K is used for all calculations. The term 4RT accounts for translational enthalpy, rotational enthalpy, and PV work.

The Gibbs free energy of calculated species:

Species	E_{elec}	ZPE + H_{vib} + 4RT − TS	G_{total}
**4a**	−1541.1931	0.454749	−1540.7383
TS-**a**	−1541.1470	0.452820	−1540.6942
**2a**	−1541.1968	0.455108	−1540.7417
**4l**	−1078.3519	0.304498	−1078.0474
**2l**	−1078.3219	0.303485	−1078.0184

Coordinates of optimized structures

4a

C	1.446902000	-0.963813000	-0.141533000
C	-0.158761000	1.800516000	-1.612259000
C	2.407229000	0.127254000	-0.363881000
C	1.172346000	1.660873000	-1.988294000
C	2.290193000	1.315994000	-1.075447000
C	-0.847356000	1.468792000	-0.355900000
C	2.280068000	-2.159029000	0.157024000
C	3.663361000	-0.279531000	0.125141000
C	3.584332000	-1.702094000	0.443801000
C	3.398080000	2.176580000	-1.071181000
C	4.592907000	1.834112000	-0.452688000
H	5.420311000	2.530712000	-0.468973000
C	4.752316000	0.575444000	0.114807000
H	5.710724000	0.261610000	0.506951000
H	3.315359000	3.121631000	-1.591184000
C	2.004525000	-3.520101000	0.246504000
C	3.012195000	-4.390272000	0.653431000
C	4.279921000	-3.923641000	0.981682000
C	4.575708000	-2.570146000	0.871346000
H	5.567882000	-2.202849000	1.100033000
H	1.038797000	-3.920911000	-0.017733000
H	2.800719000	-5.449724000	0.708438000
H	5.043755000	-4.618456000	1.304274000
C	-2.048720000	2.344752000	-0.329419000
C	-2.222459000	2.861078000	-1.631583000
C	-0.698521000	2.904156000	-3.714875000
C	-1.050117000	2.519025000	-2.432024000
C	1.513144000	2.047404000	-3.293192000
C	0.588013000	2.618794000	-4.156253000
H	0.892484000	2.896416000	-5.156423000
H	-1.394562000	3.441528000	-4.345435000
H	2.536892000	1.914404000	-3.616545000
C	-2.976501000	2.667127000	0.656357000
C	-4.072106000	3.458759000	0.322996000
C	-4.260451000	3.921110000	-0.974476000
C	-3.327557000	3.628383000	-1.962162000
H	-3.454072000	4.006383000	-2.968507000

H	-5.124868000	4.526986000	-1.211084000
H	-4.784788000	3.720511000	1.093556000
H	-2.853983000	2.342286000	1.677479000
C	-0.696964000	0.350852000	0.399507000
C	0.127001000	-0.855881000	0.156123000
C	-0.626119000	-2.121616000	0.408511000
C	-1.265199000	-2.743869000	-0.660831000
C	-0.683856000	-2.708351000	1.672728000
C	-1.938882000	-3.945624000	-0.474389000
H	-1.218715000	-2.287803000	-1.641104000
C	-1.354682000	-3.906807000	1.858070000
H	-0.193061000	-2.221722000	2.504489000
C	-1.980639000	-4.530591000	0.784290000
H	-2.426088000	-4.424894000	-1.313054000
H	-1.387860000	-4.356428000	2.841353000
H	-2.501547000	-5.467508000	0.929864000
C	-1.571583000	0.212403000	1.603162000
C	-2.844075000	-0.354006000	1.528735000
C	-1.109324000	0.689615000	2.826954000
C	-3.646258000	-0.424452000	2.656668000
H	-3.200776000	-0.730072000	0.579621000
C	-1.914105000	0.618191000	3.958589000
H	-0.122286000	1.129749000	2.883118000
C	-3.184903000	0.065148000	3.873760000
H	-4.633932000	-0.859883000	2.586189000
H	-1.548591000	0.998610000	4.903119000
H	-3.813030000	0.012423000	4.752982000

TS-**a**

C	0.213141000	0.576246000	2.105303000
C	-0.735703000	3.728743000	1.337925000
C	1.345300000	1.500606000	2.146565000
C	0.627880000	3.904547000	1.651520000
C	1.502368000	2.903242000	2.285873000
C	-1.698612000	2.804425000	1.819498000
C	0.753251000	-0.726652000	1.725311000
C	2.523905000	0.694502000	2.132418000
C	2.156402000	-0.664158000	1.753850000
C	2.750436000	3.345507000	2.772861000
C	3.843429000	2.513340000	2.916506000

H	4.775500000	2.911230000	3.293761000
C	3.755390000	1.181293000	2.515704000
H	4.619646000	0.530864000	2.544571000
H	2.862445000	4.394039000	3.009616000
C	0.111816000	-1.908911000	1.369339000
C	0.885903000	-3.015240000	1.043854000
C	2.278212000	-2.952177000	1.087739000
C	2.926069000	-1.773568000	1.446592000
H	4.007202000	-1.726879000	1.473475000
H	-0.967790000	-1.965969000	1.349385000
H	0.402559000	-3.938484000	0.754252000
H	2.860905000	-3.827797000	0.834722000
C	-2.897498000	2.967258000	1.030951000
C	-2.713692000	4.052836000	0.142909000
C	-0.719548000	5.639766000	-0.192544000
C	-1.367742000	4.560496000	0.358218000
C	1.249476000	5.027815000	1.070722000
C	0.597896000	5.885966000	0.200912000
H	1.140885000	6.720210000	-0.221989000
H	-1.210200000	6.276695000	-0.916903000
H	2.304533000	5.184783000	1.234829000
C	-4.109040000	2.278182000	1.057369000
C	-5.117269000	2.666435000	0.185534000
C	-4.930404000	3.735243000	-0.691723000
C	-3.727772000	4.439583000	-0.714367000
H	-3.594617000	5.272277000	-1.393236000
H	-5.730669000	4.022599000	-1.360274000
H	-6.060469000	2.137004000	0.187621000
H	-4.262009000	1.464356000	1.753858000
C	-1.659058000	1.922417000	2.994472000
C	-0.978847000	0.713541000	2.872711000
C	-1.441057000	-0.478282000	3.637576000
C	-2.788477000	-0.840221000	3.626213000
C	-0.548842000	-1.243191000	4.394497000
C	-3.234606000	-1.938782000	4.346171000
H	-3.484337000	-0.251756000	3.043972000
C	-0.994834000	-2.334835000	5.122074000
H	0.497636000	-0.968945000	4.411325000
C	-2.339284000	-2.688464000	5.098025000
H	-4.281750000	-2.209163000	4.320026000

H	-0.292303000	-2.911581000	5.708648000
H	-2.686171000	-3.543960000	5.661960000
C	-2.417101000	2.329834000	4.181415000
C	-2.111995000	1.797621000	5.448747000
C	-3.387505000	3.345704000	4.129012000
C	-2.779222000	2.216095000	6.584388000
H	-1.327107000	1.063686000	5.541953000
C	-4.055760000	3.760244000	5.269962000
H	-3.624612000	3.815511000	3.187939000
C	-3.766519000	3.192759000	6.503308000
H	-2.517568000	1.786031000	7.541980000
H	-4.804721000	4.536993000	5.191606000
H	-4.289202000	3.518045000	7.392722000

2a

C	0.653747000	-0.703472000	-0.471632000
C	0.367481000	1.855607000	-0.547204000
C	2.079286000	-0.274470000	-0.278433000
C	1.585678000	2.099224000	0.079111000
C	2.525927000	0.968861000	0.162094000
C	-0.305139000	0.531227000	-0.749275000
C	0.805967000	-1.988775000	-1.246136000
C	2.959516000	-1.276849000	-0.688151000
C	2.166290000	-2.338847000	-1.311554000
C	3.903034000	1.102408000	0.375619000
C	4.776766000	0.059981000	0.086995000
H	5.835553000	0.197800000	0.261082000
C	4.325481000	-1.125153000	-0.488584000
H	5.026129000	-1.894807000	-0.784460000
H	4.306722000	2.044090000	0.722068000
C	-0.159712000	-2.881501000	-1.676350000
C	0.237317000	-4.082505000	-2.256794000
C	1.586443000	-4.406323000	-2.364451000
C	2.560469000	-3.544508000	-1.875699000
H	3.607643000	-3.813734000	-1.926292000
H	-1.210156000	-2.666166000	-1.536699000
H	-0.510766000	-4.776923000	-2.614763000
H	1.877486000	-5.346562000	-2.813234000
C	-1.285199000	0.835022000	-1.853615000
C	-1.429944000	2.236262000	-1.935703000

C	-0.065119000	4.199361000	-0.876718000
C	-0.406852000	2.872519000	-1.101970000
C	1.914929000	3.440425000	0.305966000
C	1.081266000	4.464492000	-0.131178000
H	1.362308000	5.490566000	0.064767000
H	-0.656274000	5.010144000	-1.281588000
H	2.846300000	3.694522000	0.793947000
C	-2.115754000	0.009563000	-2.589363000
C	-3.085698000	0.581265000	-3.410703000
C	-3.240599000	1.962008000	-3.474891000
C	-2.411386000	2.799816000	-2.737744000
H	-2.528148000	3.874454000	-2.791639000
H	-4.006403000	2.386883000	-4.109908000
H	-3.726762000	-0.058045000	-4.002716000
H	-2.013794000	-1.063502000	-2.544194000
C	-0.981214000	0.146944000	0.579158000
C	-0.146535000	-0.878289000	0.825454000
C	-0.022717000	-1.870488000	1.883658000
C	-1.127162000	-2.193673000	2.682610000
C	1.182088000	-2.545208000	2.102386000
C	-1.020927000	-3.147973000	3.680260000
H	-2.071728000	-1.697905000	2.505893000
C	1.283412000	-3.500608000	3.102583000
H	2.044235000	-2.309620000	1.496557000
C	0.185715000	-3.804812000	3.896423000
H	-1.884651000	-3.386292000	4.286748000
H	2.224996000	-4.009356000	3.260850000
H	0.266600000	-4.551700000	4.674824000
C	-2.010000000	0.855316000	1.336698000
C	-1.789091000	1.132387000	2.691158000
C	-3.197910000	1.296435000	0.744348000
C	-2.729986000	1.826031000	3.433963000
H	-0.866381000	0.799685000	3.148154000
C	-4.136676000	1.992550000	1.493014000
H	-3.395574000	1.081003000	-0.295944000
C	-3.907397000	2.260942000	2.835712000
H	-2.541744000	2.034659000	4.478706000
H	-5.052888000	2.324144000	1.022828000
H	-4.641392000	2.806032000	3.414134000

4l

C	0.352310000	-1.802039000	0.329639000
C	-0.655751000	1.639440000	-0.039762000
C	1.513561000	-0.927163000	0.101194000
C	0.668661000	1.505737000	0.382122000
C	1.650072000	0.457029000	-0.021858000
C	-1.593793000	0.718611000	-0.702203000
C	0.902618000	-3.127630000	0.658017000
C	2.658502000	-1.753338000	0.030252000
C	2.284619000	-3.111193000	0.415965000
C	2.907232000	0.934132000	-0.422912000
C	4.005870000	0.102176000	-0.594783000
H	4.952105000	0.526233000	-0.902428000
C	3.900662000	-1.253250000	-0.323326000
H	4.762589000	-1.904046000	-0.387102000
H	3.021592000	1.997804000	-0.579990000
C	0.277907000	-4.294232000	1.073833000
C	1.045778000	-5.440541000	1.245842000
C	2.415803000	-5.424919000	0.992842000
C	3.046317000	-4.258757000	0.572891000
H	4.111096000	-4.250057000	0.378861000
H	-0.784419000	-4.315606000	1.280966000
H	0.575140000	-6.353799000	1.584024000
H	2.994947000	-6.328111000	1.130312000
C	-2.728438000	1.545590000	-1.145904000
C	-2.594250000	2.825452000	-0.586824000
C	-0.768423000	3.911903000	0.859956000
C	-1.325380000	2.872259000	0.134117000
C	1.194637000	2.554559000	1.151747000
C	0.490482000	3.723682000	1.408949000
H	0.947948000	4.500356000	2.006717000
H	-1.303180000	4.842673000	0.994965000
H	2.199033000	2.448446000	1.537810000
C	-3.838332000	1.230677000	-1.916362000
C	-4.808866000	2.204750000	-2.122476000
C	-4.679294000	3.470921000	-1.555719000
C	-3.570144000	3.790637000	-0.780195000
H	-3.472249000	4.775716000	-0.342614000
H	-5.447062000	4.213626000	-1.725917000
H	-5.672453000	1.978279000	-2.733101000

H	-3.945508000	0.252523000	-2.367664000
C	-1.714650000	-0.627609000	-0.641289000
C	-0.946318000	-1.672617000	-0.027384000
H	-2.641501000	-1.002604000	-1.062215000
H	-1.522610000	-2.585090000	0.082786000

2l

C	0.064793000	-0.873377000	0.585993000
C	-0.226530000	1.679224000	0.333189000
C	1.488840000	-0.435352000	0.771033000
C	1.042150000	1.970583000	0.827838000
C	1.948866000	0.840322000	1.087181000
C	-0.936114000	0.359986000	0.389080000
C	0.224824000	-2.183253000	-0.149902000
C	2.355643000	-1.503141000	0.541298000
C	1.572074000	-2.586488000	-0.055373000
C	3.316655000	0.963451000	1.361001000
C	4.169689000	-0.129173000	1.255663000
H	5.220890000	0.001118000	1.475461000
C	3.711748000	-1.364359000	0.803616000
H	4.402152000	-2.182227000	0.644481000
H	3.731532000	1.928134000	1.619778000
C	-0.708308000	-3.036111000	-0.710964000
C	-0.292939000	-4.269801000	-1.206956000
C	1.036883000	-4.664079000	-1.112686000
C	1.978737000	-3.825365000	-0.529183000
H	3.014686000	-4.129932000	-0.453851000
H	-1.746983000	-2.754741000	-0.783984000
H	-1.016210000	-4.928689000	-1.668389000
H	1.339916000	-5.628566000	-1.497523000
C	-2.076973000	0.563102000	-0.578629000
C	-2.137886000	1.922934000	-0.937382000
C	-0.581031000	3.946954000	-0.400883000
C	-0.996388000	2.623178000	-0.345680000
C	1.441399000	3.311676000	0.779847000
C	0.627079000	4.281188000	0.205280000
H	0.967760000	5.307745000	0.183085000
H	-1.166663000	4.699633000	-0.912068000
H	2.414986000	3.600012000	1.152083000
C	-3.092223000	-0.295659000	-0.960579000

C	-4.120250000	0.182289000	-1.768787000
C	-4.156623000	1.518121000	-2.155272000
C	-3.173420000	2.401923000	-1.728086000
H	-3.217029000	3.447675000	-2.003907000
H	-4.964829000	1.874032000	-2.780017000
H	-4.903652000	-0.490668000	-2.090494000
H	-3.107781000	-1.320616000	-0.620094000
C	-1.410208000	0.025055000	1.797480000
C	-0.601213000	-1.016156000	1.951851000
H	-2.131808000	0.536476000	2.418502000
H	-0.382975000	-1.709375000	2.751882000

4k

C	1.64040000	-0.86970000	0.42660000
C	1.28640000	2.49830000	-0.17310000
C	2.33620000	-0.50740000	-0.81770000
C	2.51900000	1.99510000	-0.55580000
C	2.68550000	0.73590000	-1.31830000
C	-0.05780000	1.91320000	-0.29380000
C	2.16840000	-2.22040000	0.76800000
C	2.83700000	-1.68510000	-1.39850000
C	2.69170000	-2.75470000	-0.41210000
C	3.38270000	0.76410000	-2.53560000
C	3.74640000	-0.40490000	-3.18990000
H	4.27370000	-0.34430000	-4.13250000
C	3.50920000	-1.64840000	-2.60810000
H	3.88530000	-2.55300000	-3.06790000
H	3.65600000	1.72420000	-2.95310000
C	2.22730000	-2.97920000	1.96530000
C	2.61680000	-4.35080000	1.86280000
C	3.00300000	-4.88970000	0.61240000
C	3.08510000	-4.09830000	-0.50210000
H	3.44800000	-4.49730000	-1.44020000
H	3.28580000	-5.93370000	0.56670000
C	-0.96630000	3.08870000	-0.41190000
C	-0.26970000	4.18370000	0.10620000
C	2.25900000	4.63350000	0.44320000
C	1.14690000	3.84020000	0.21990000
C	3.63950000	2.81230000	-0.34310000

C	3.51570000	4.08880000	0.18810000
H	4.40320000	4.68630000	0.34800000
H	2.15820000	5.66370000	0.75860000
H	4.61470000	2.43580000	-0.62240000
C	-2.28320000	3.27030000	-0.90800000
C	-2.92450000	4.52070000	-0.64490000
C	-2.22220000	5.55600000	0.01690000
C	-0.90140000	5.41250000	0.34800000
H	-0.34810000	6.23100000	0.78950000
H	-2.73830000	6.48840000	0.20730000
C	-0.39700000	0.66870000	0.13380000
C	0.45500000	-0.34020000	0.82770000
C	-0.26410000	-0.89010000	2.01560000
C	-0.41420000	-0.08360000	3.14280000
C	-0.82640000	-2.16410000	2.01010000
C	-1.07940000	-0.56100000	4.26380000
H	0.00490000	0.91510000	3.13730000
C	-1.50010000	-2.63650000	3.12560000
H	-0.72360000	-2.78200000	1.12830000
C	-1.62080000	-1.84040000	4.25780000
H	-1.17830000	0.06710000	5.13930000
H	-1.92730000	-3.63010000	3.11300000
H	-2.14180000	-2.21290000	5.12960000
C	-1.81820000	0.21040000	0.10630000
C	-2.16800000	-0.82420000	-0.76050000
C	-2.78710000	0.75030000	0.94800000
C	-3.47720000	-1.28250000	-0.81470000
H	-1.40810000	-1.25760000	-1.39900000
C	-4.09200000	0.28490000	0.90150000
H	-2.51050000	1.54070000	1.63250000
C	-4.44250000	-0.72580000	0.01470000
H	-3.74190000	-2.07620000	-1.50080000
H	-4.83830000	0.71560000	1.55530000
H	-5.46270000	-1.08360000	-0.02390000
C	-2.95850000	2.33650000	-1.72900000
H	-2.45070000	1.42770000	-2.00950000
C	-4.22880000	2.57590000	-2.17790000
H	-4.72200000	1.84190000	-2.80090000
C	-4.89450000	3.77100000	-1.84050000
C	-4.24730000	4.72430000	-1.10250000

H	-5.90390000	3.94340000	-2.18920000
H	-4.73110000	5.66590000	-0.87420000
C	2.65990000	-5.14240000	3.03420000
C	2.39320000	-4.60170000	4.26270000
H	2.43620000	-5.21800000	5.15060000
C	2.09700000	-3.22860000	4.37370000
H	1.93150000	-2.79050000	5.34870000
C	2.02250000	-2.44040000	3.25760000
H	2.93180000	-6.18630000	2.93970000
H	1.81300000	-1.38720000	3.35510000

F. NMR spectra

^{1}H NMR spectrum of **S1a** in $CDCl_3$ (400 MHz)

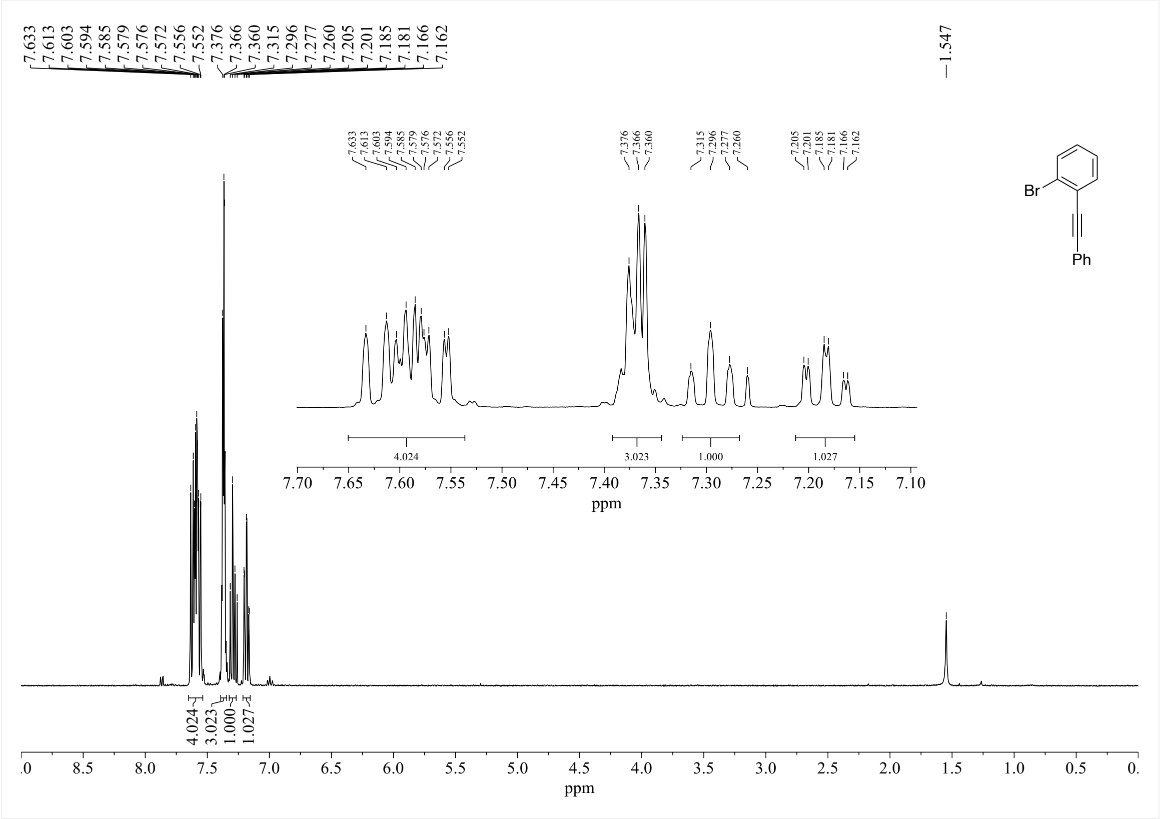

^{1}H NMR spectrum of **S1b** in $CDCl_3$ (400 MHz)

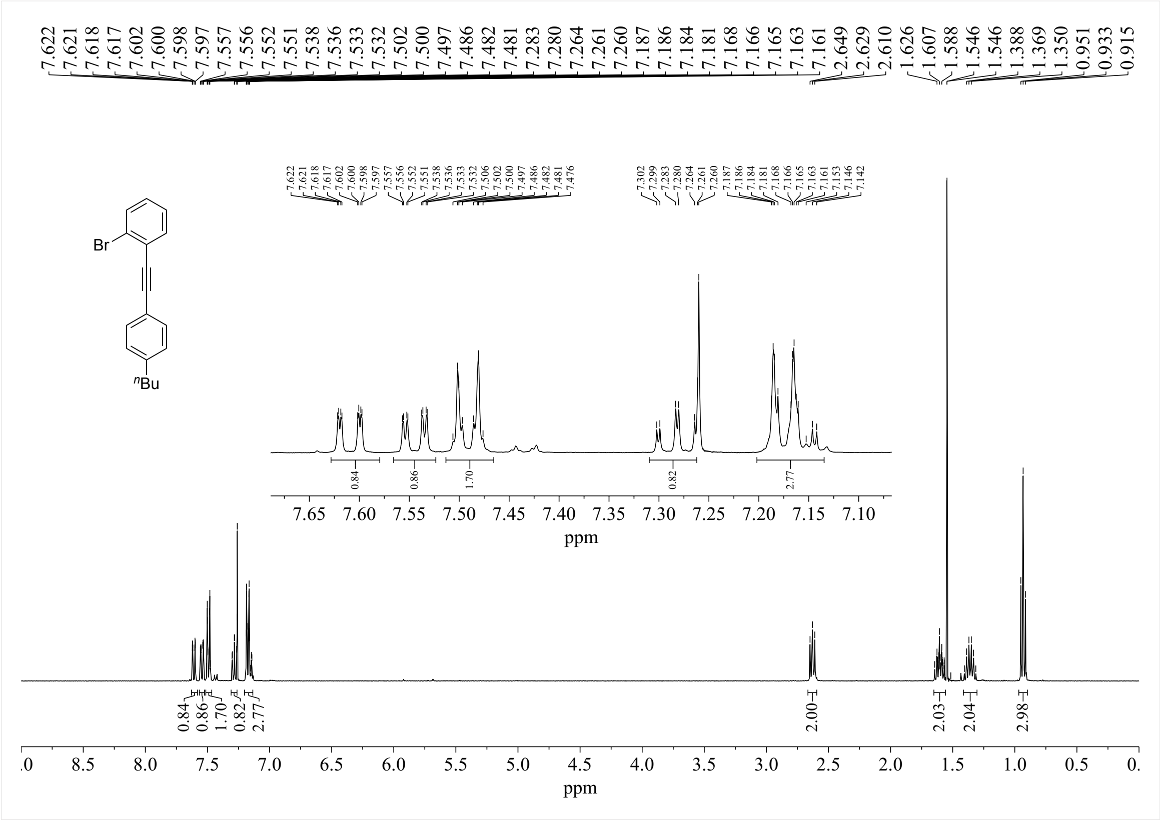

^{1}H NMR spectrum of **S1c** in $CDCl_3$ (400 MHz)

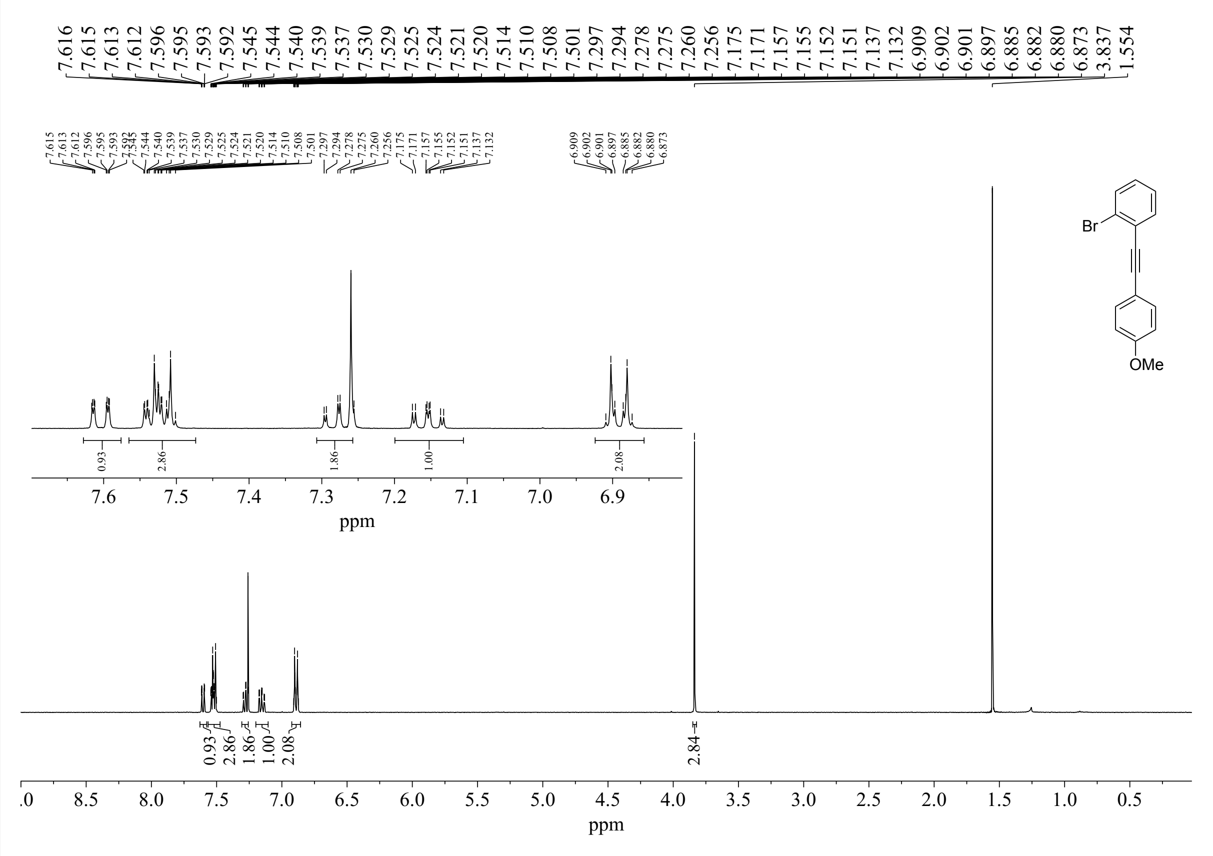

^{1}H NMR spectrum of **S1d** in $CDCl_3$ (400 MHz)

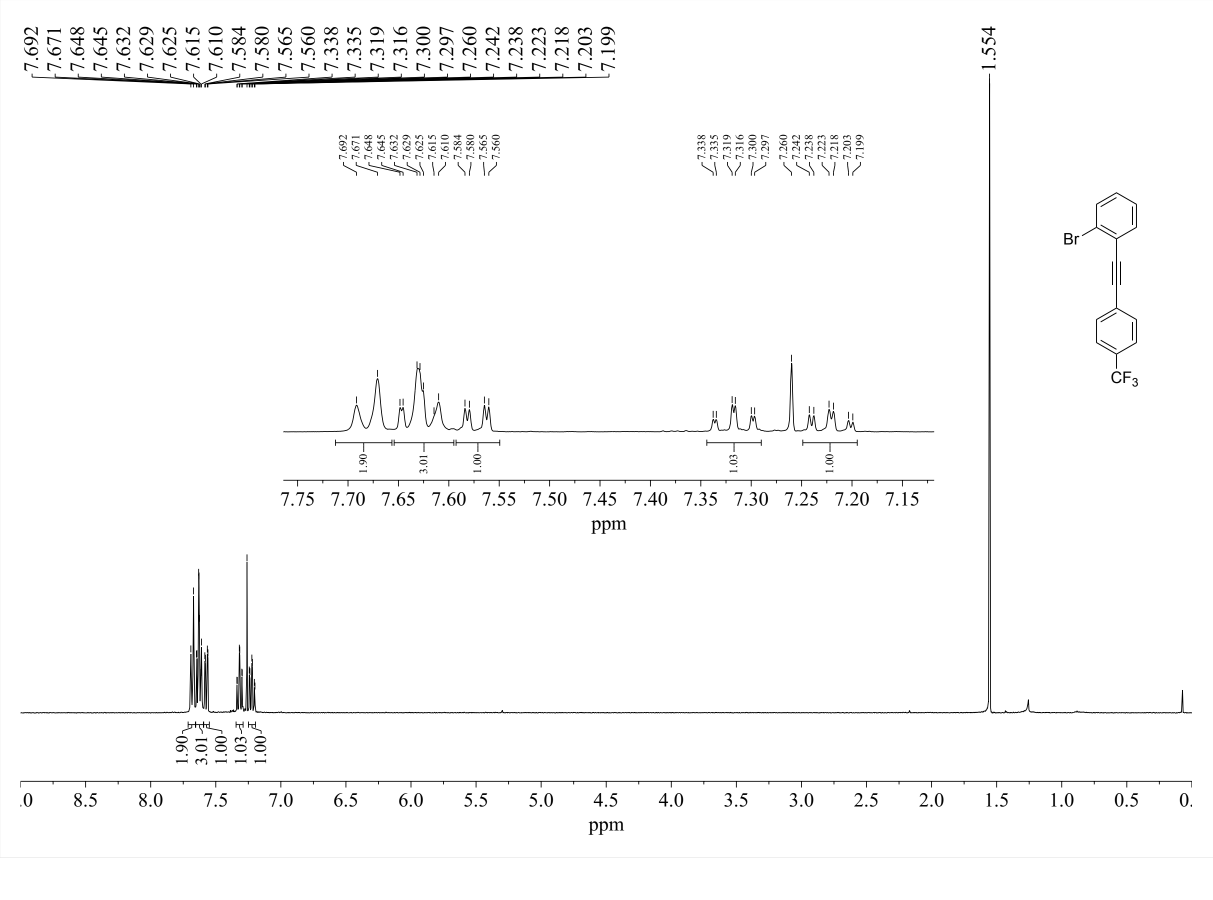

^{1}H NMR spectrum of **S1e** in $CDCl_3$ (400 MHz)

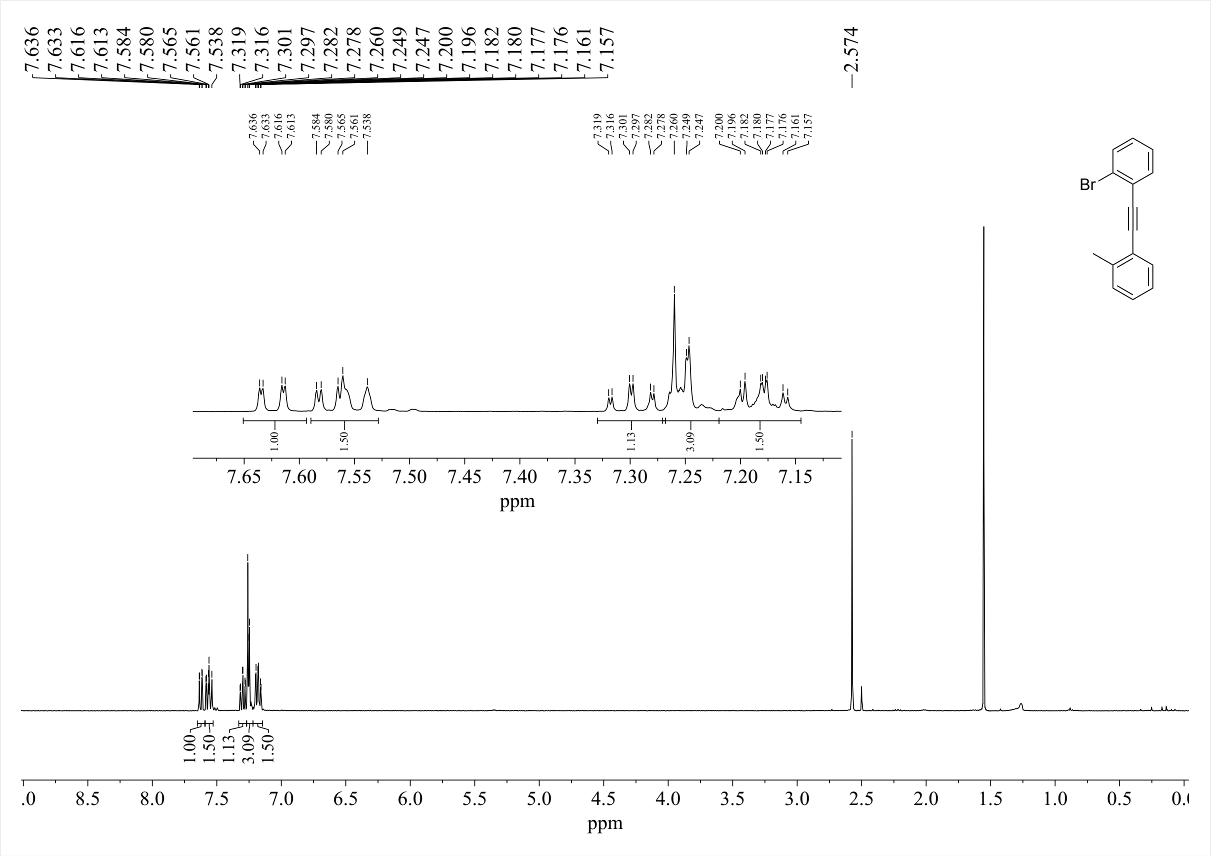

^{1}H NMR spectrum of **S1f** in $CDCl_3$ (400 MHz)

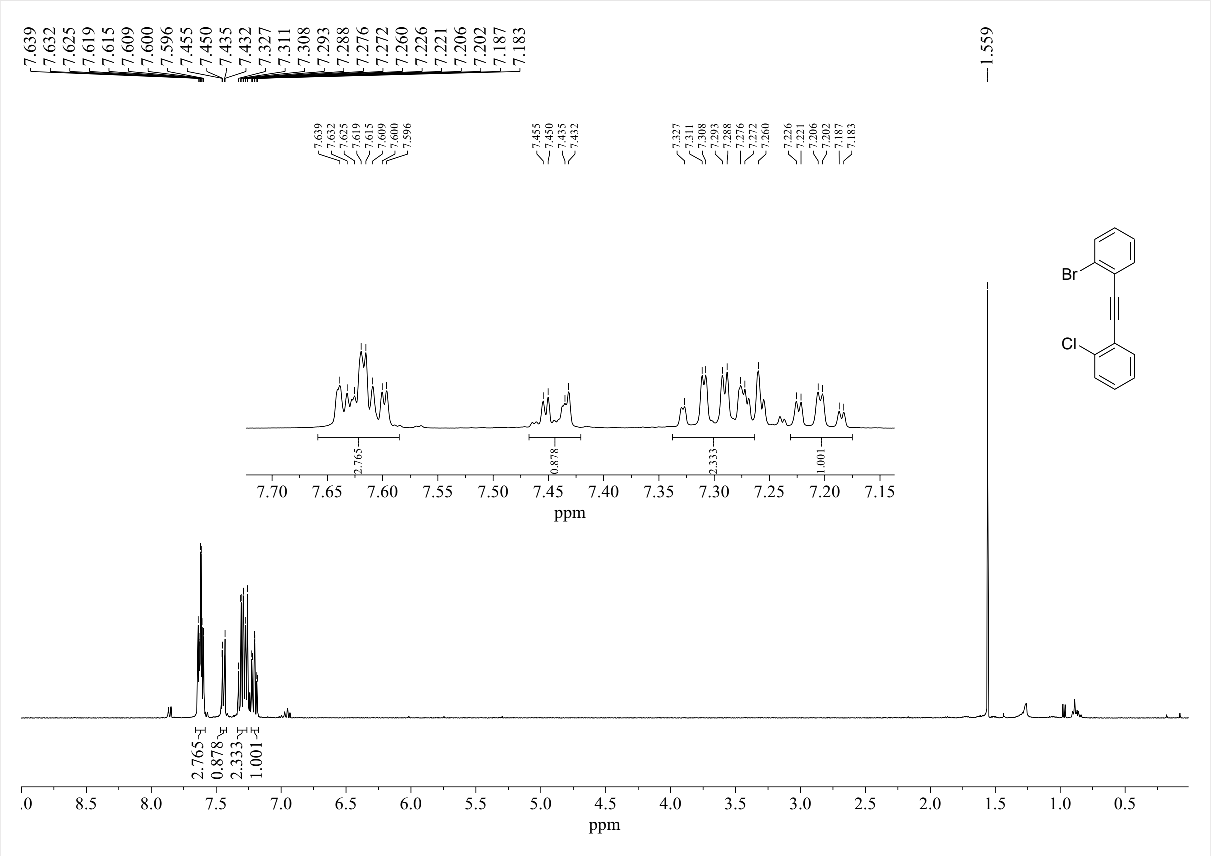

^{1}H NMR spectrum of **S1g** in $CDCl_3$ (400 MHz)

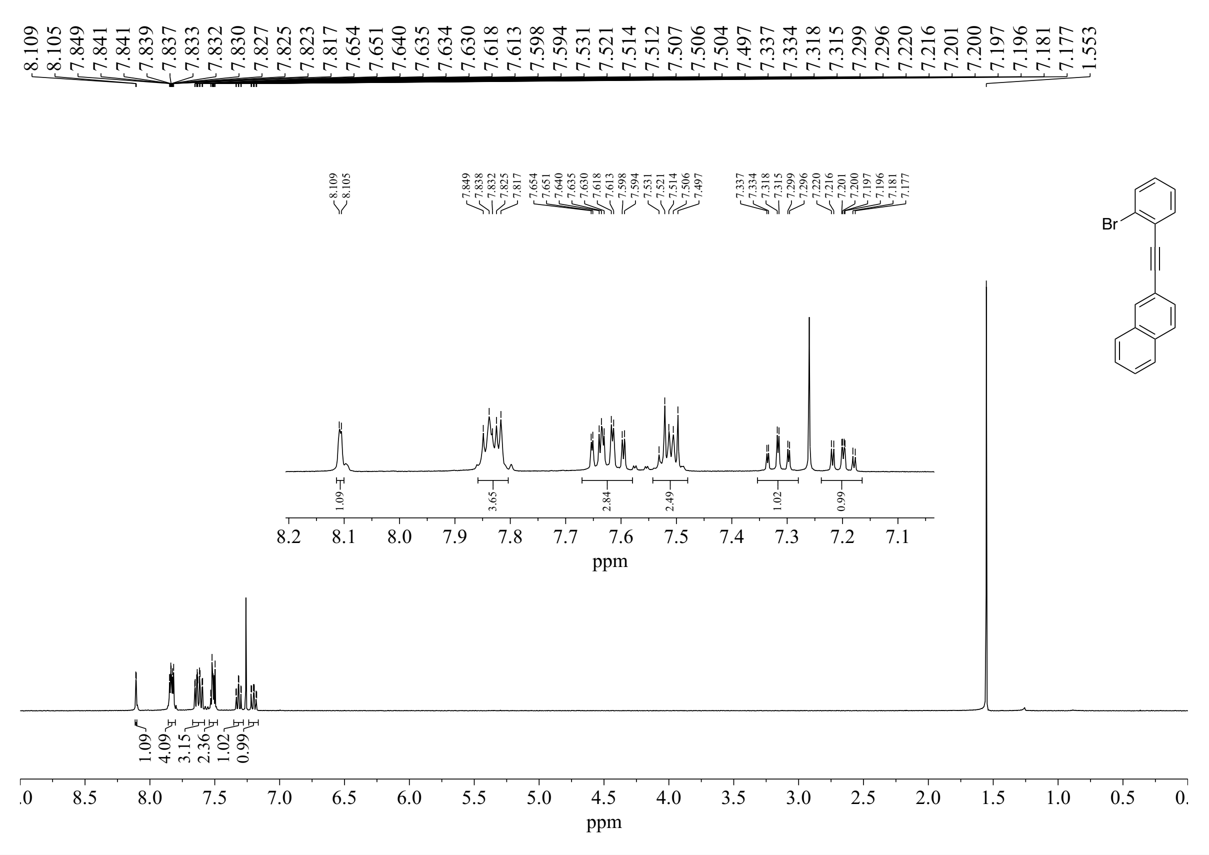

^{1}H NMR spectrum of **S1h** in $CDCl_3$ (400 MHz)

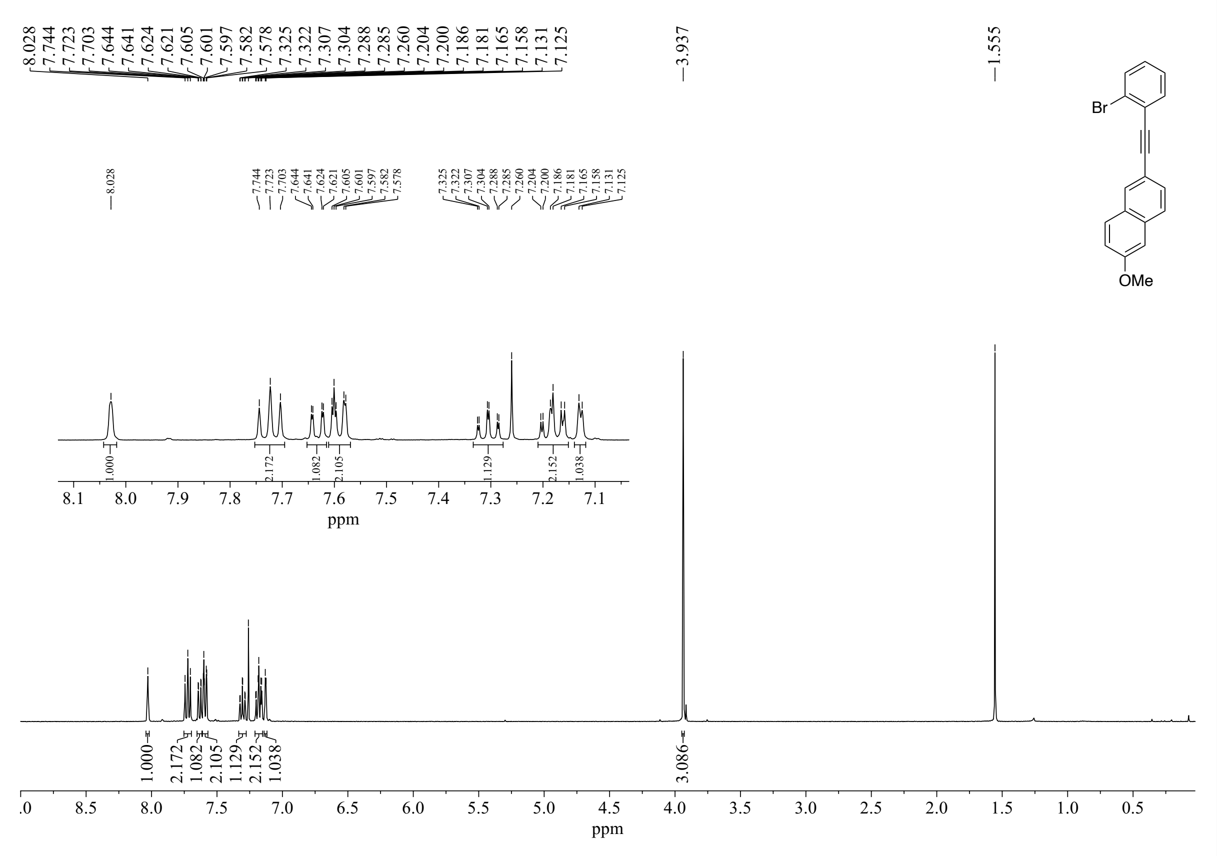

^{1}H (400 MHz) and ^{13}C (125 MHz) NMR spectra of **S1i** in $CDCl_3$

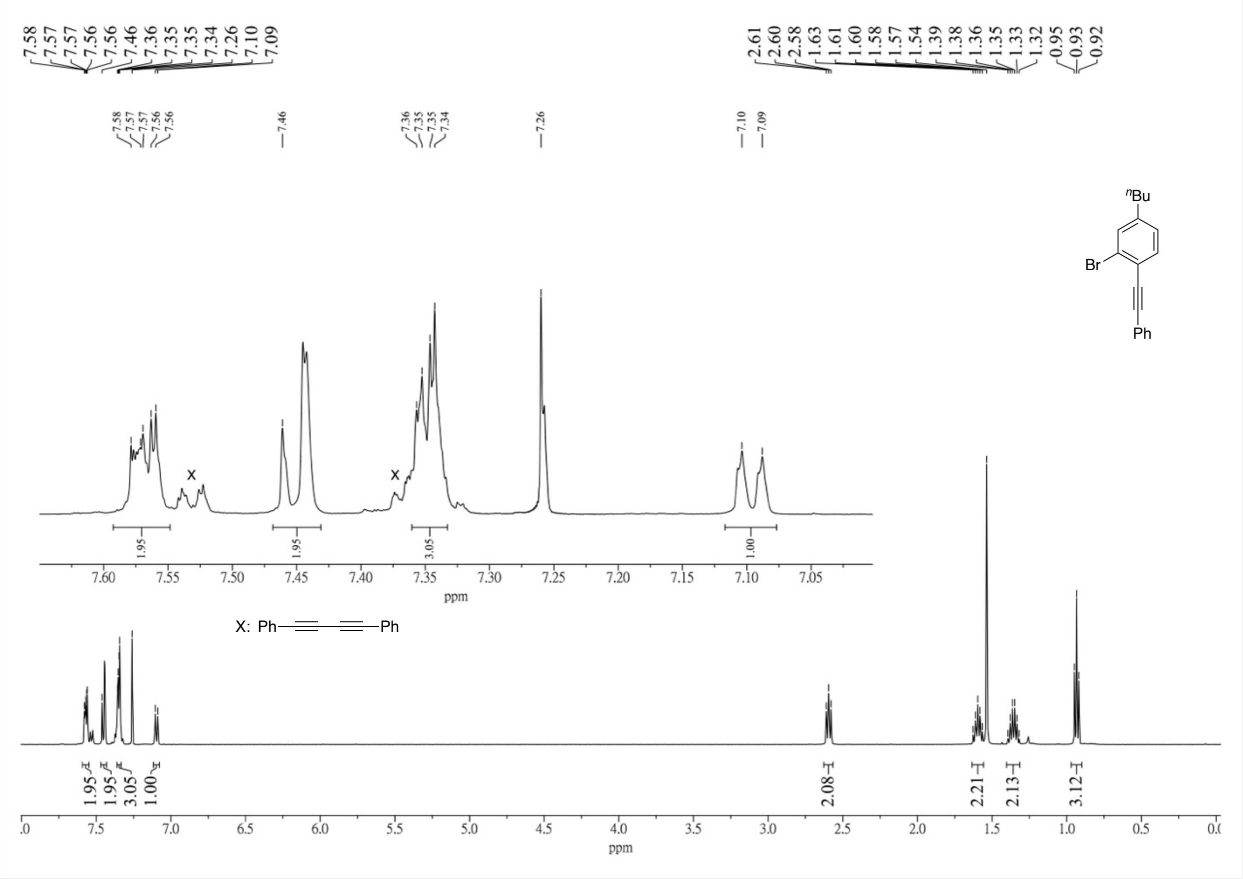

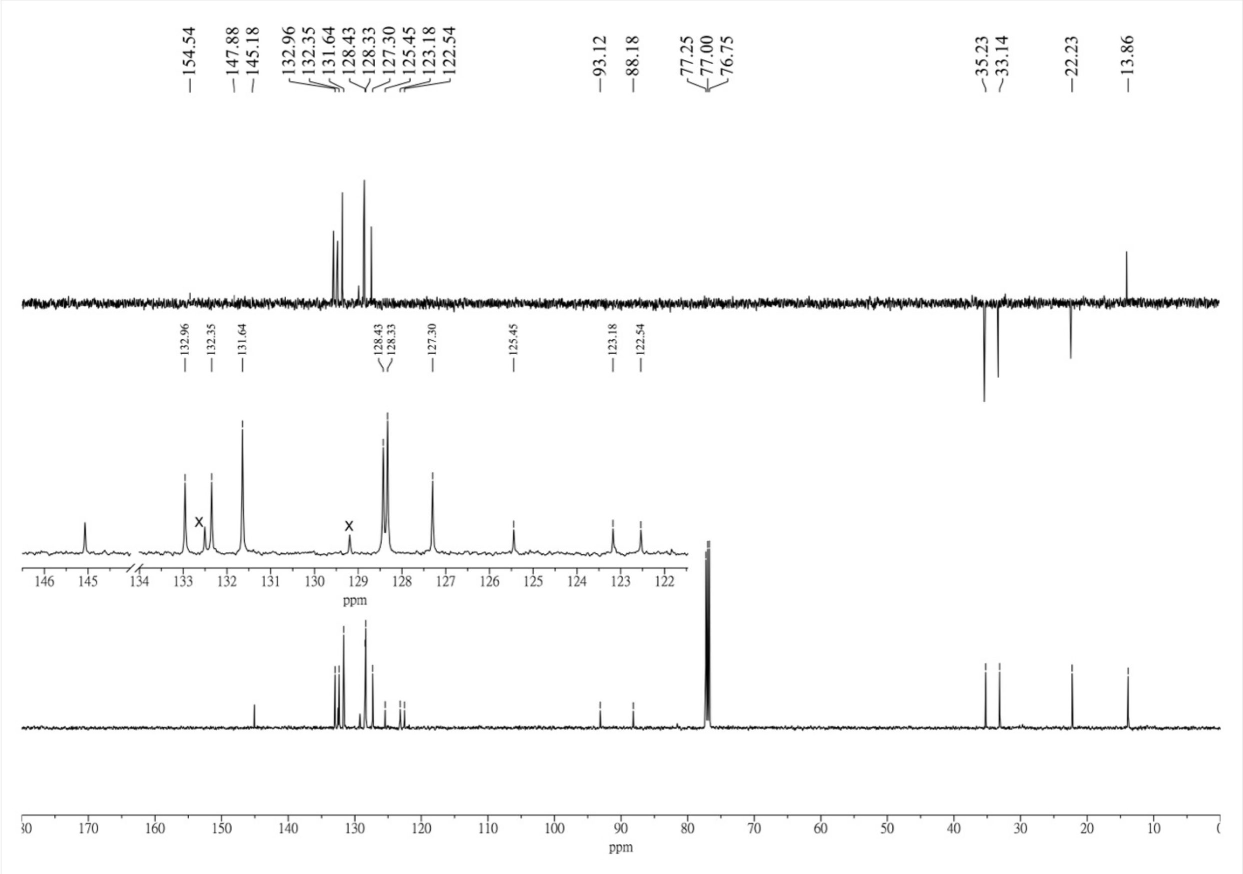

^{1}H NMR spectrum of **S1j** in $CDCl_3$ (400 MHz)

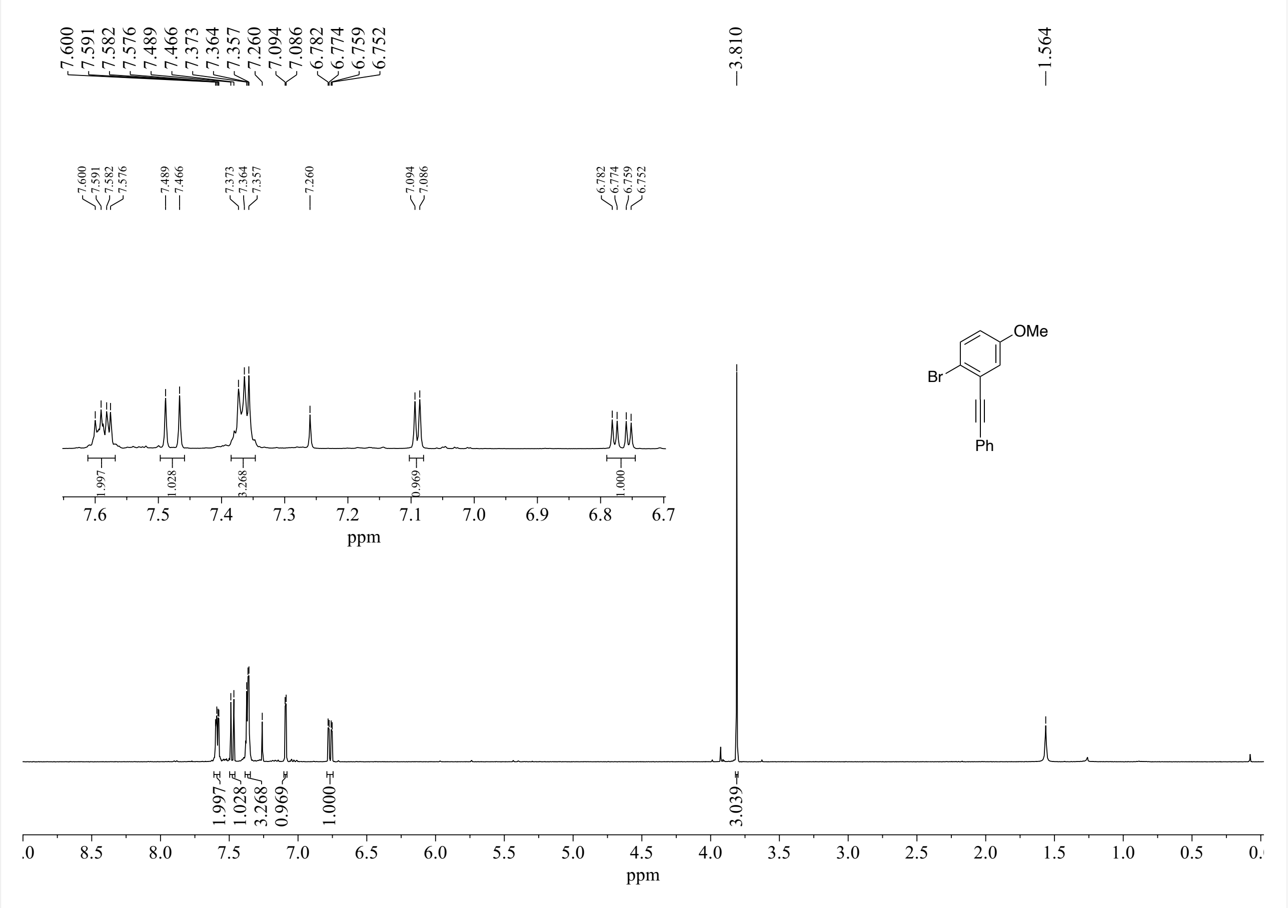

^{1}H (500 MHz) and ^{13}C (125 MHz) NMR spectra of **S1k** in $CDCl_3$

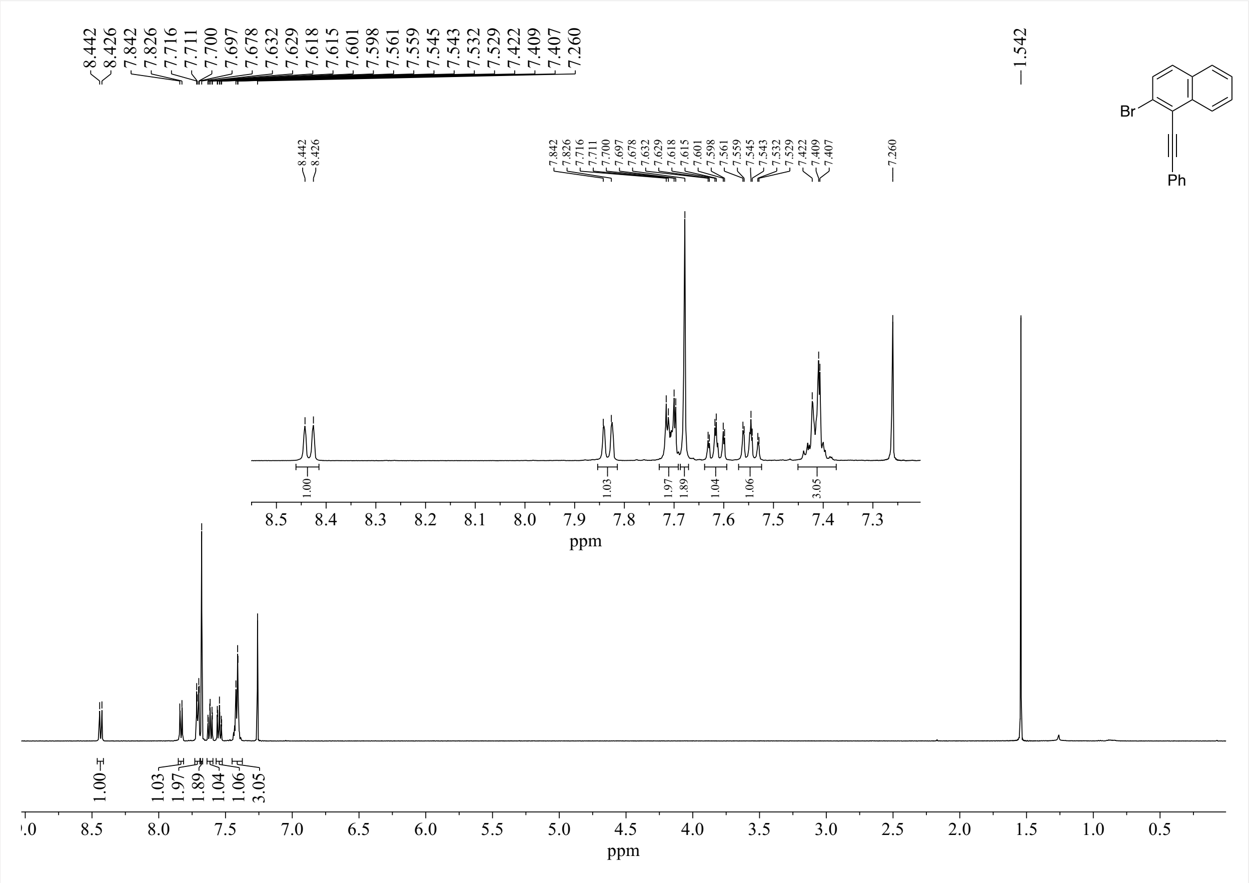

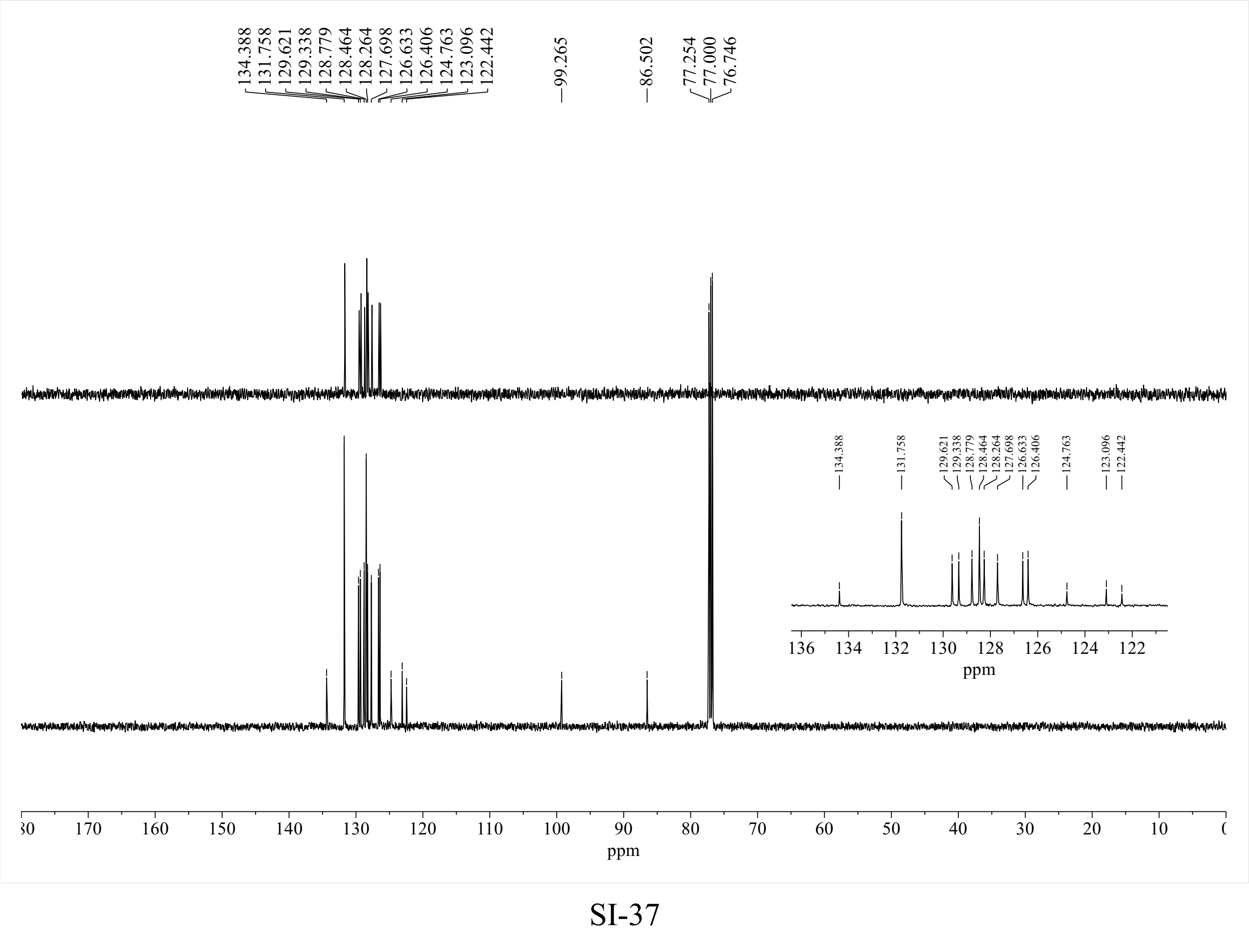

^{1}H NMR spectrum of **1a** in $CDCl_3$ (400 MHz)

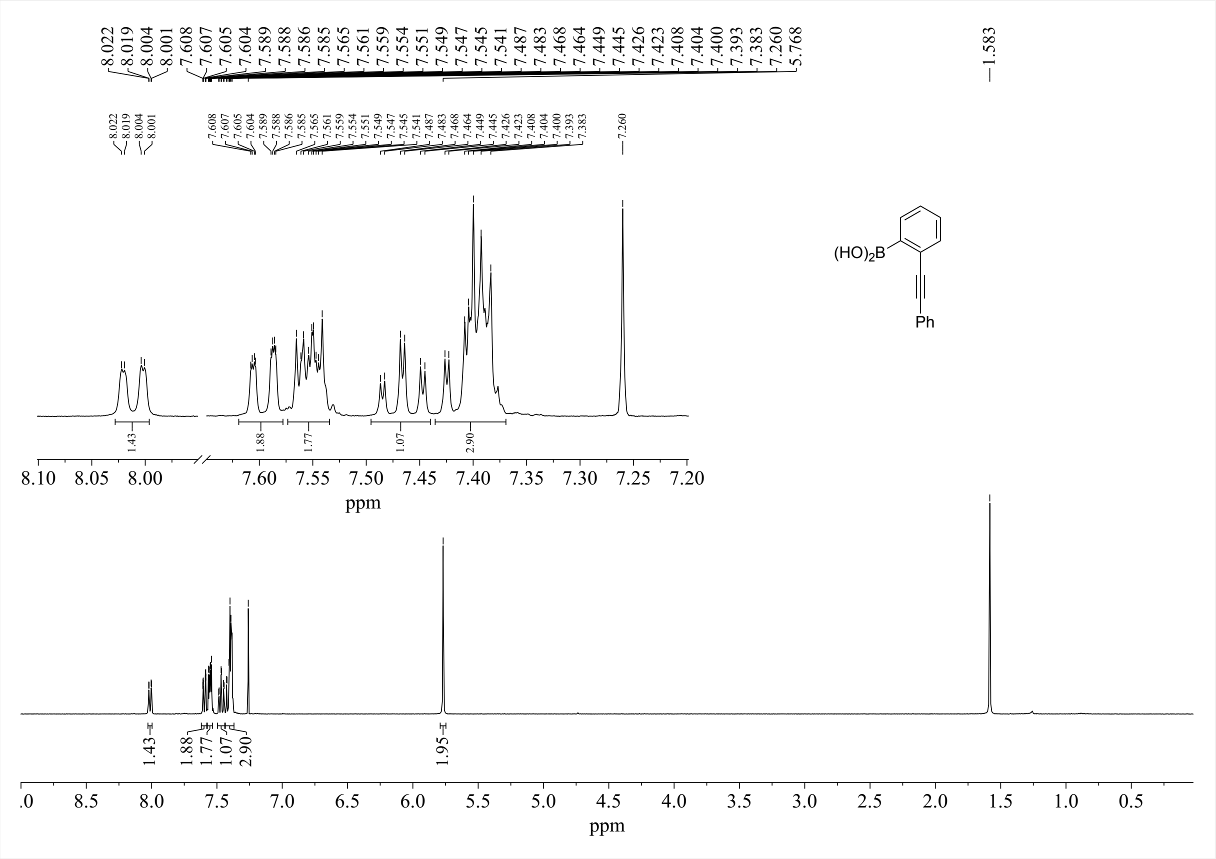

^{1}H NMR spectrum of **1b** in $CDCl_3$ (400 MHz)

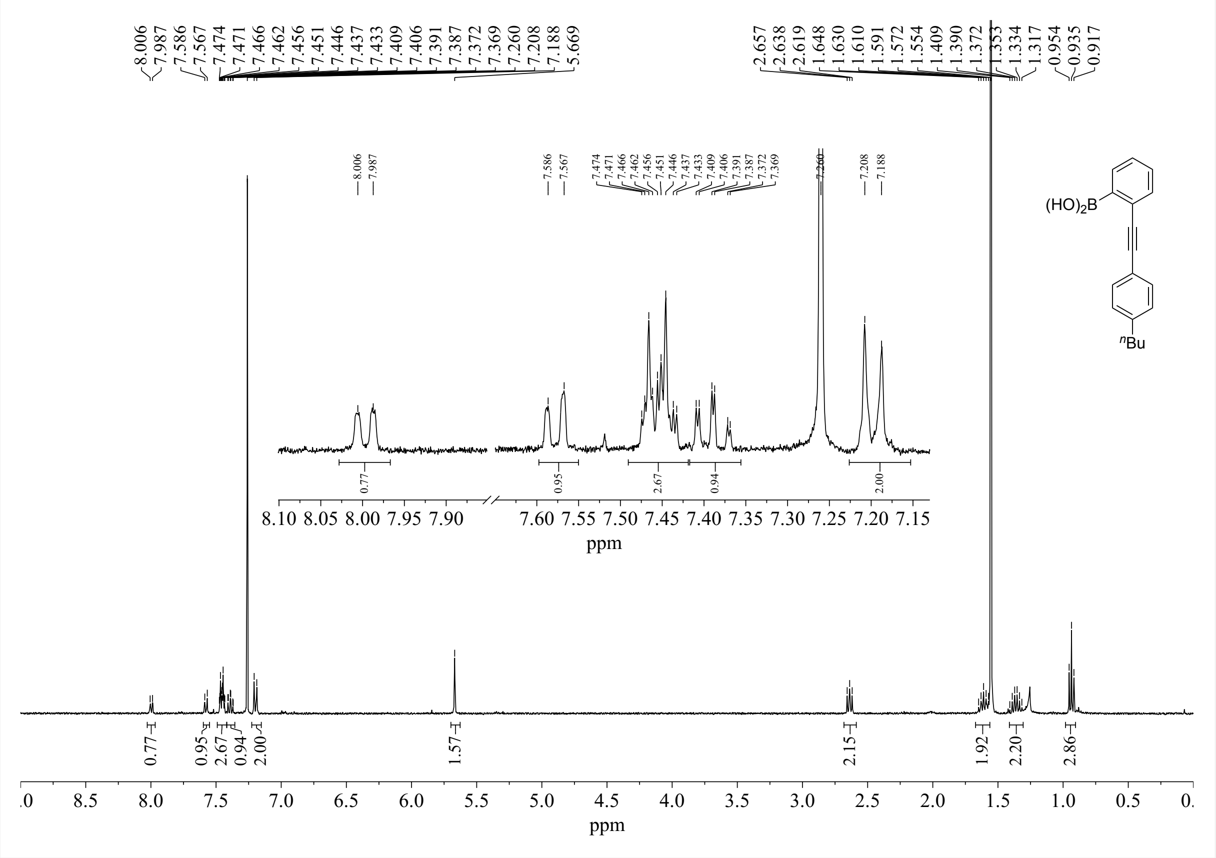

^{1}H NMR spectrum of **1c** in $CDCl_3$ (500 MHz)

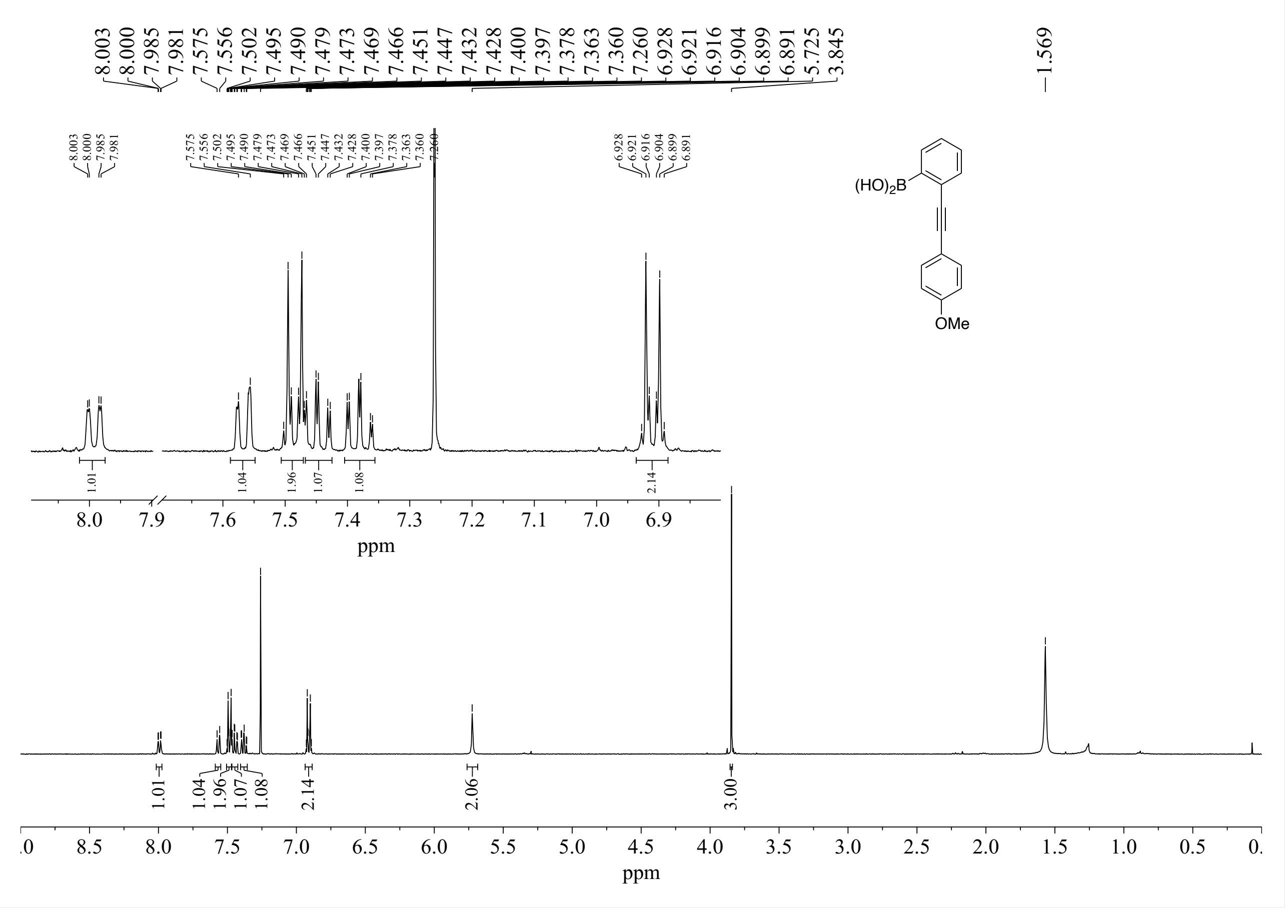

^{1}H (400 MHz) and ^{13}C (125 MHz) NMR spectra of **1d** in $CDCl_3$

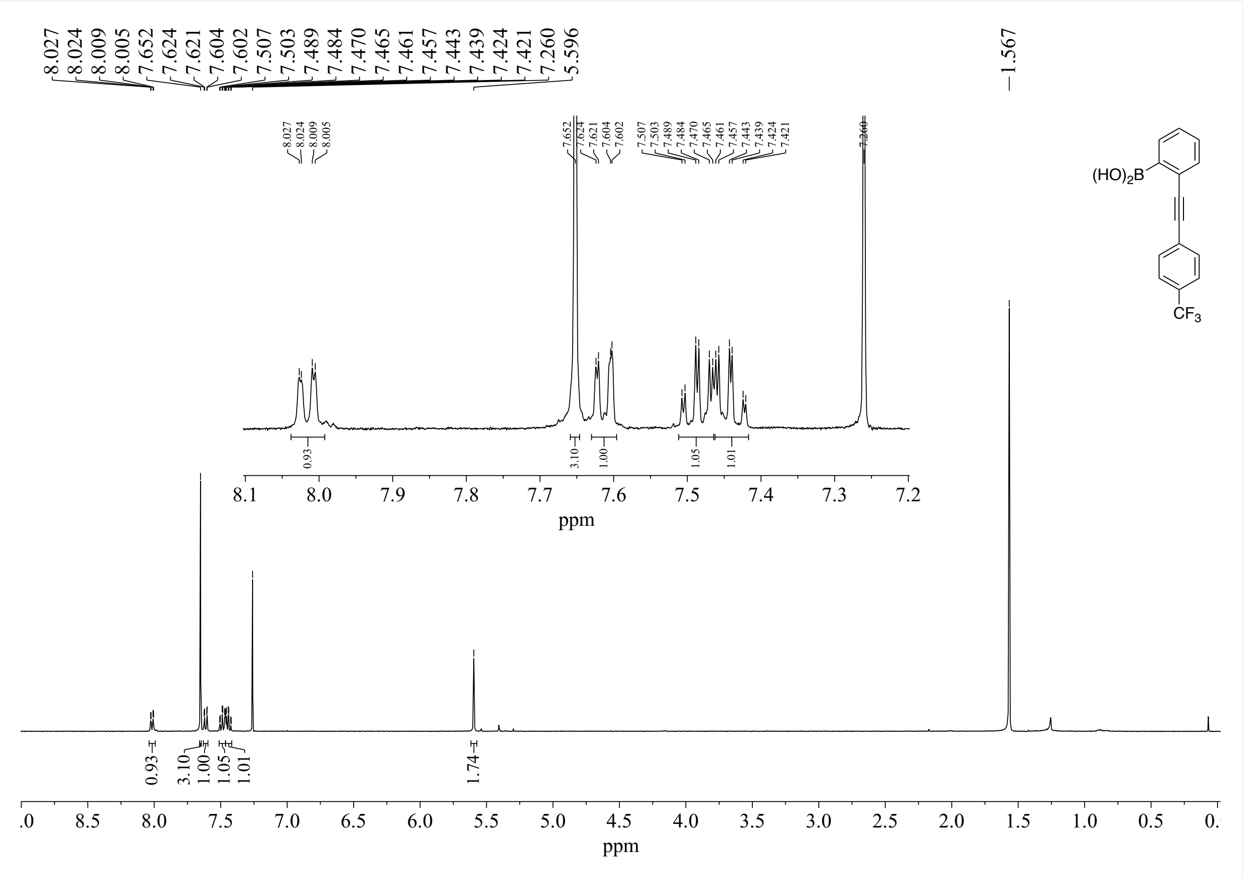

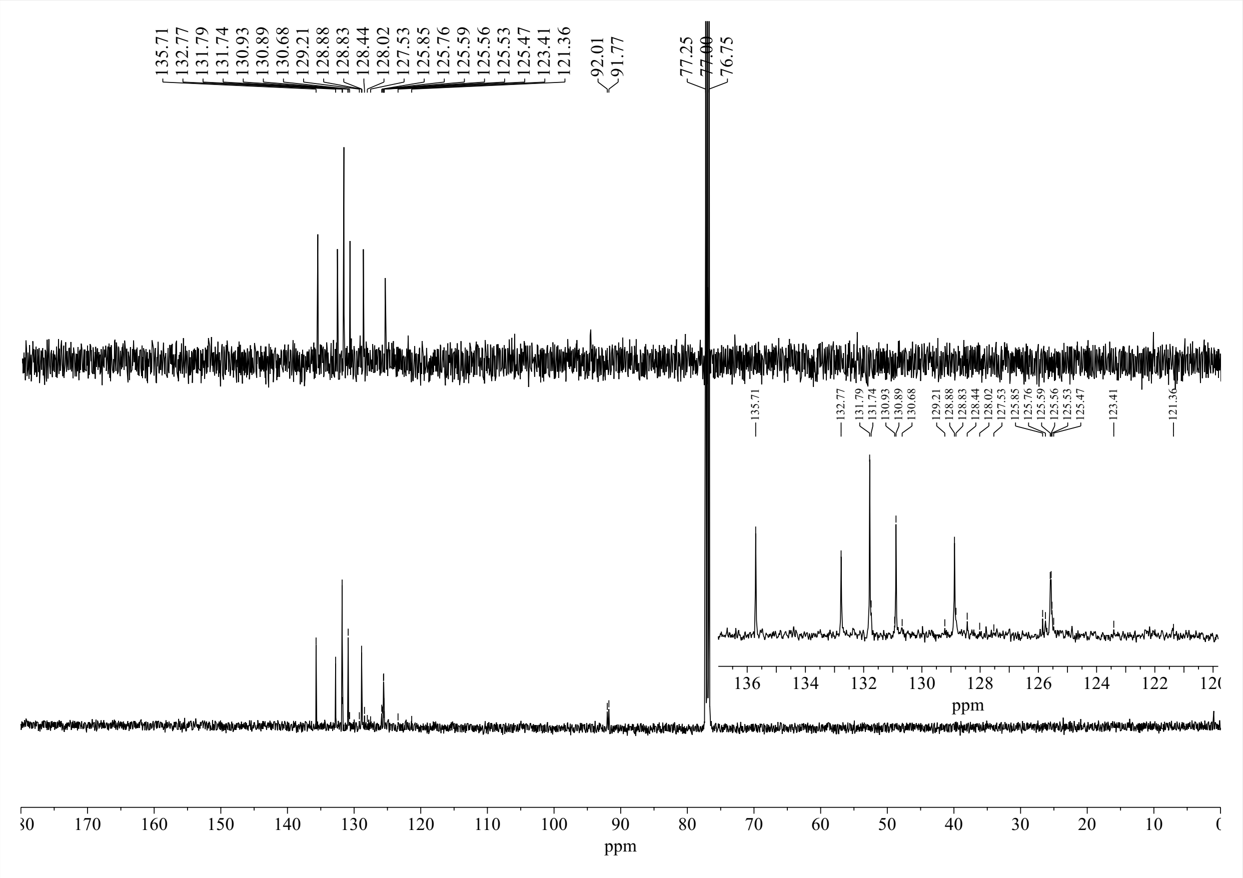

^{1}H NMR spectrum of **1e** in $CDCl_3$ (400 MHz)

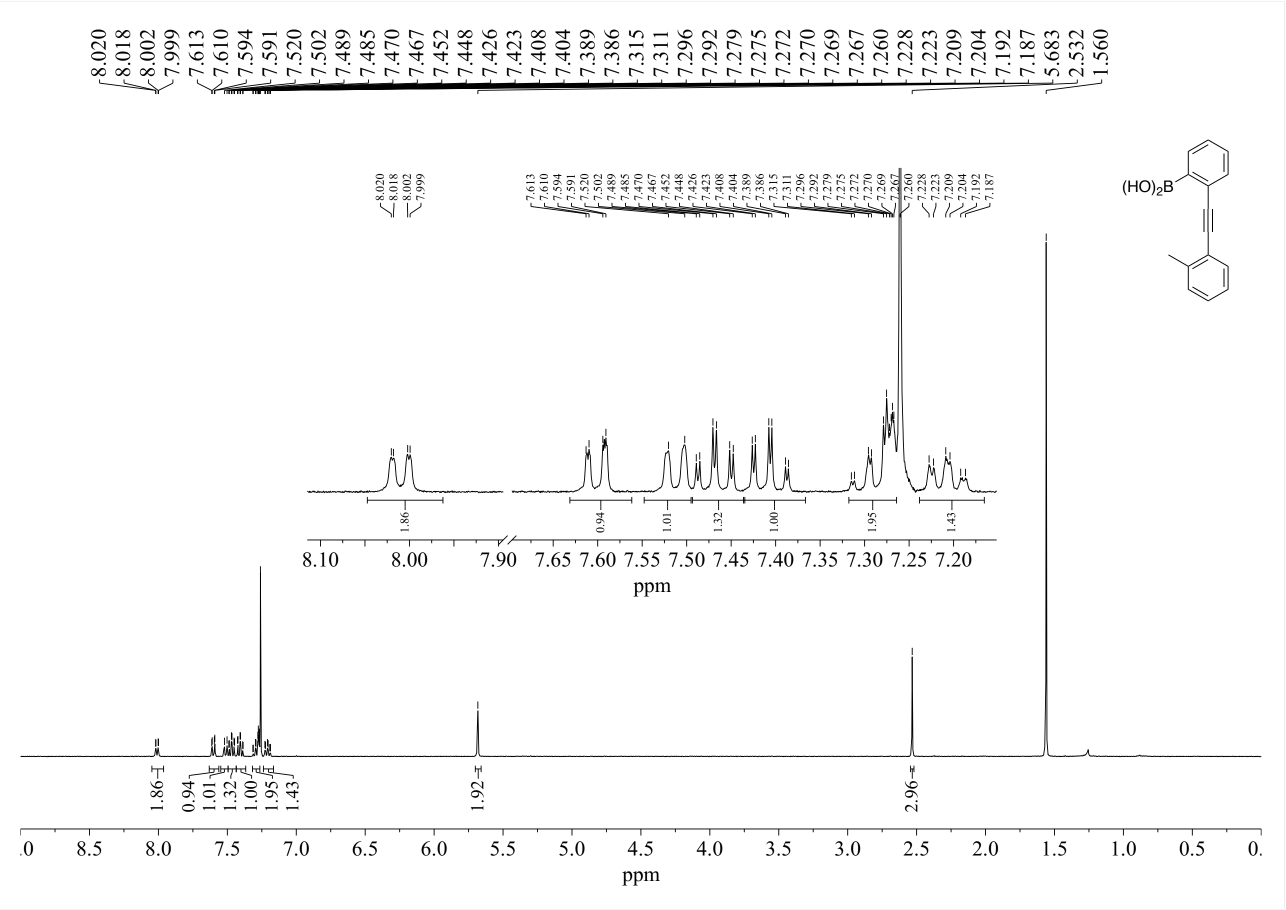

^{1}H (400 MHz) and ^{13}C (125 MHz) NMR spectrum of **1f** in $CDCl_3$

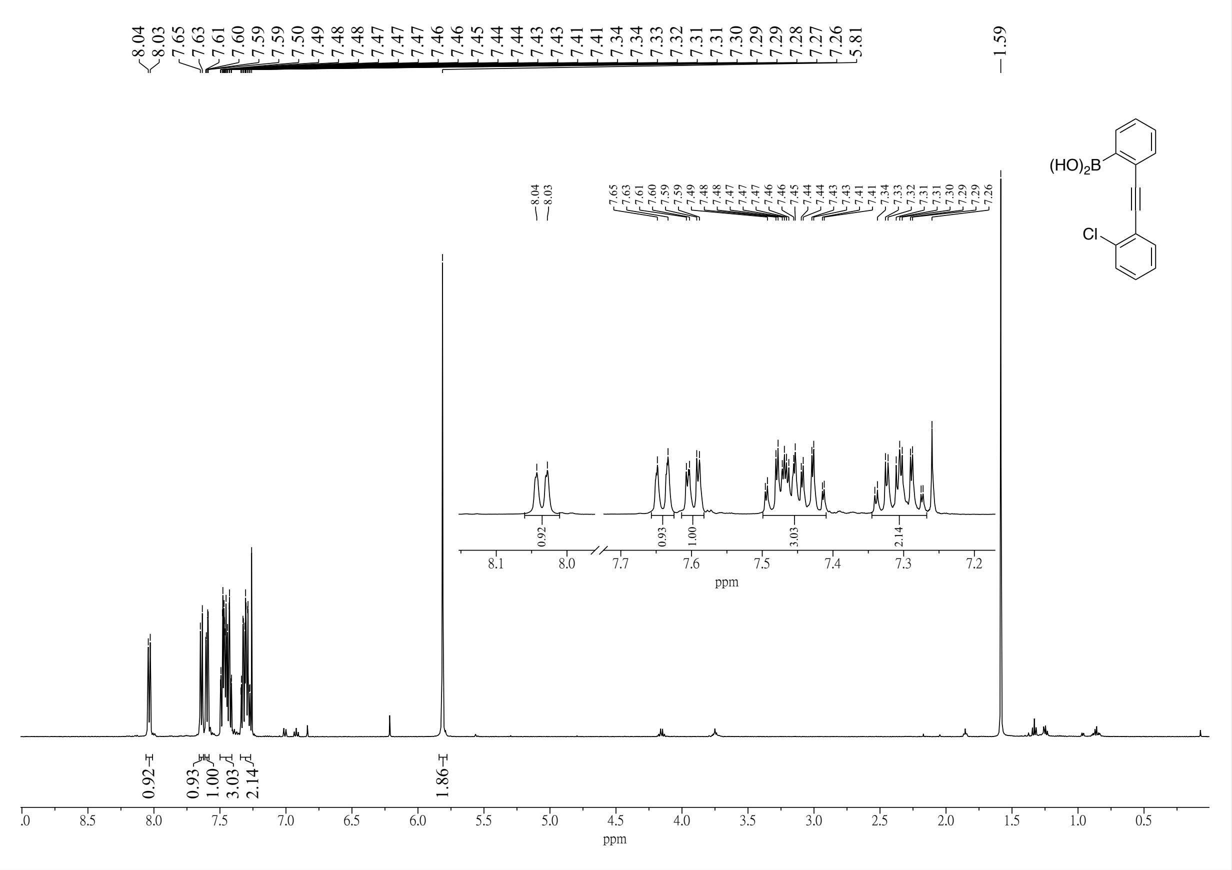

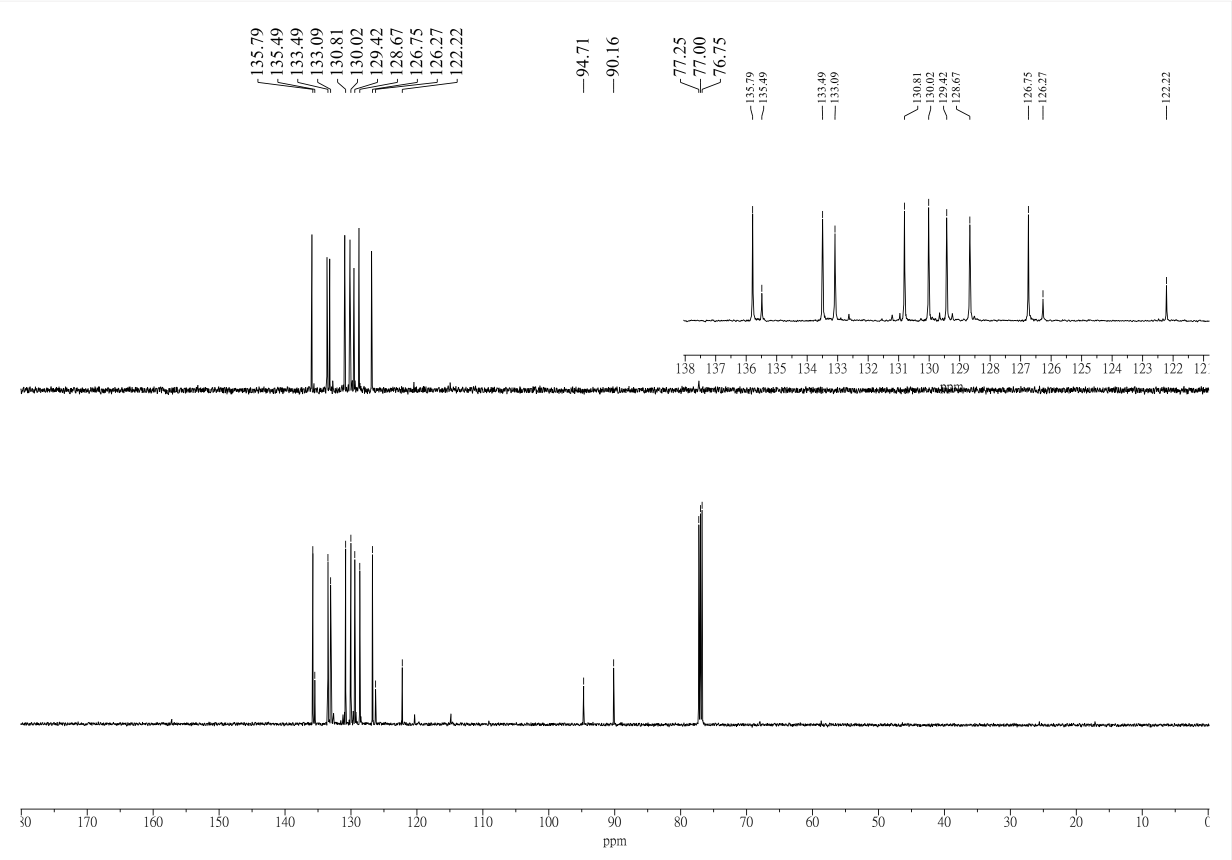

^{1}H NMR spectrum of **1g** in $CDCl_3$ (400 MHz)

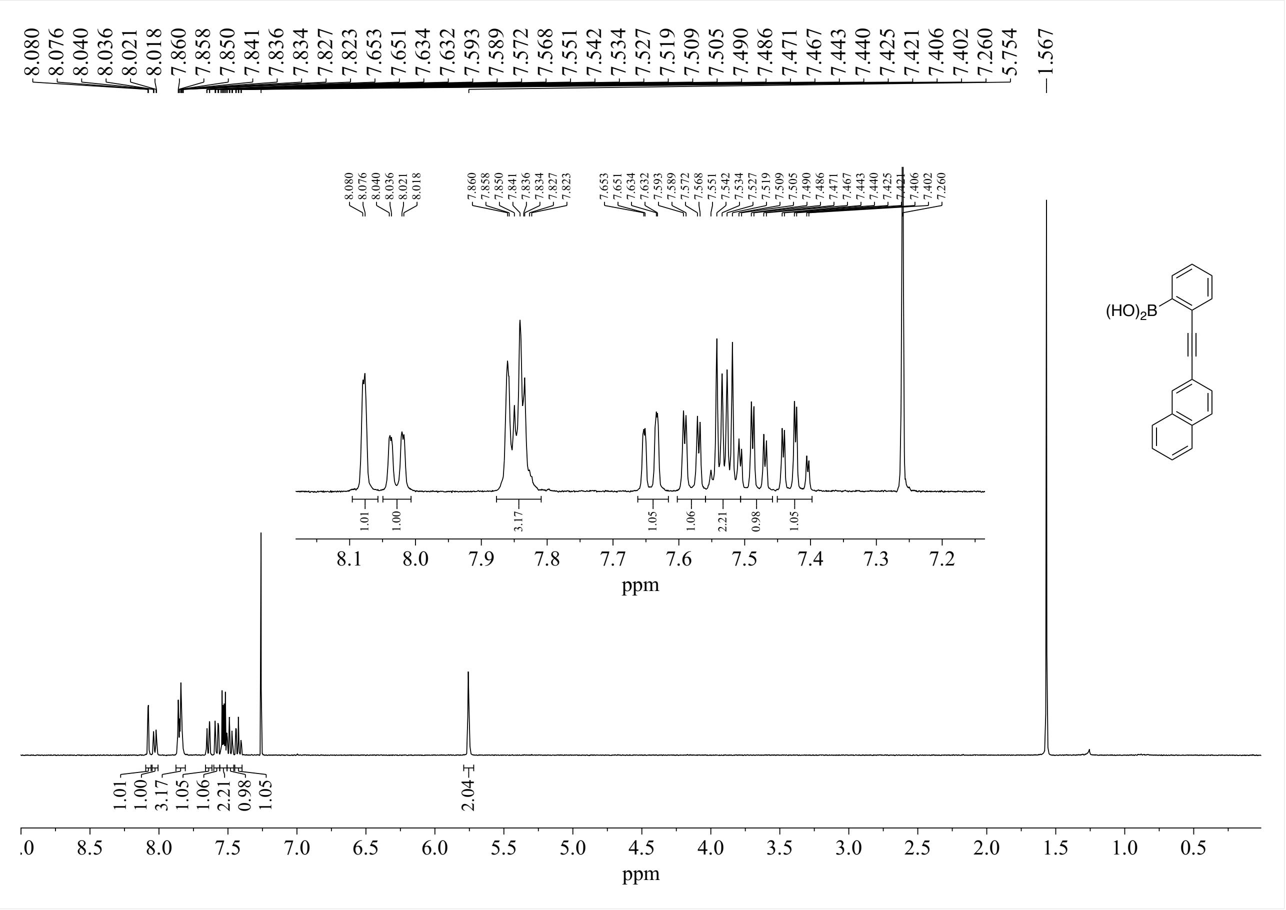

^{1}H (400 MHz) and ^{13}C (125 MHz) NMR spectra of **1h** in $CDCl_3$

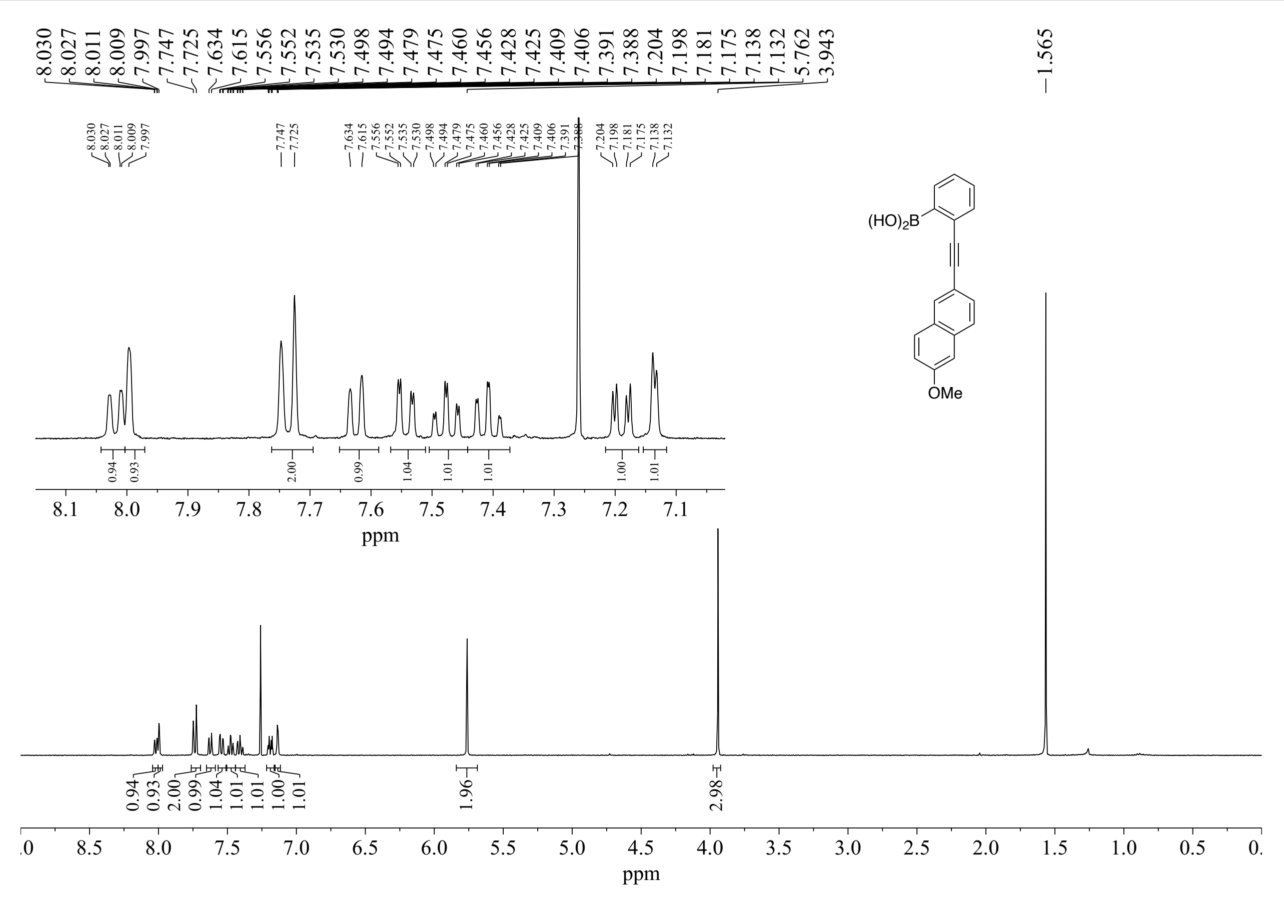

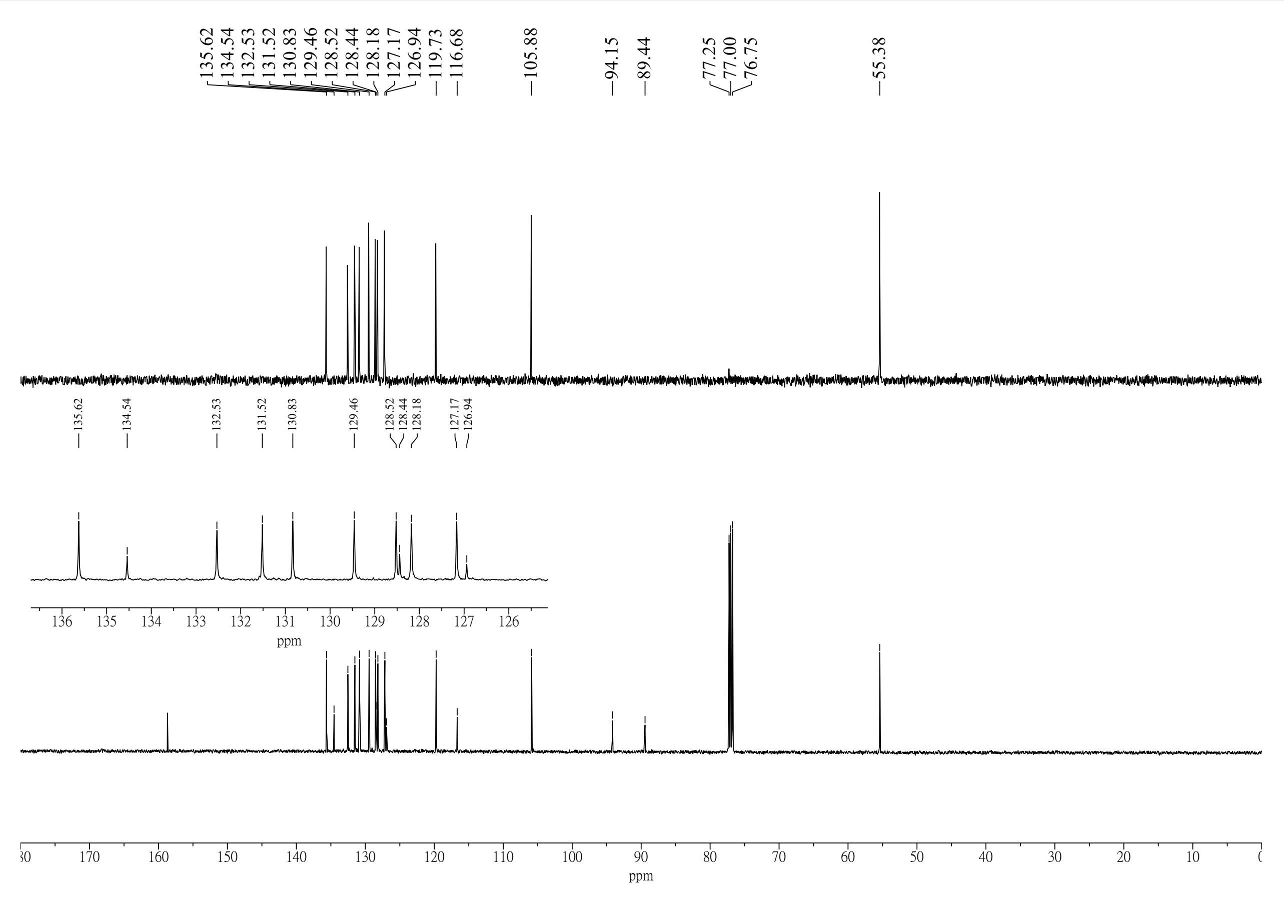

^{1}H (400 MHz) and ^{13}C (125 MHz) NMR spectra of **1i** in $CDCl_3$

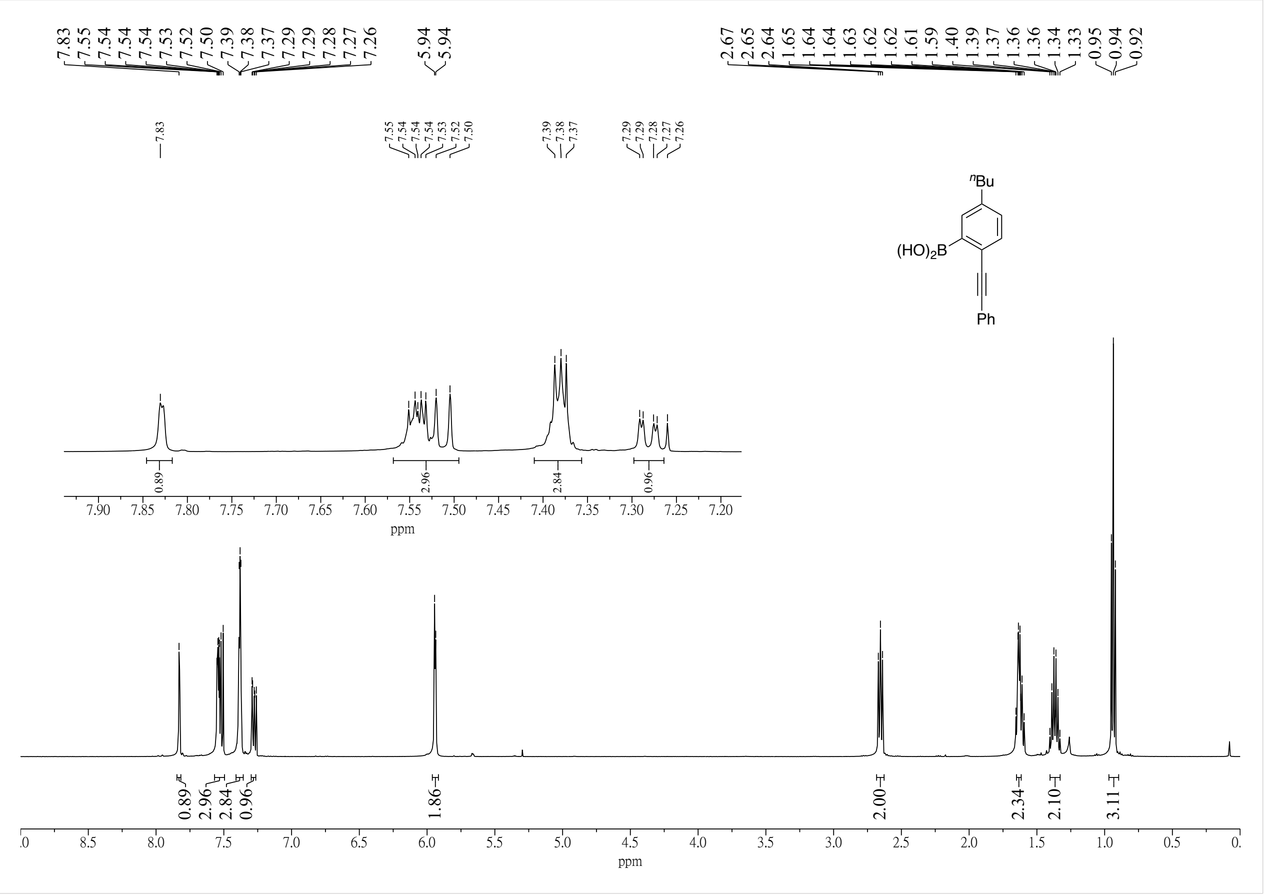

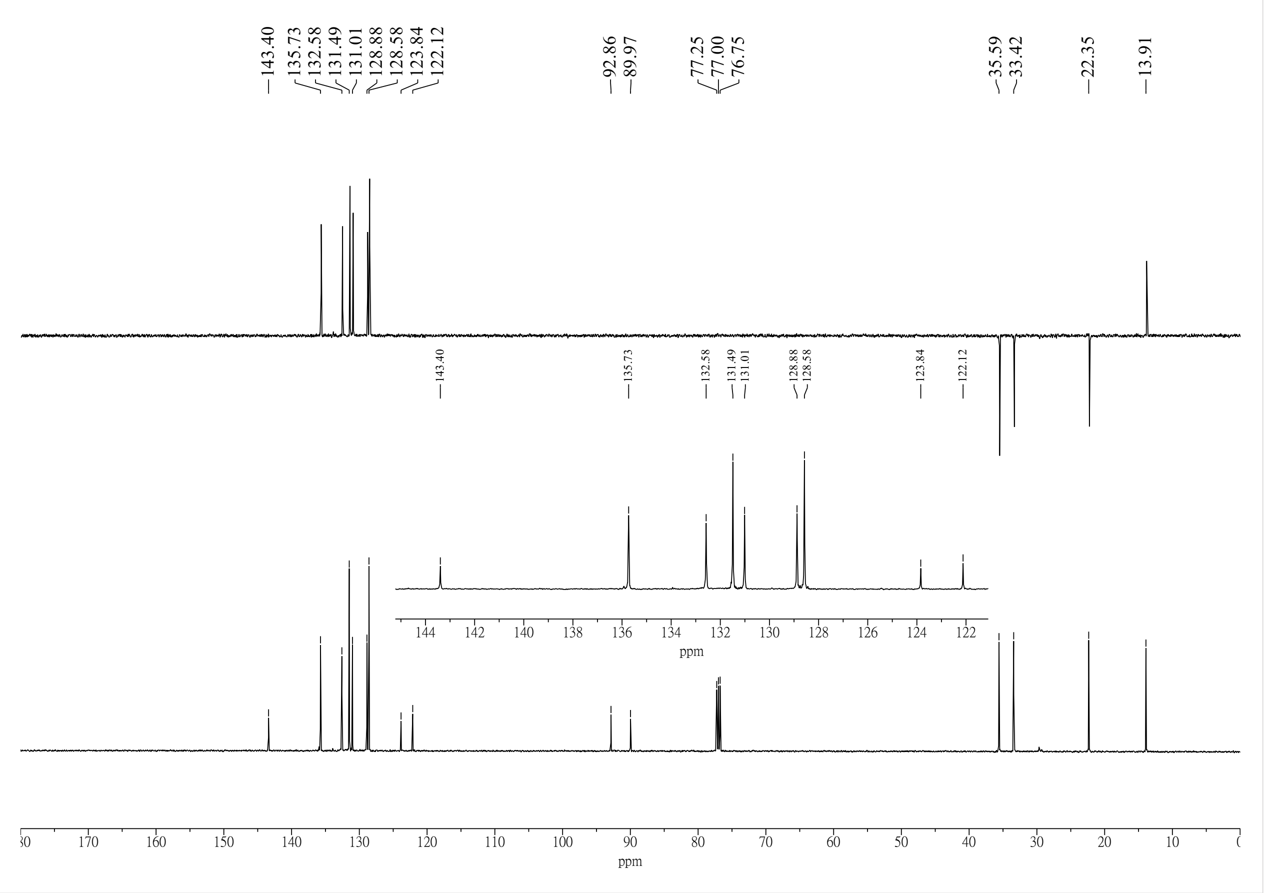

^{1}H NMR spectrum of **1j** in $CDCl_3$ (400 MHz)

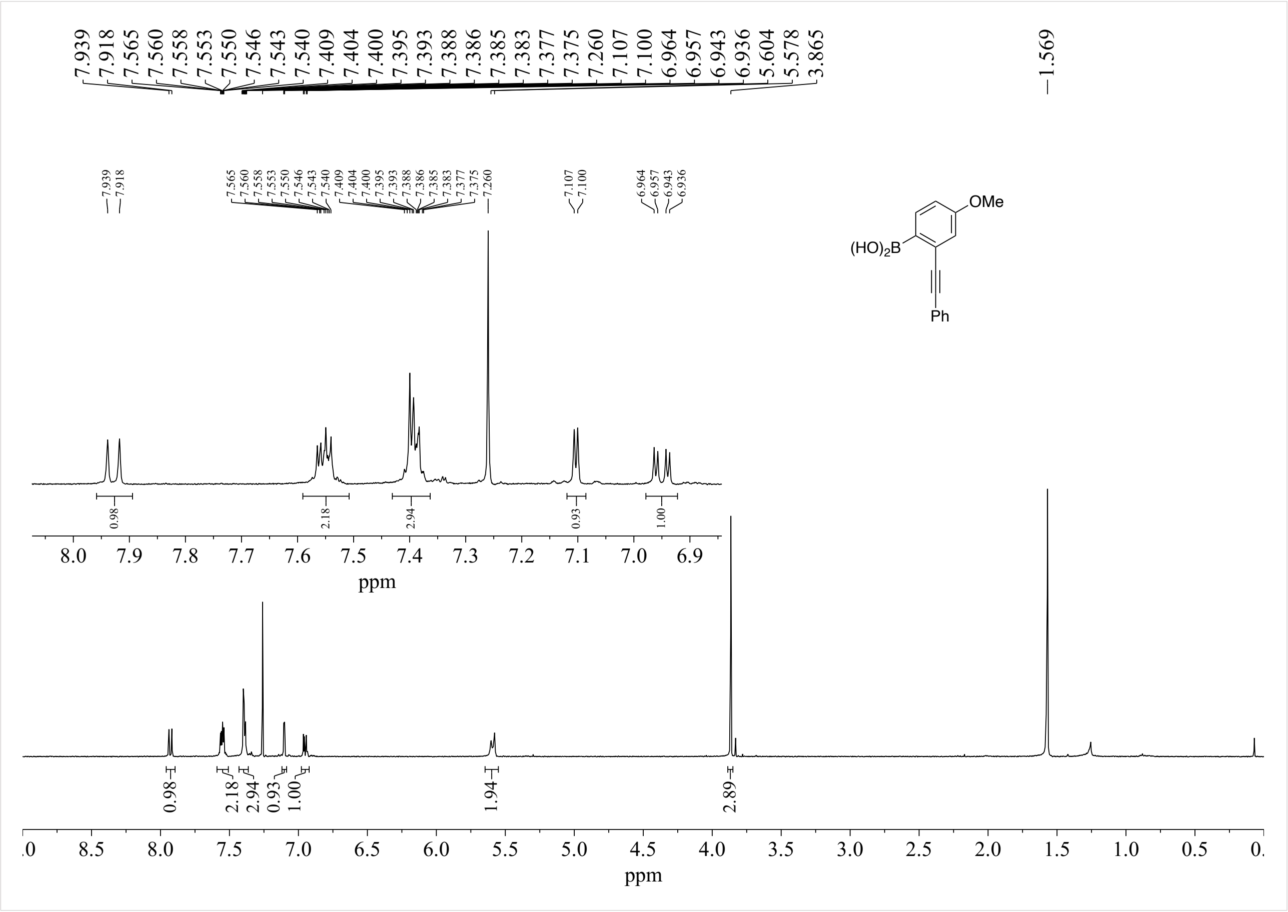

^{1}H (400 MHz) and ^{13}C (125 MHz) NMR spectra of **1k** in $CDCl_3$

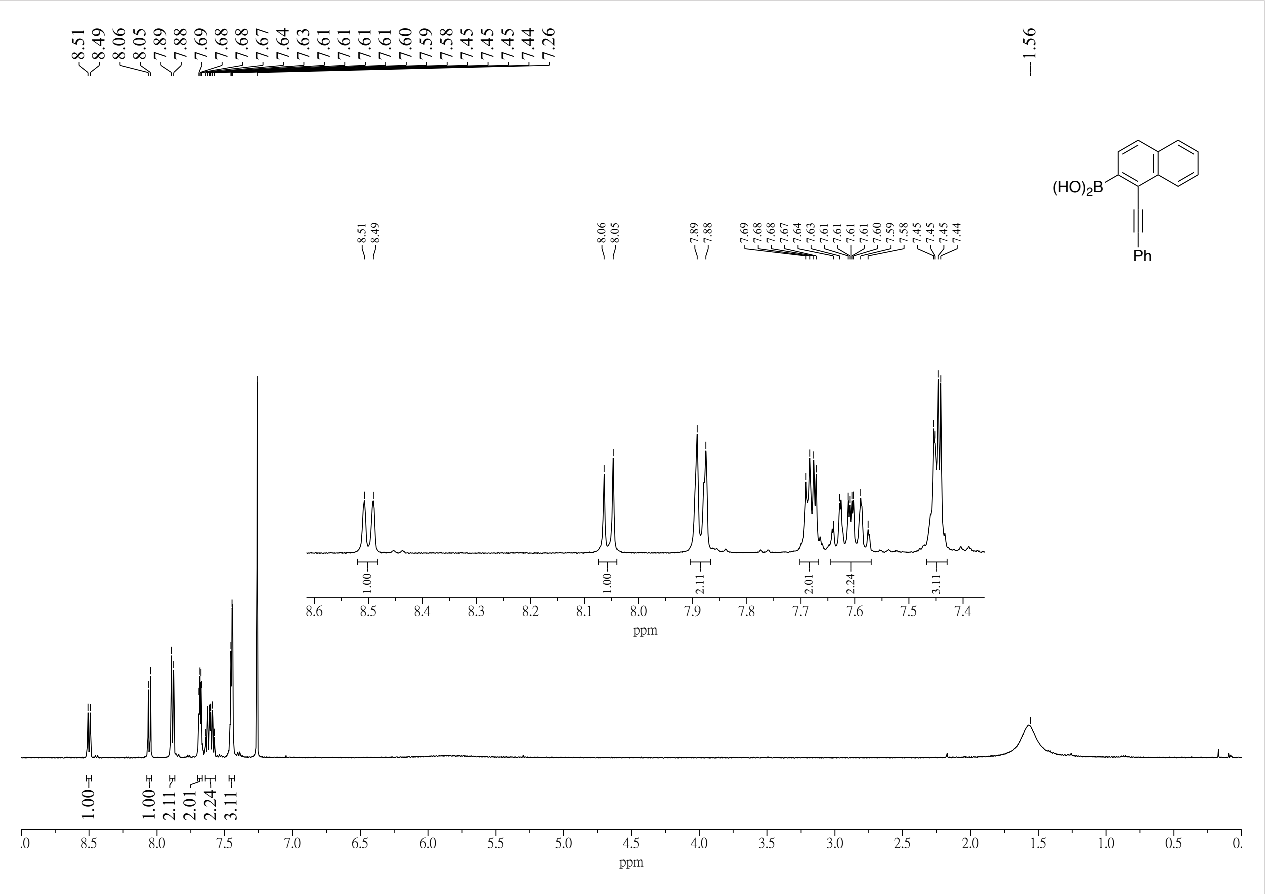

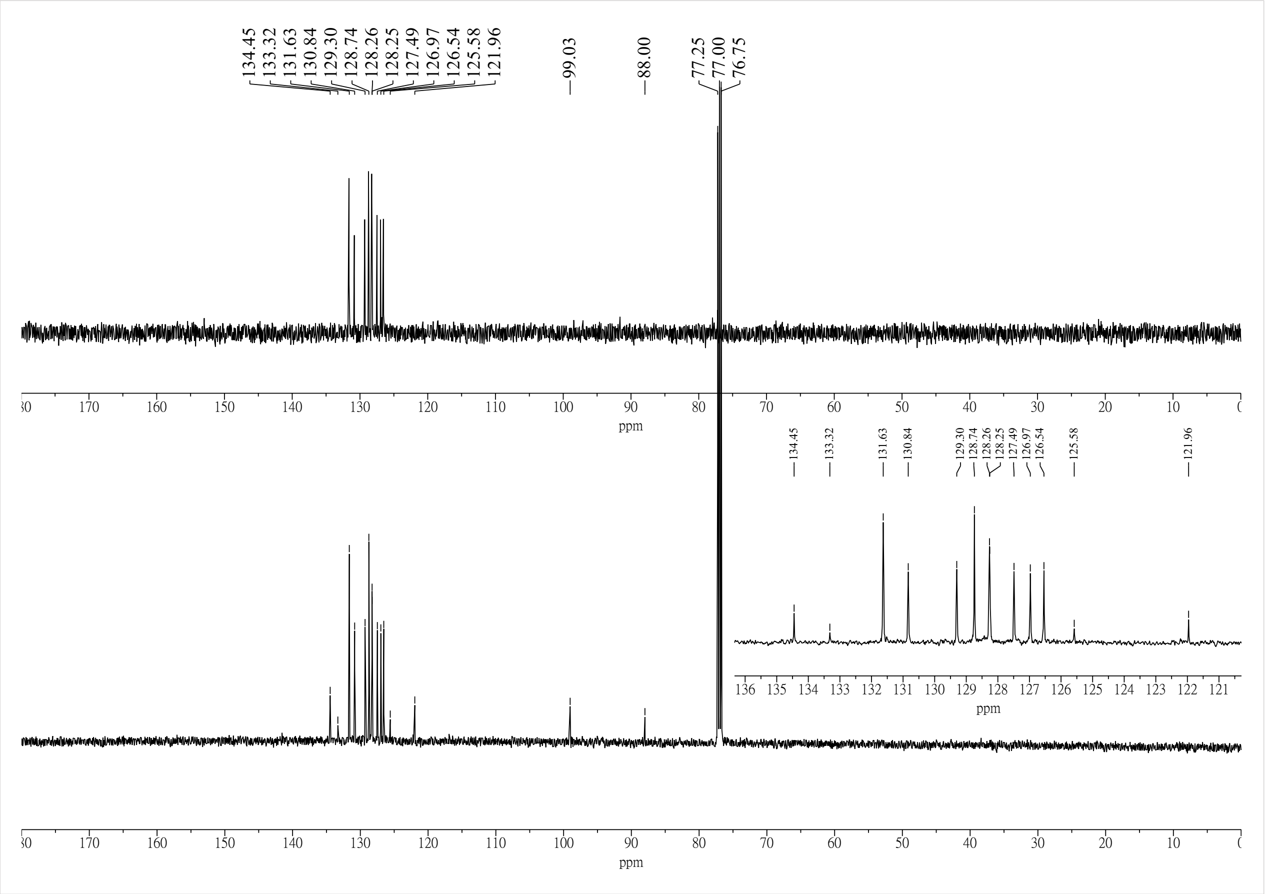

^{1}H (500 MHz) and ^{13}C (125 MHz) NMR spectra of **2a** in $CDCl_3$

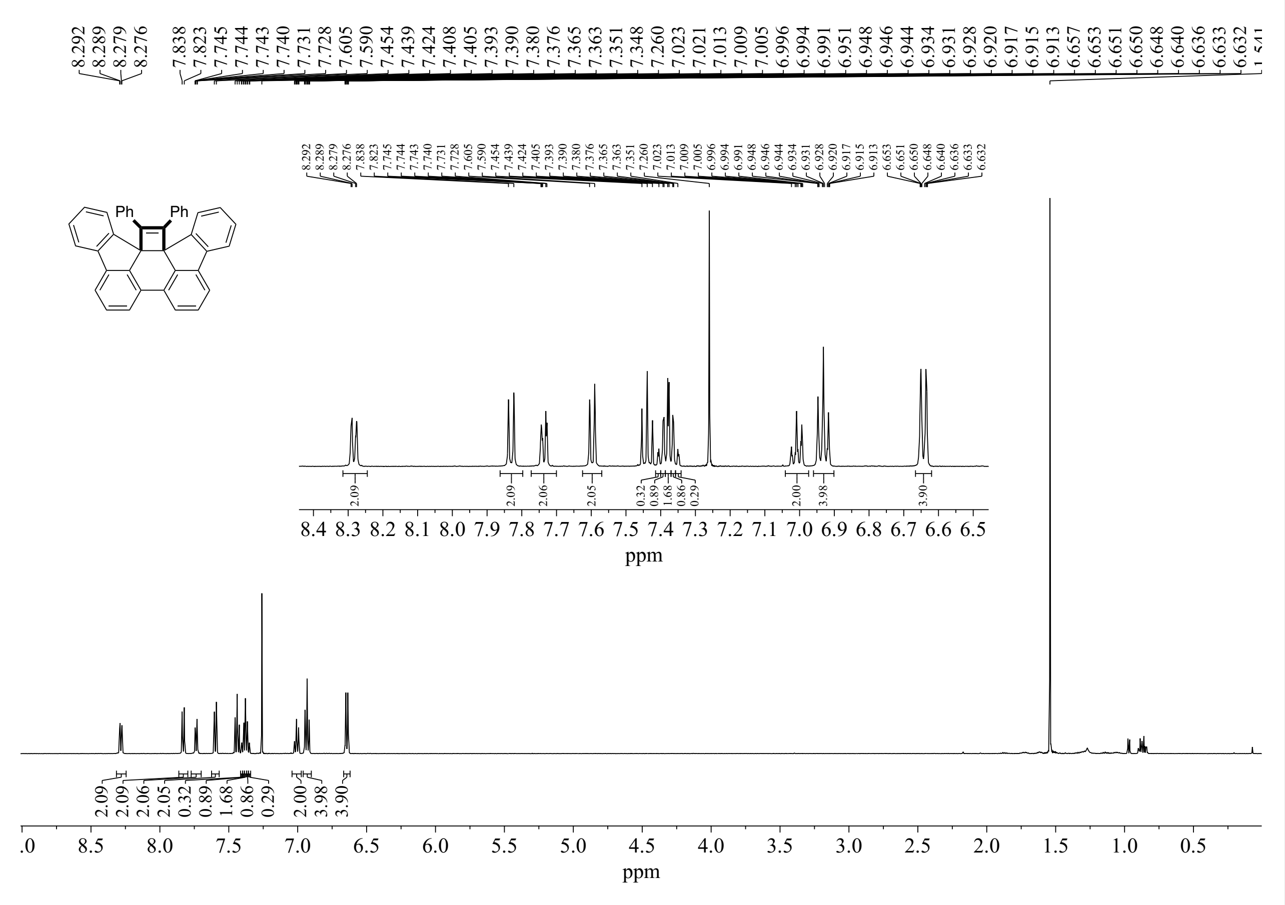

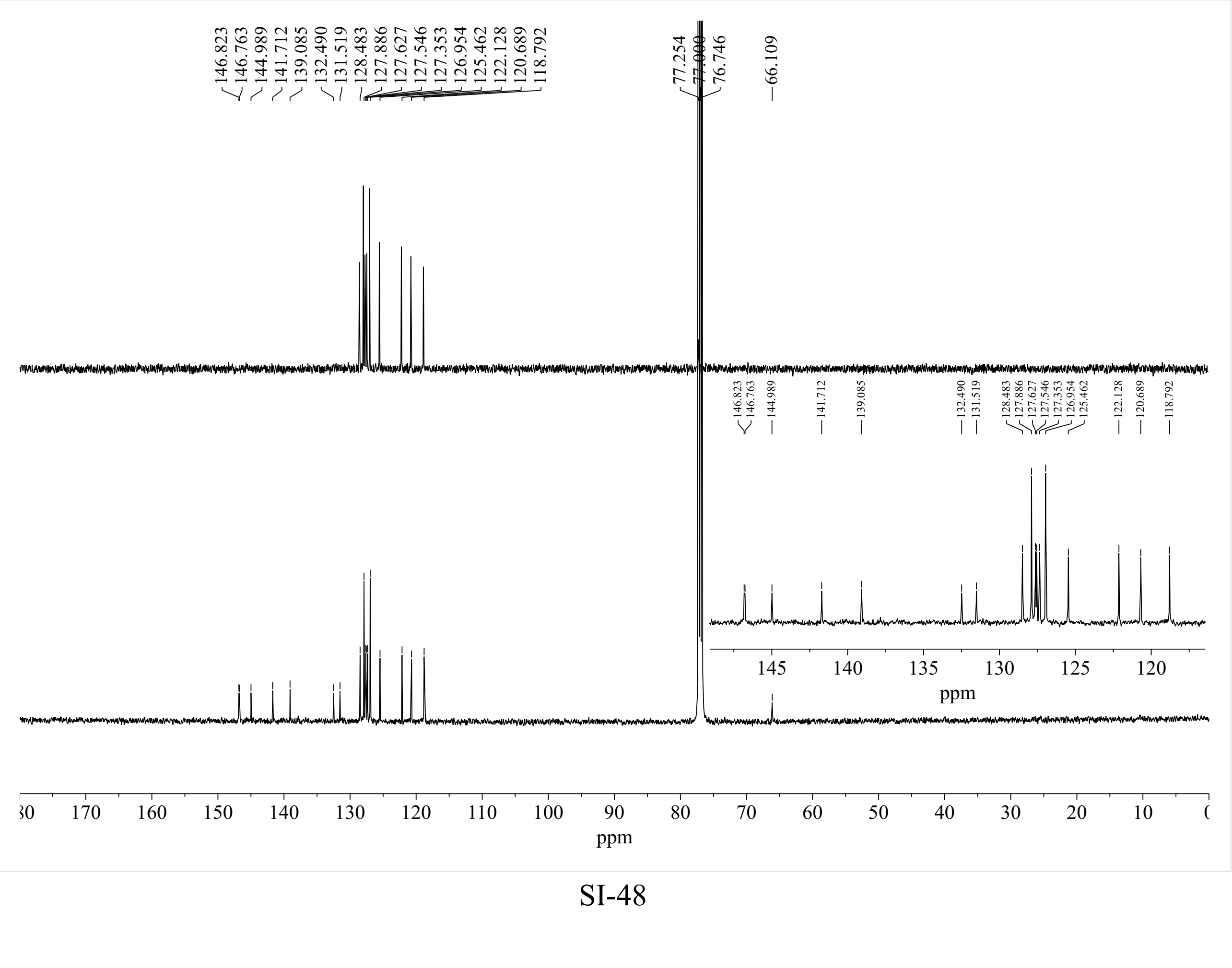

^{1}H (500 MHz) and ^{13}C (125 MHz) NMR spectra of **2b** in $CDCl_3$

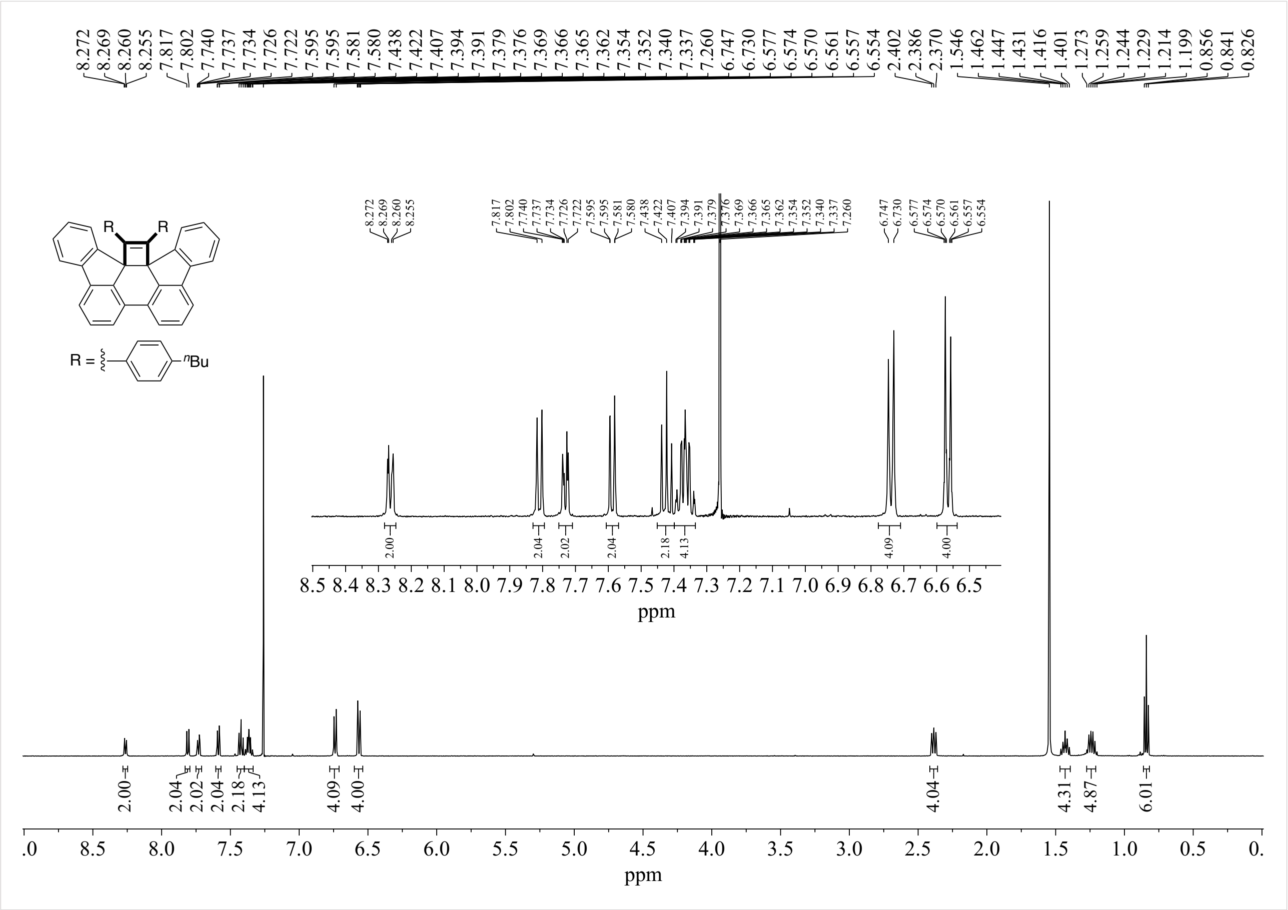

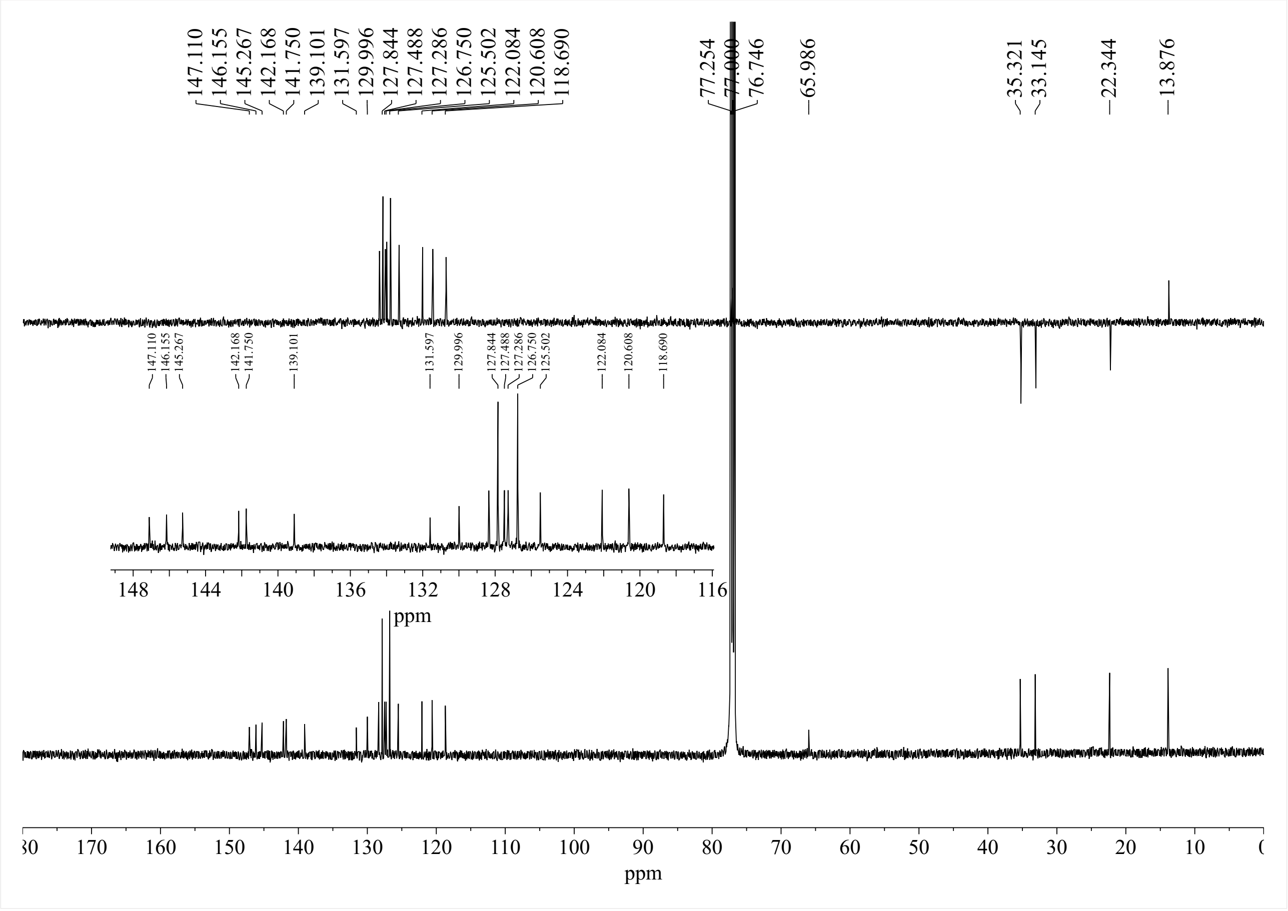

^{1}H (500 MHz) and ^{13}C (125 MHz) NMR spectra of **2c** in $CDCl_3$

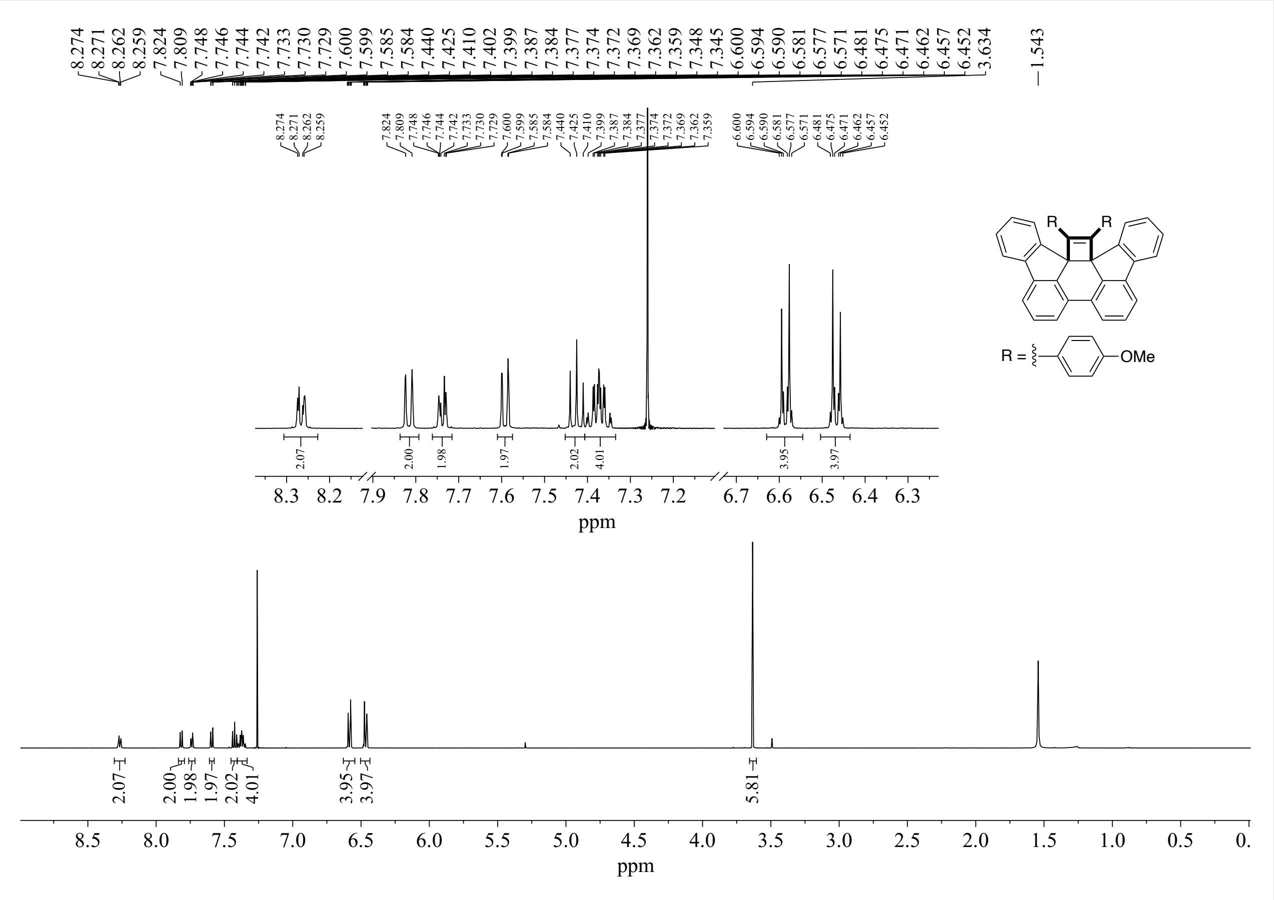

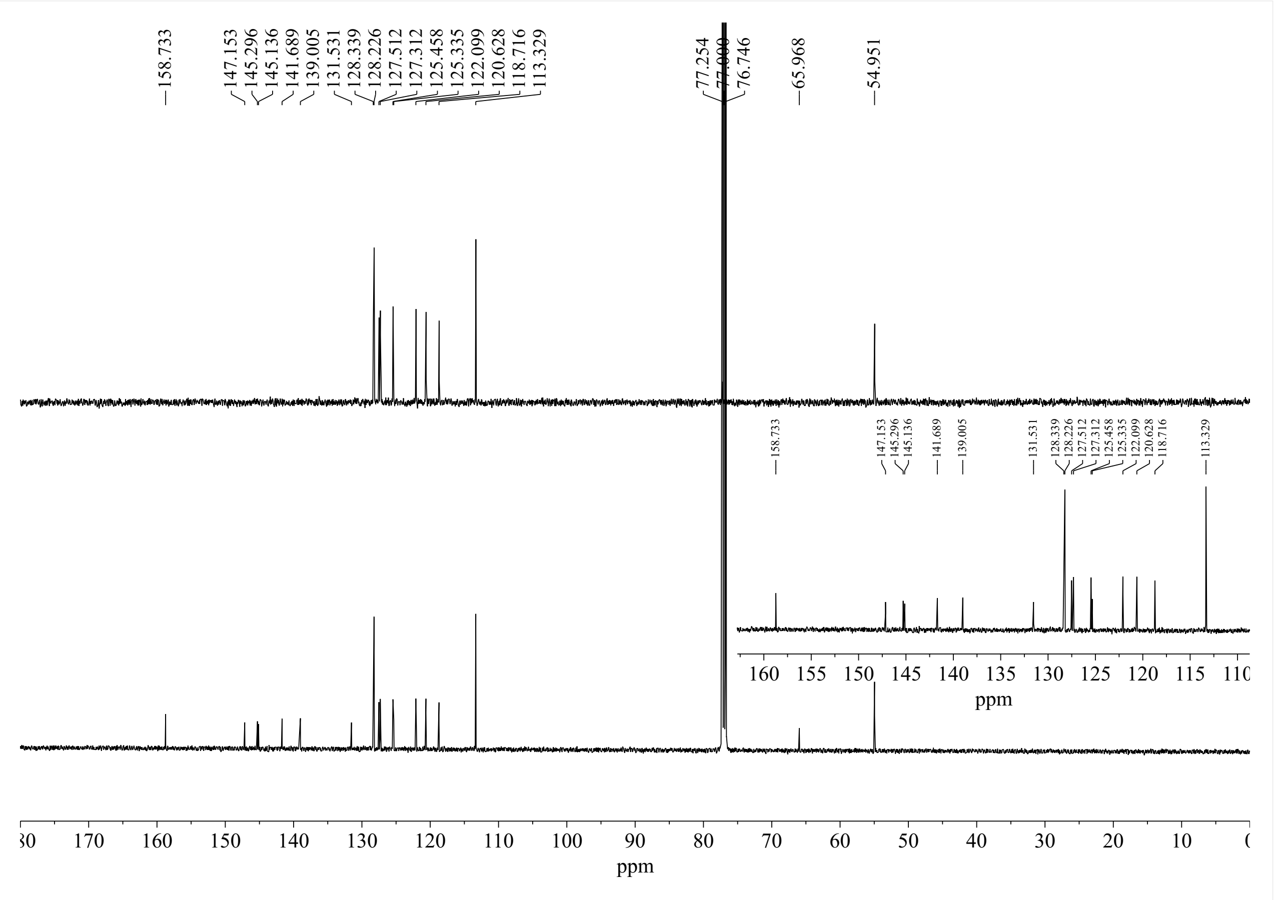

^{1}H (500 MHz) and ^{13}C (125 MHz) NMR spectra of **2d** in $CDCl_3$

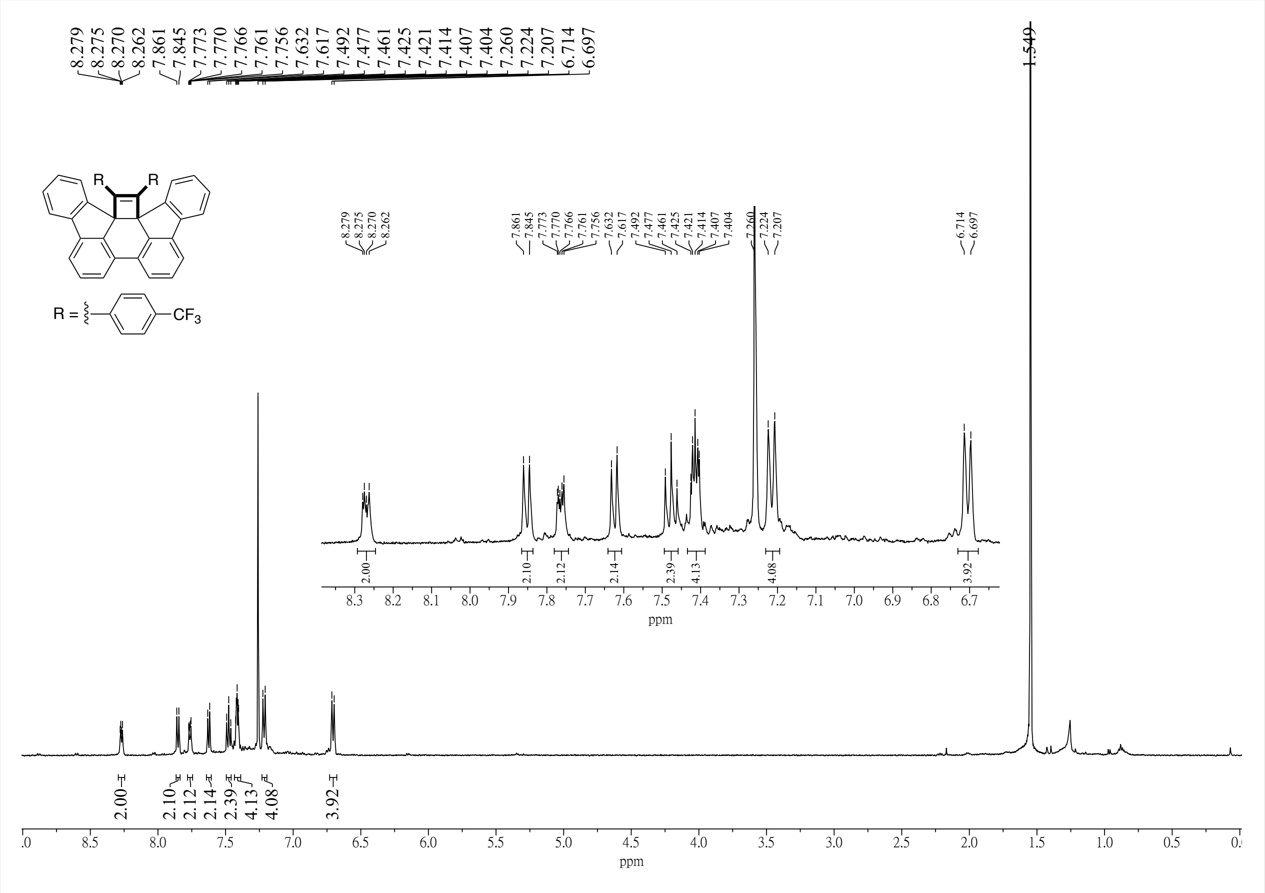

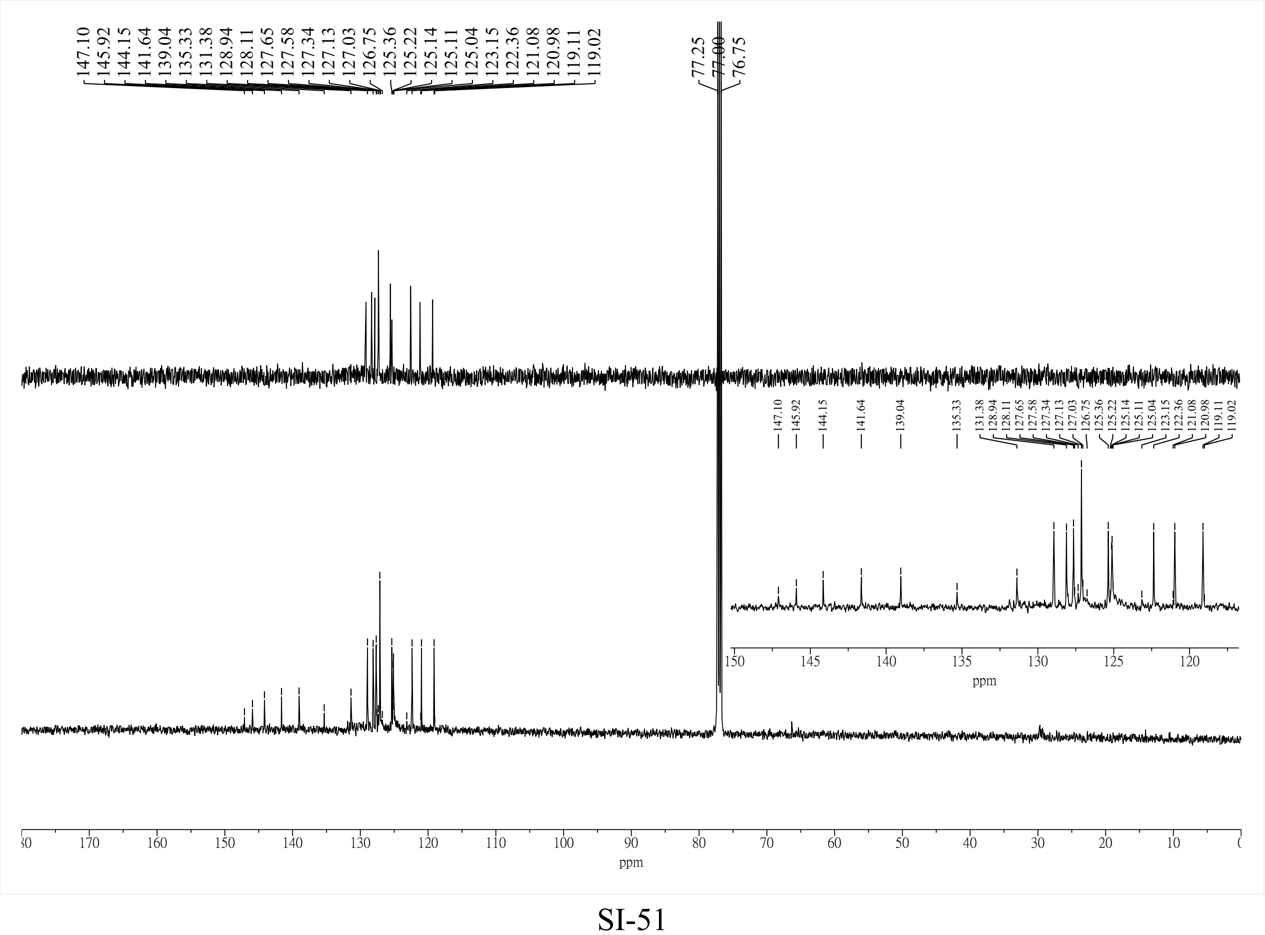

^{1}H (500 MHz) and ^{13}C (125 MHz) NMR spectra of **2g** in $CDCl_3$

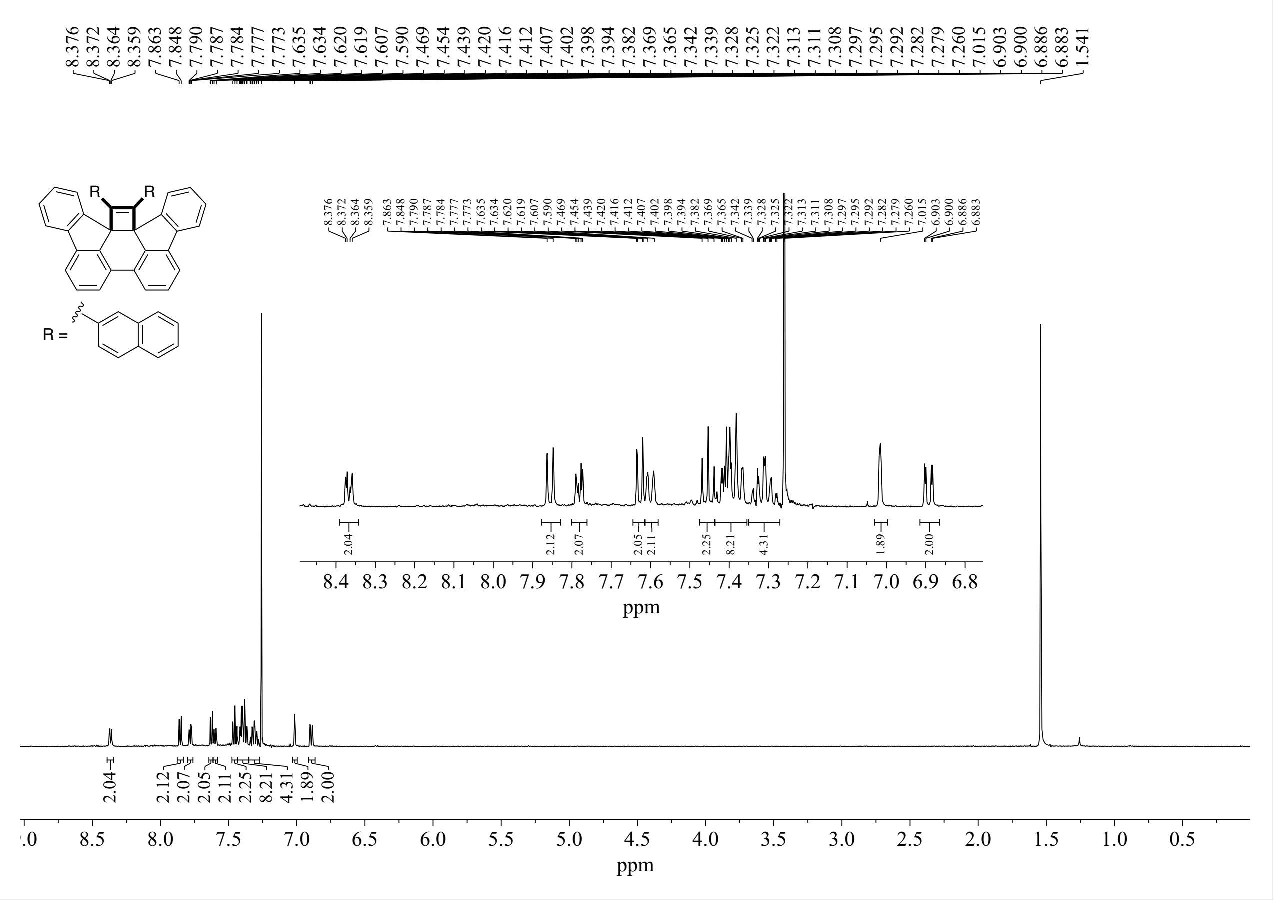

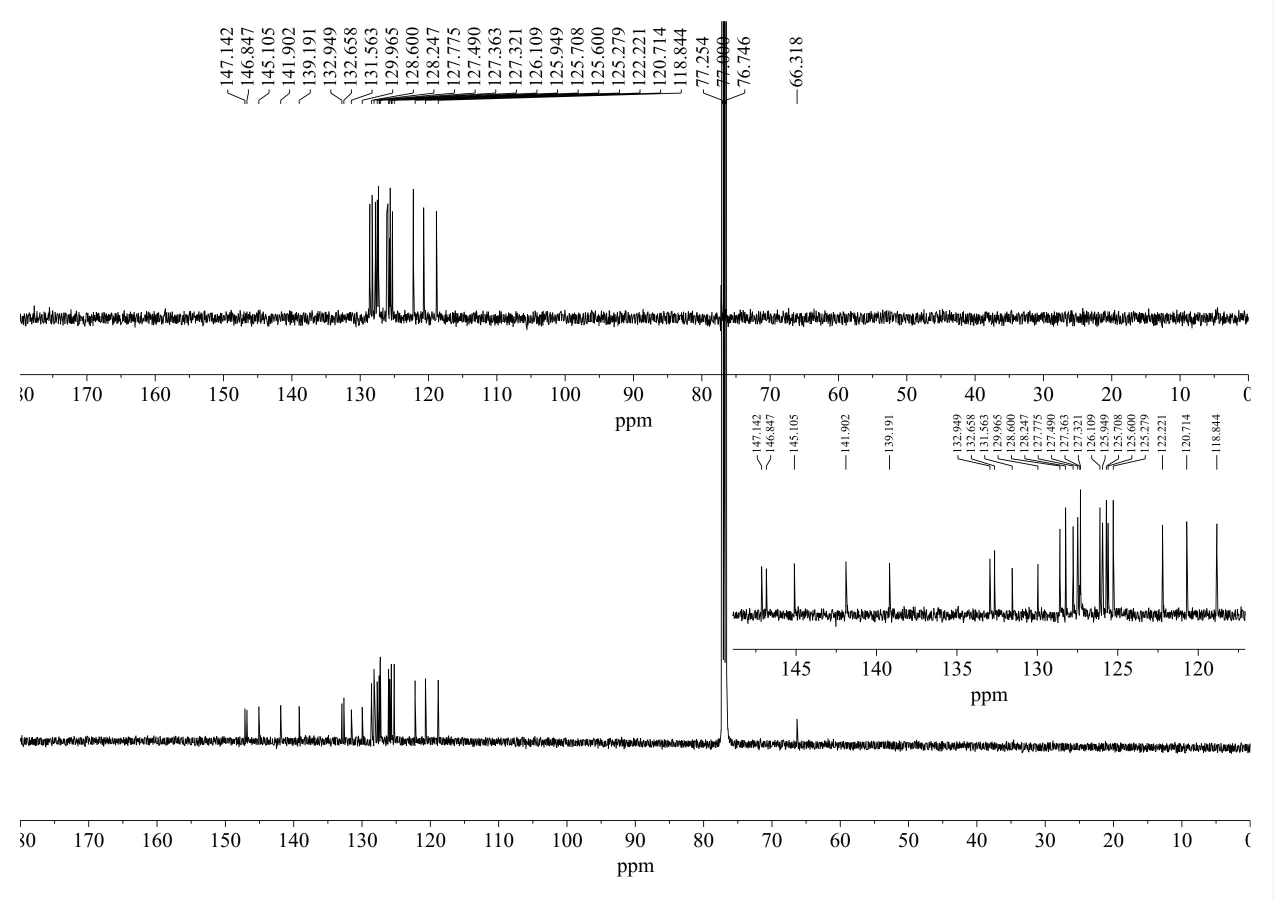

^{1}H (500 MHz) and ^{13}C (125 MHz) NMR spectra of **2h** in $CDCl_3$

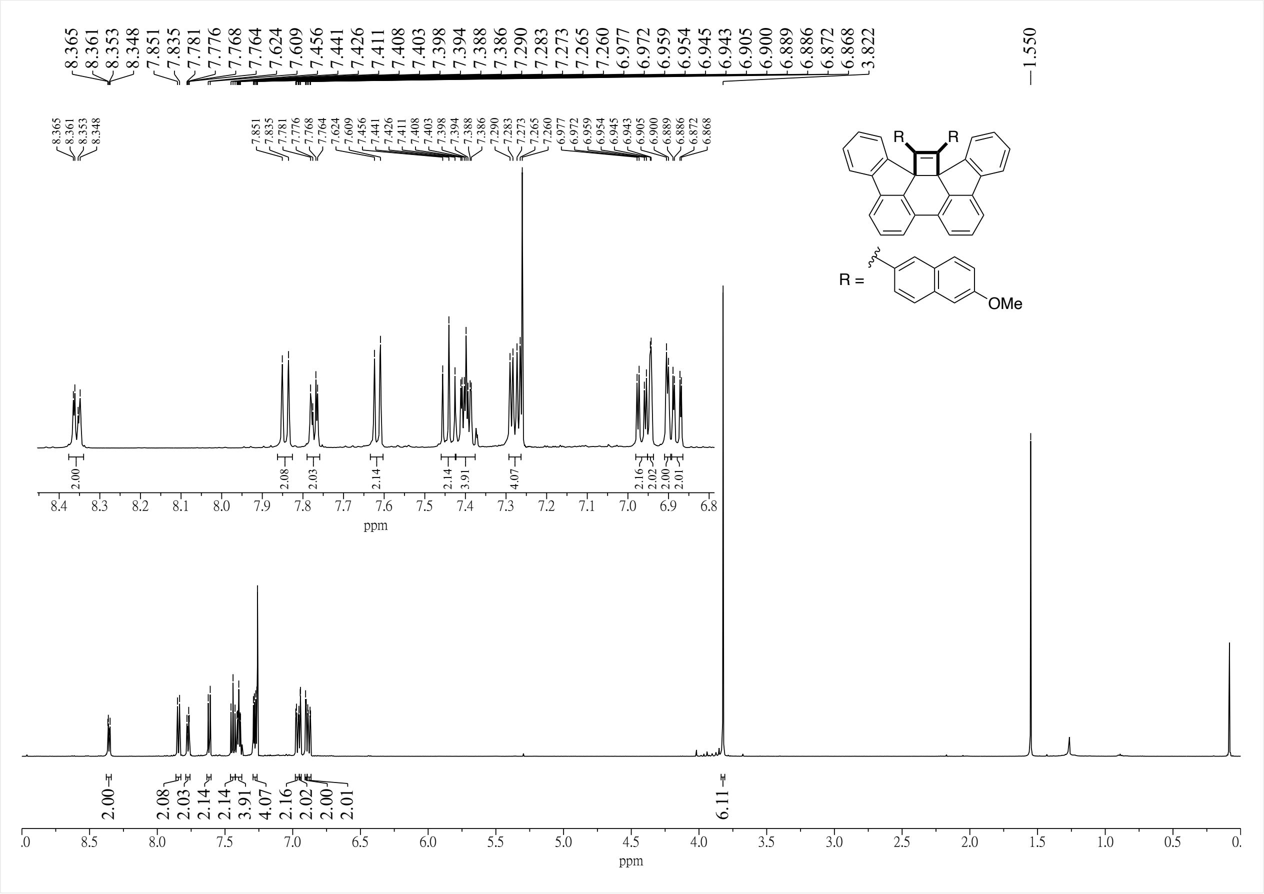

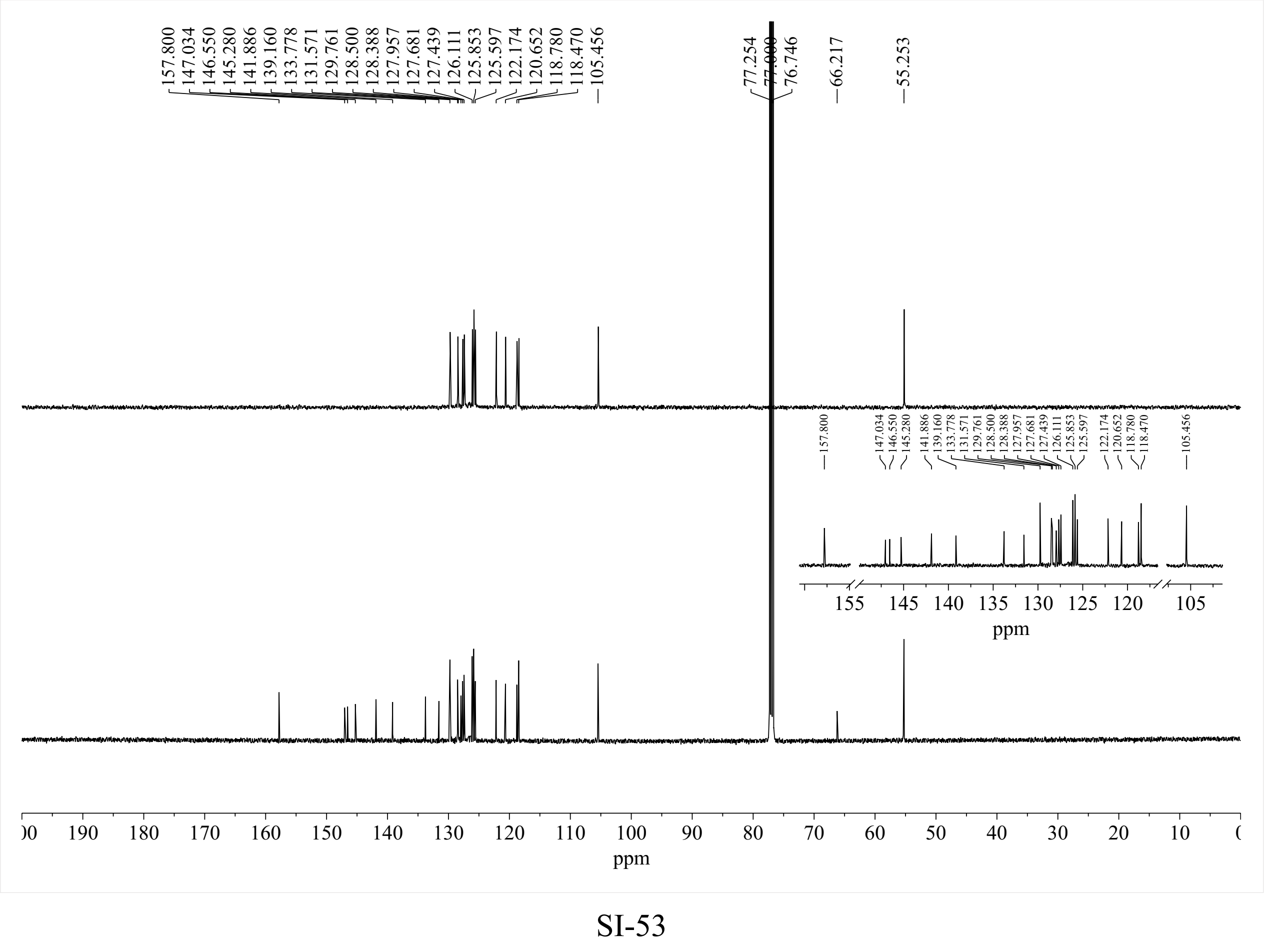

^{1}H (500 MHz) and ^{13}C (125 MHz) NMR spectra of **2i** in $CDCl_3$

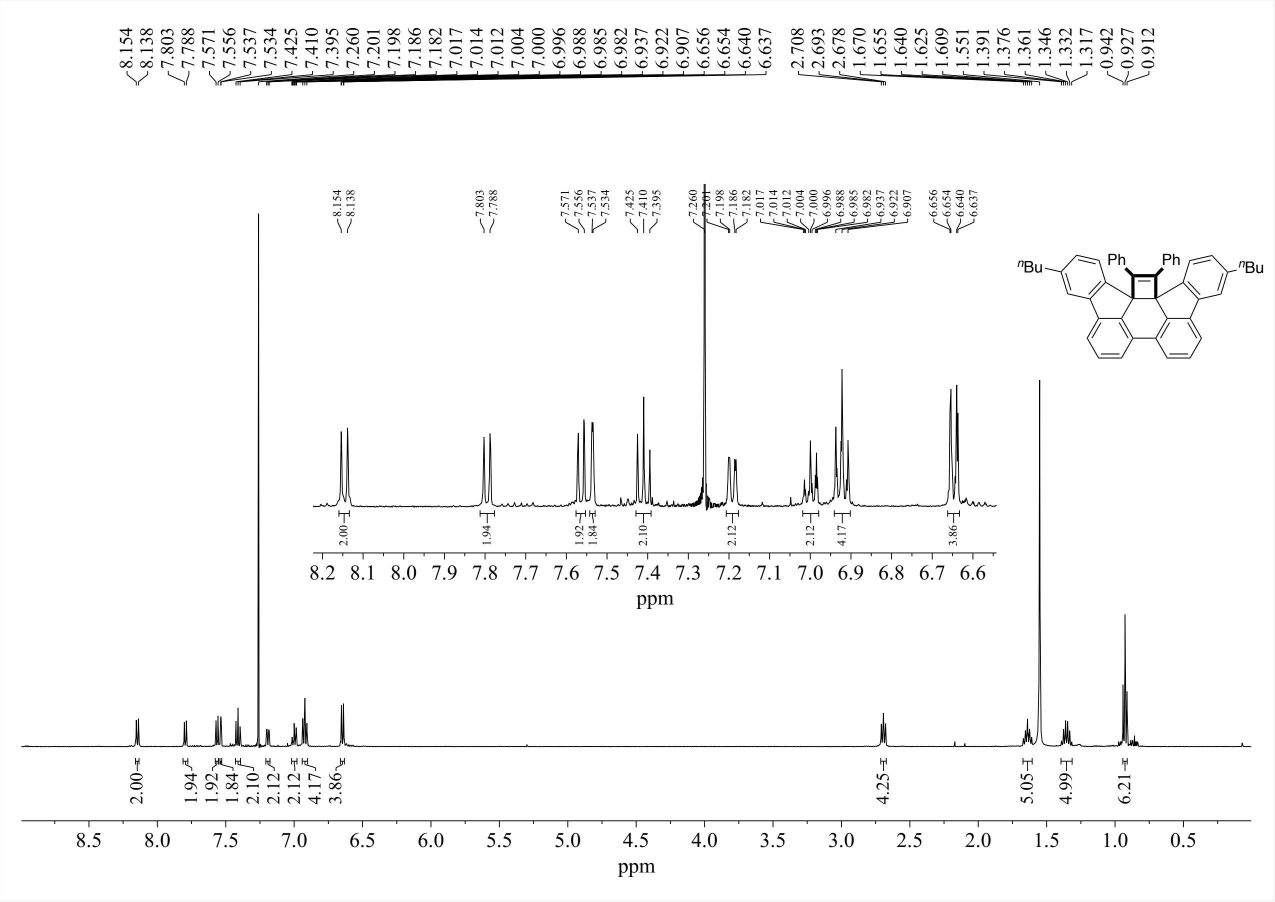

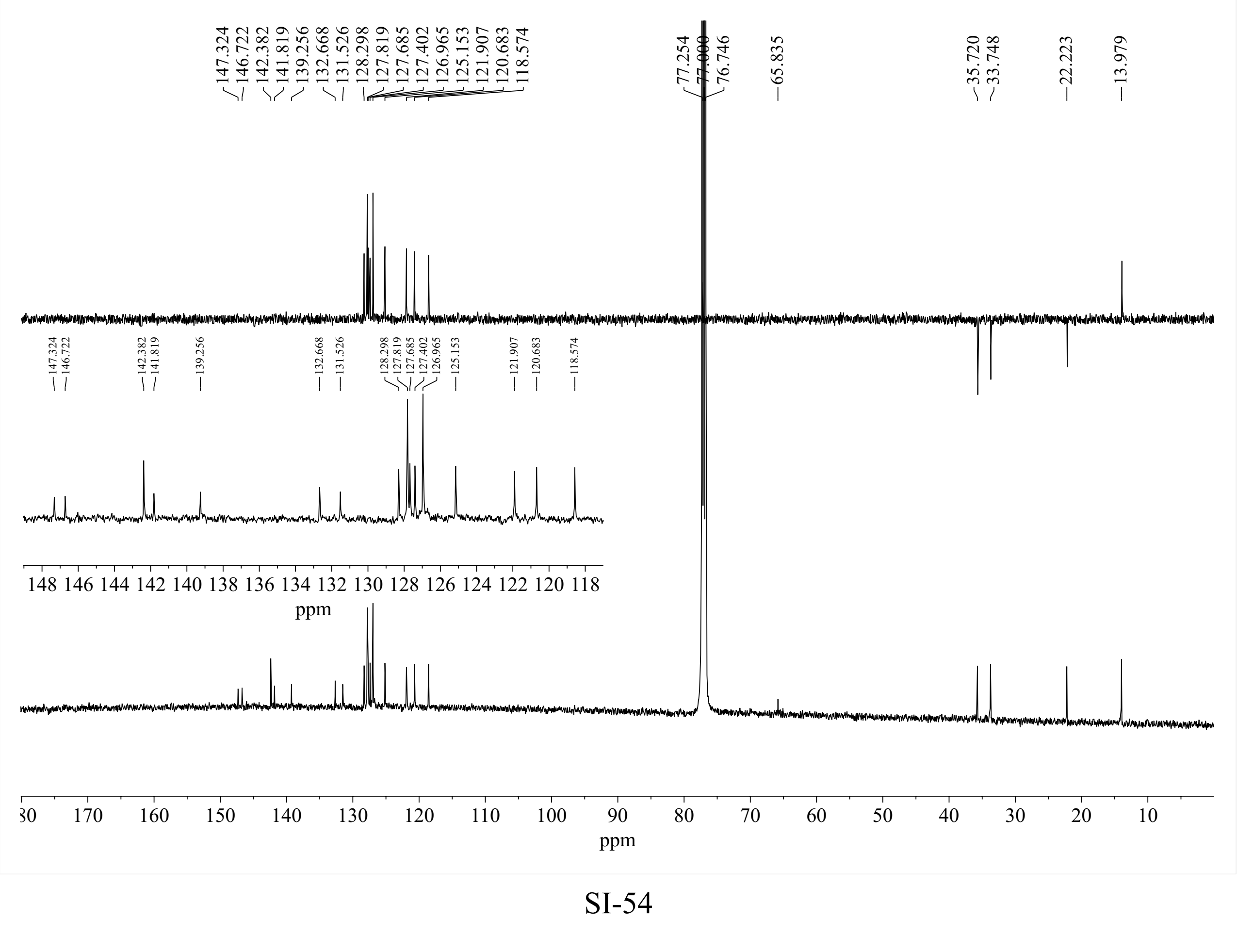

^{1}H (500 MHz) and ^{13}C (125 MHz) NMR spectra of **2j** in $CDCl_3$

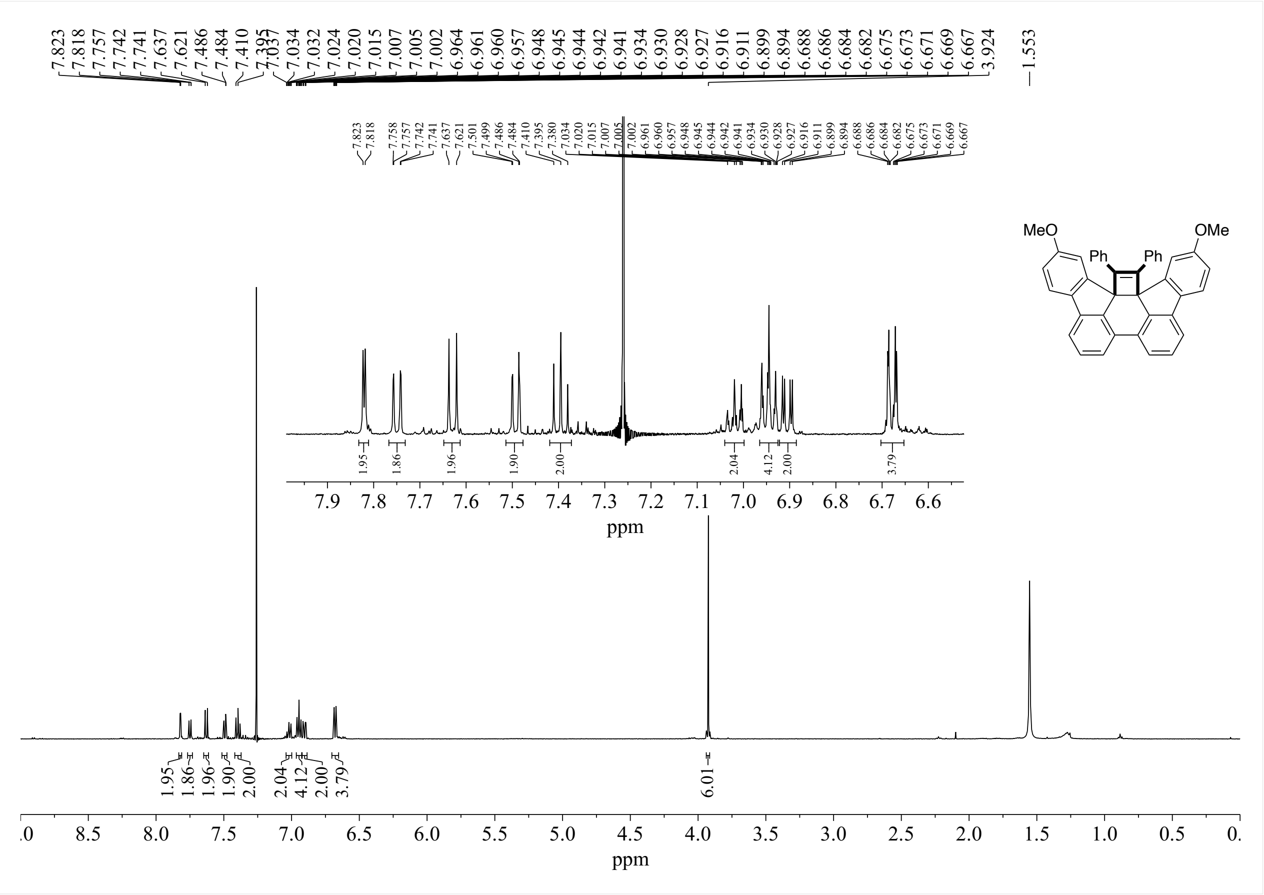

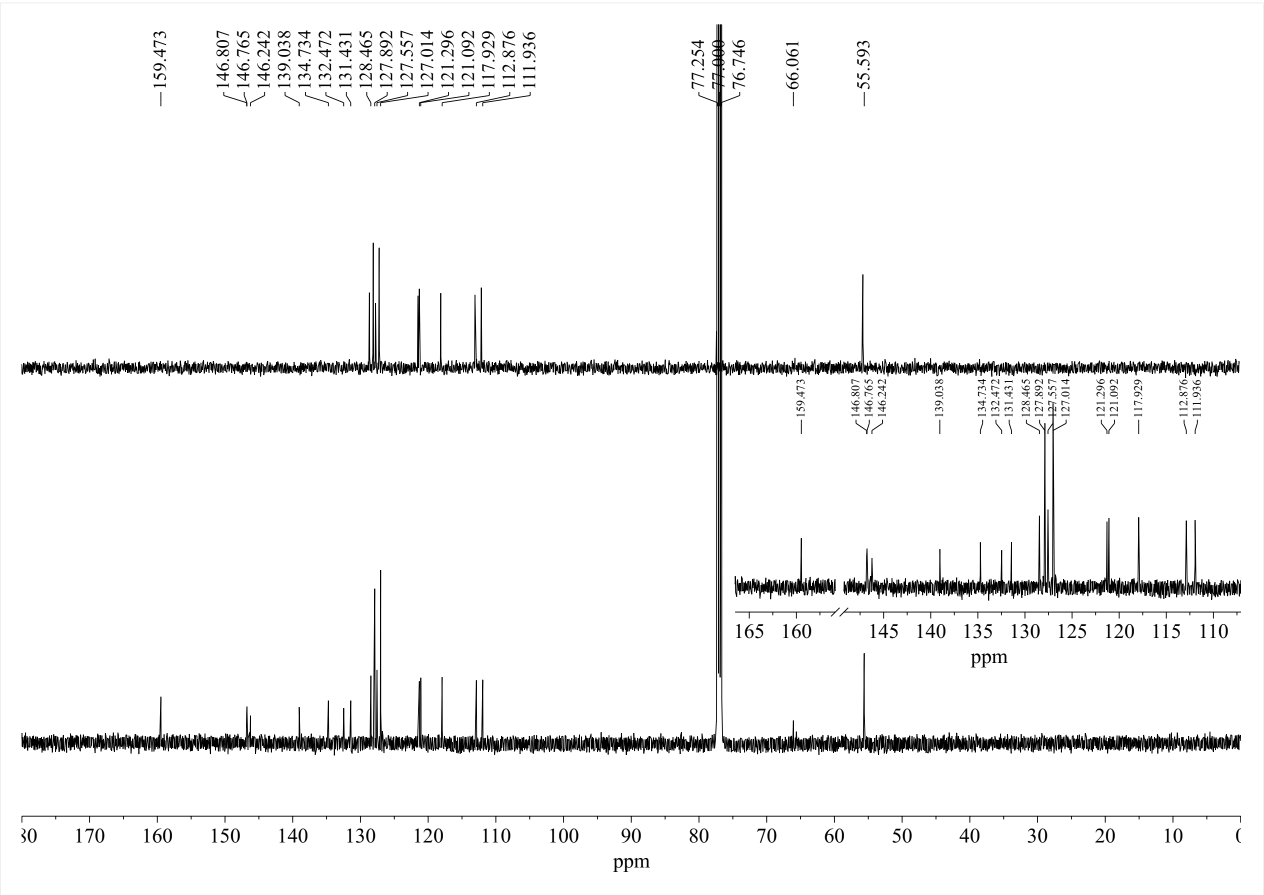

^{1}H (700 MHz) and ^{13}C (175 MHz) NMR spectrum of **3a** in $CDCl_3$

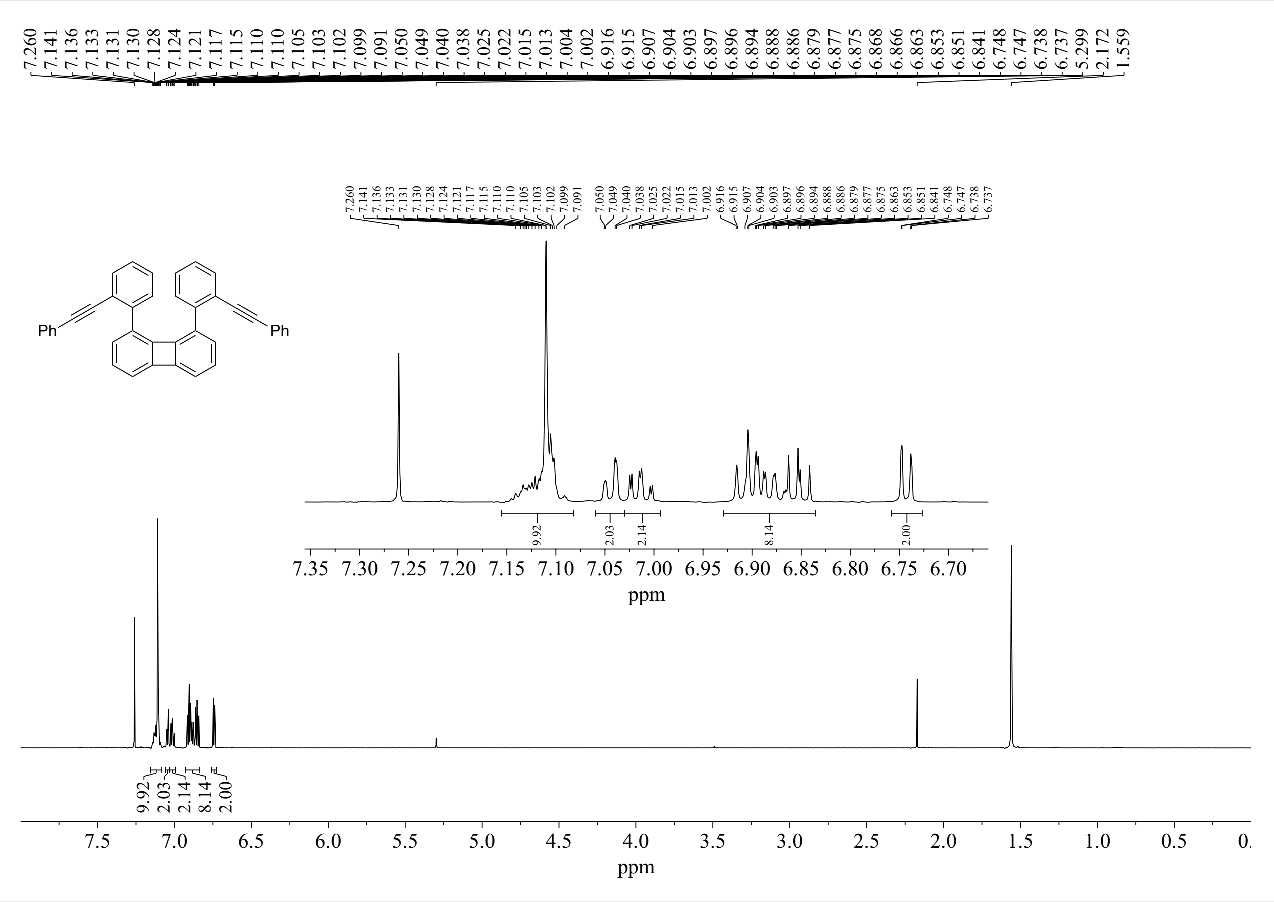

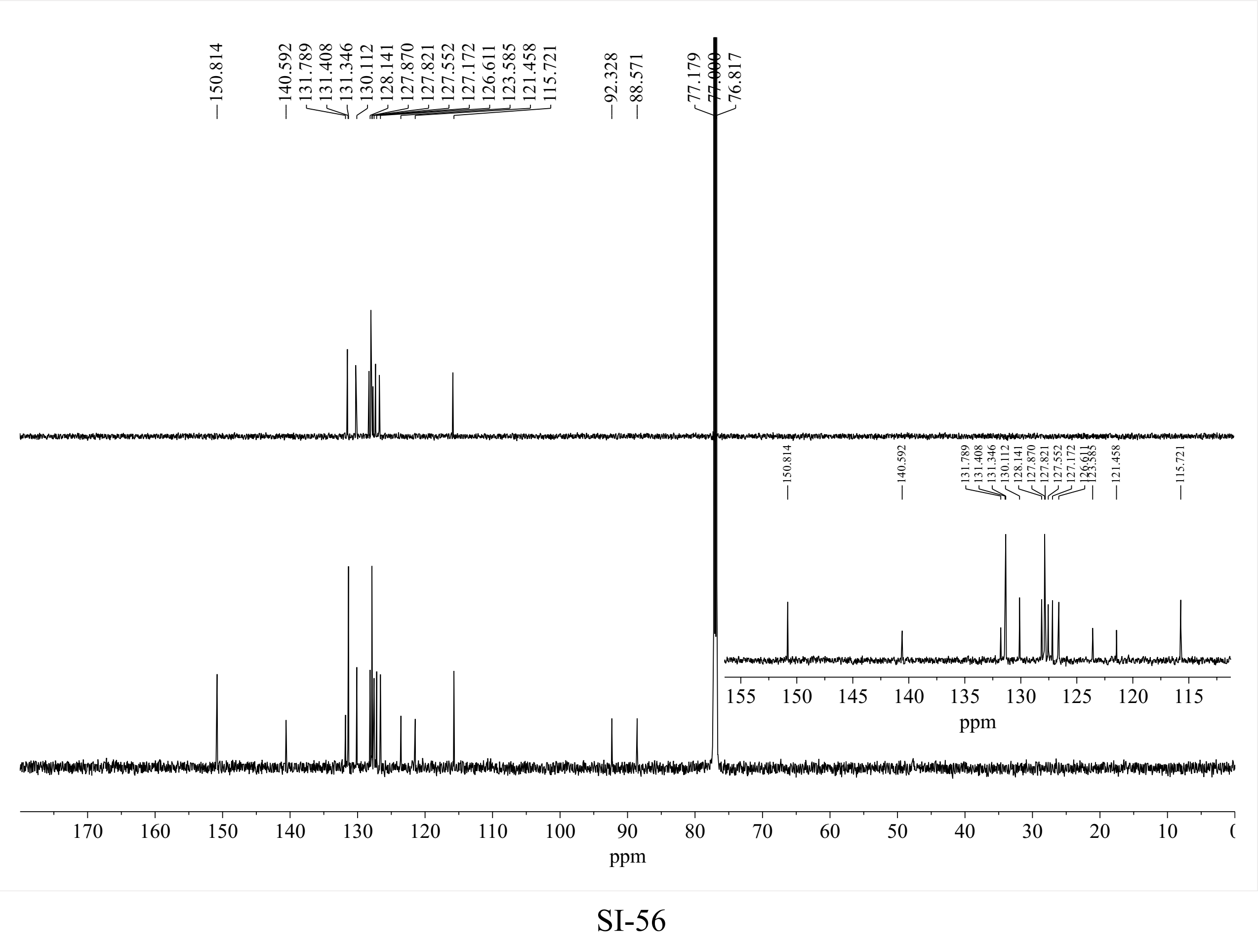

^{1}H (400 MHz) and ^{13}C (125 MHz) NMR spectrum of **3k** in $CDCl_3$

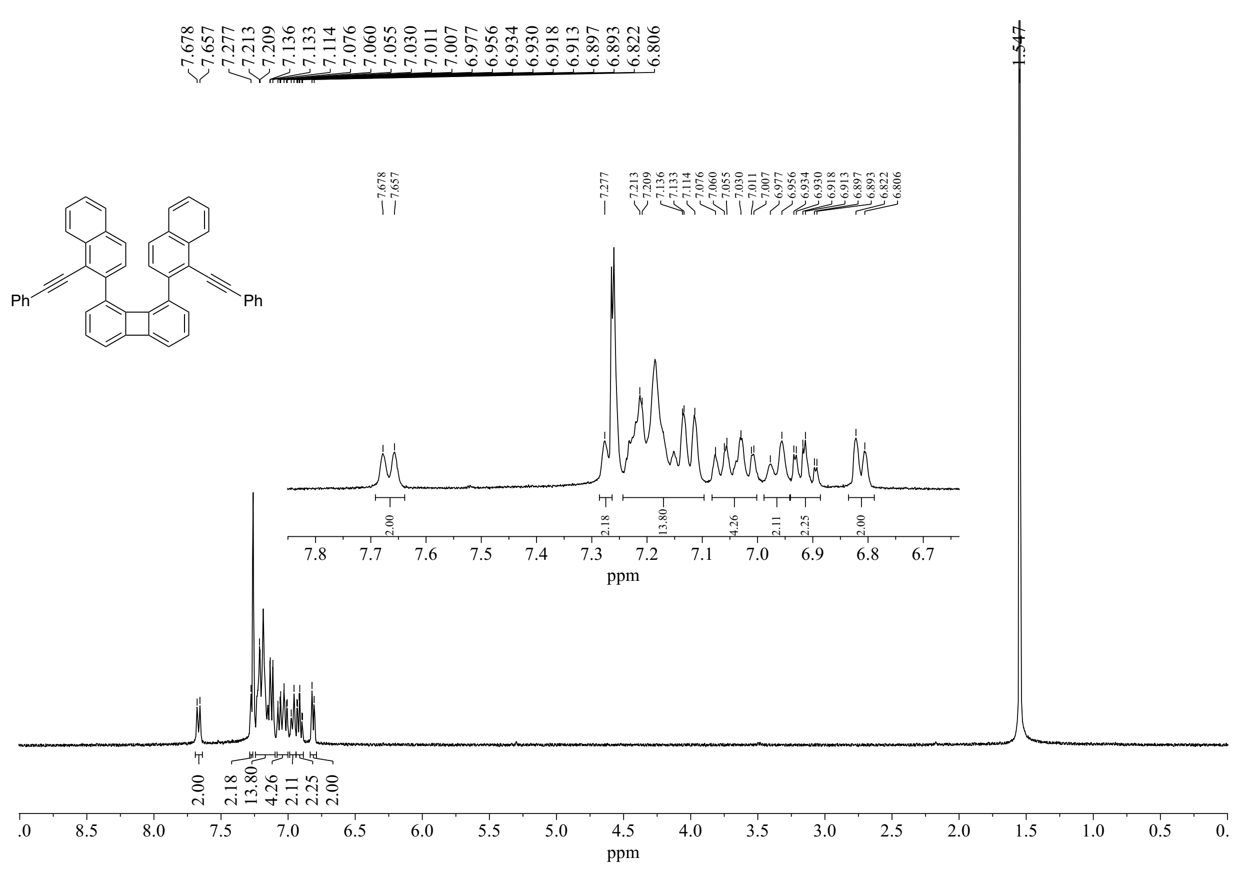

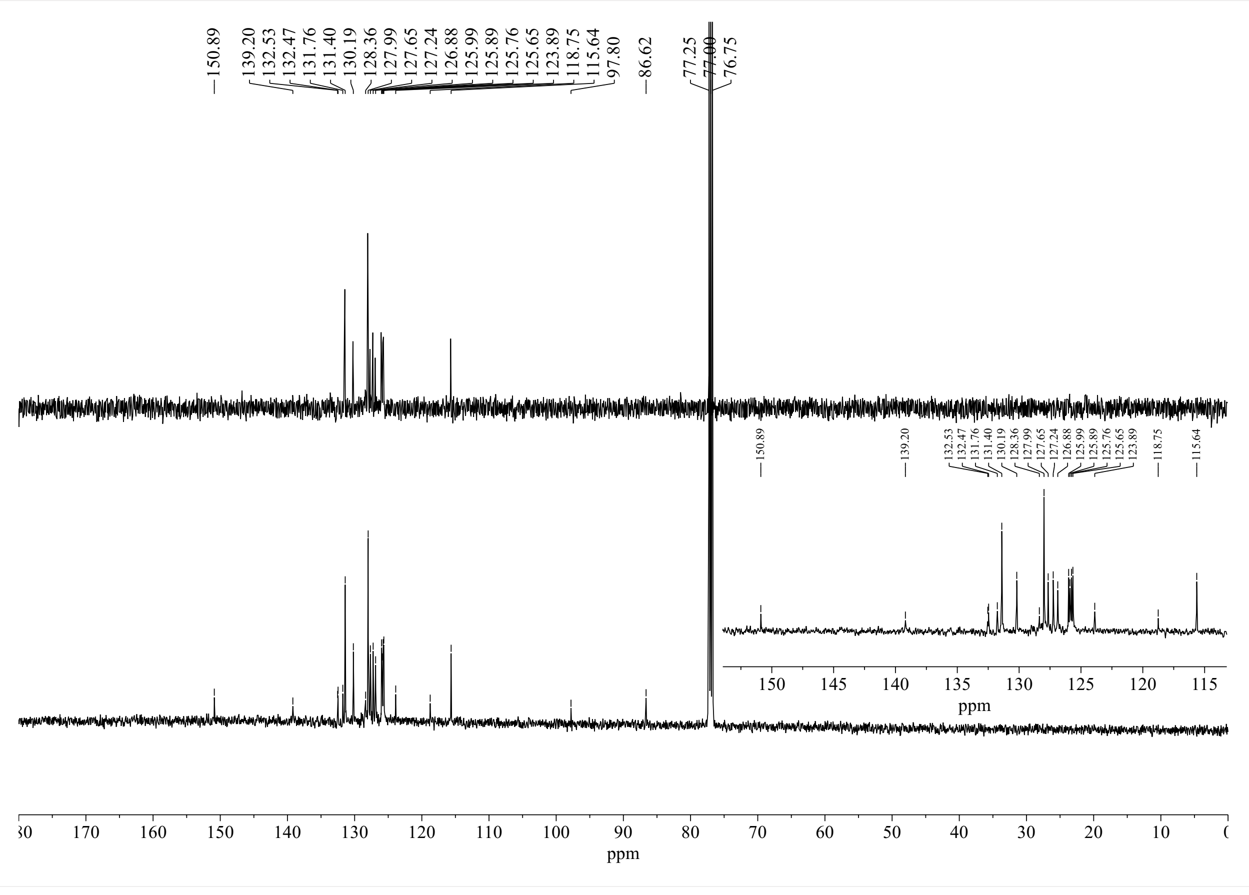

^{1}H (500 MHz) and ^{13}C (125 MHz) NMR spectra of **4a** in $CDCl_3$

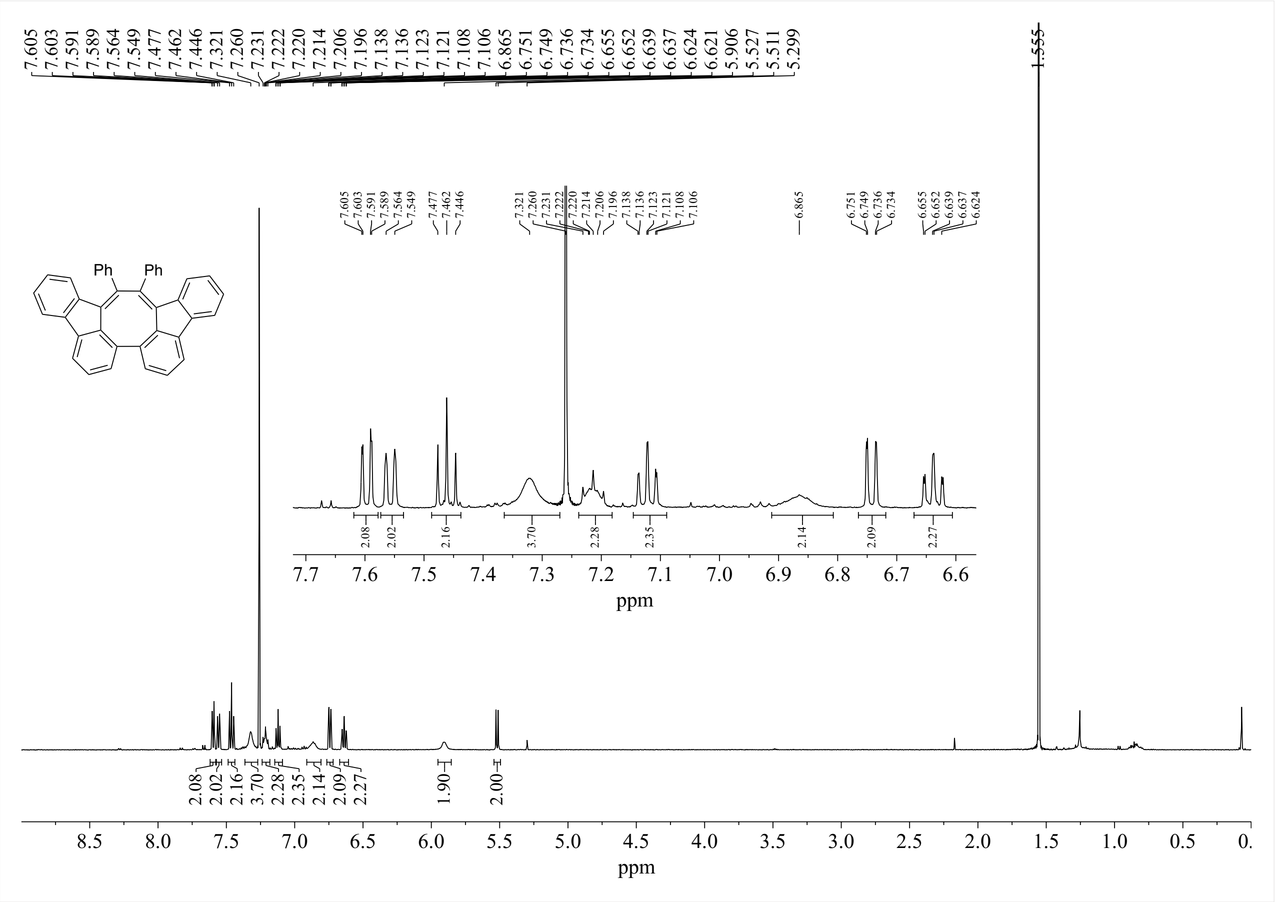

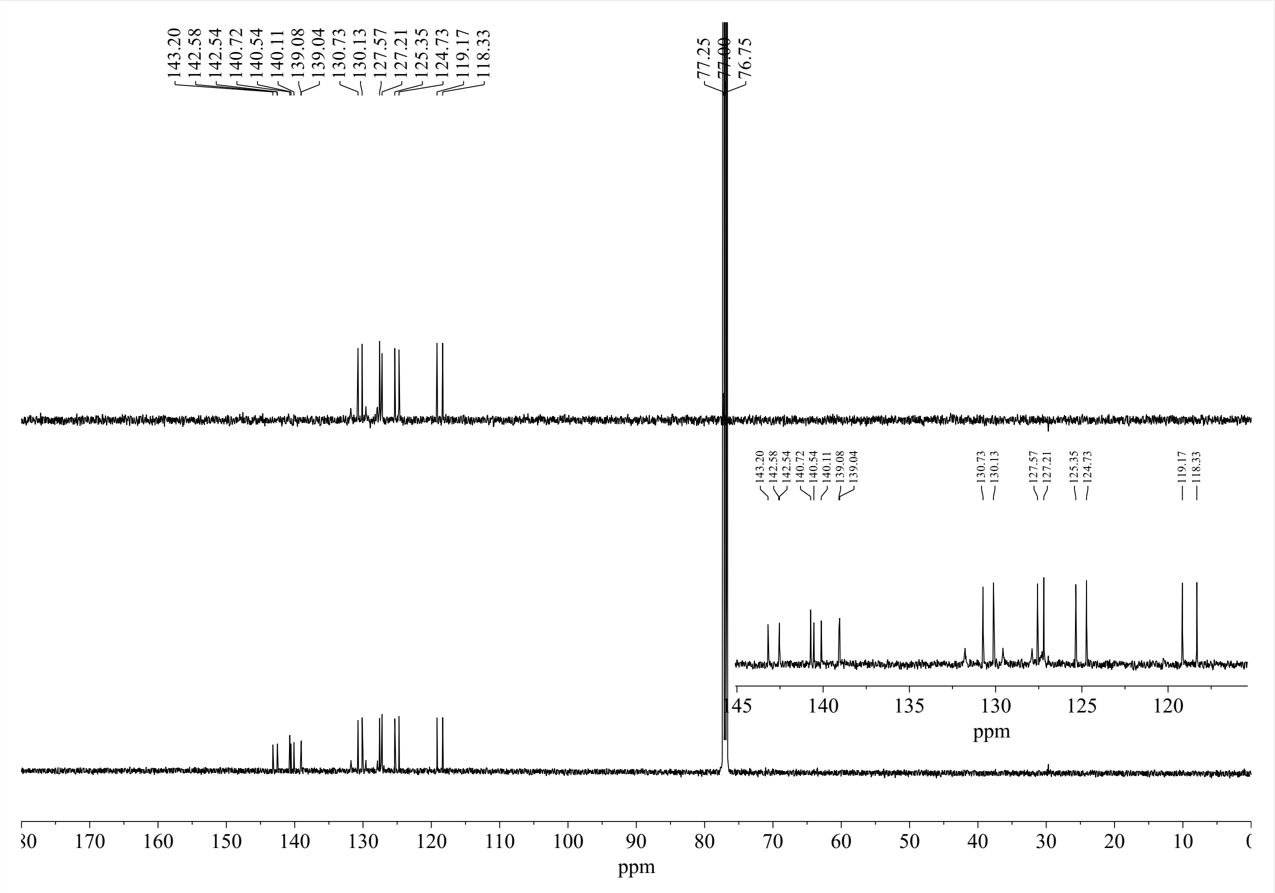

^{1}H (400 MHz) and ^{13}C (125 MHz) NMR spectra of **5** in $CDCl_3$

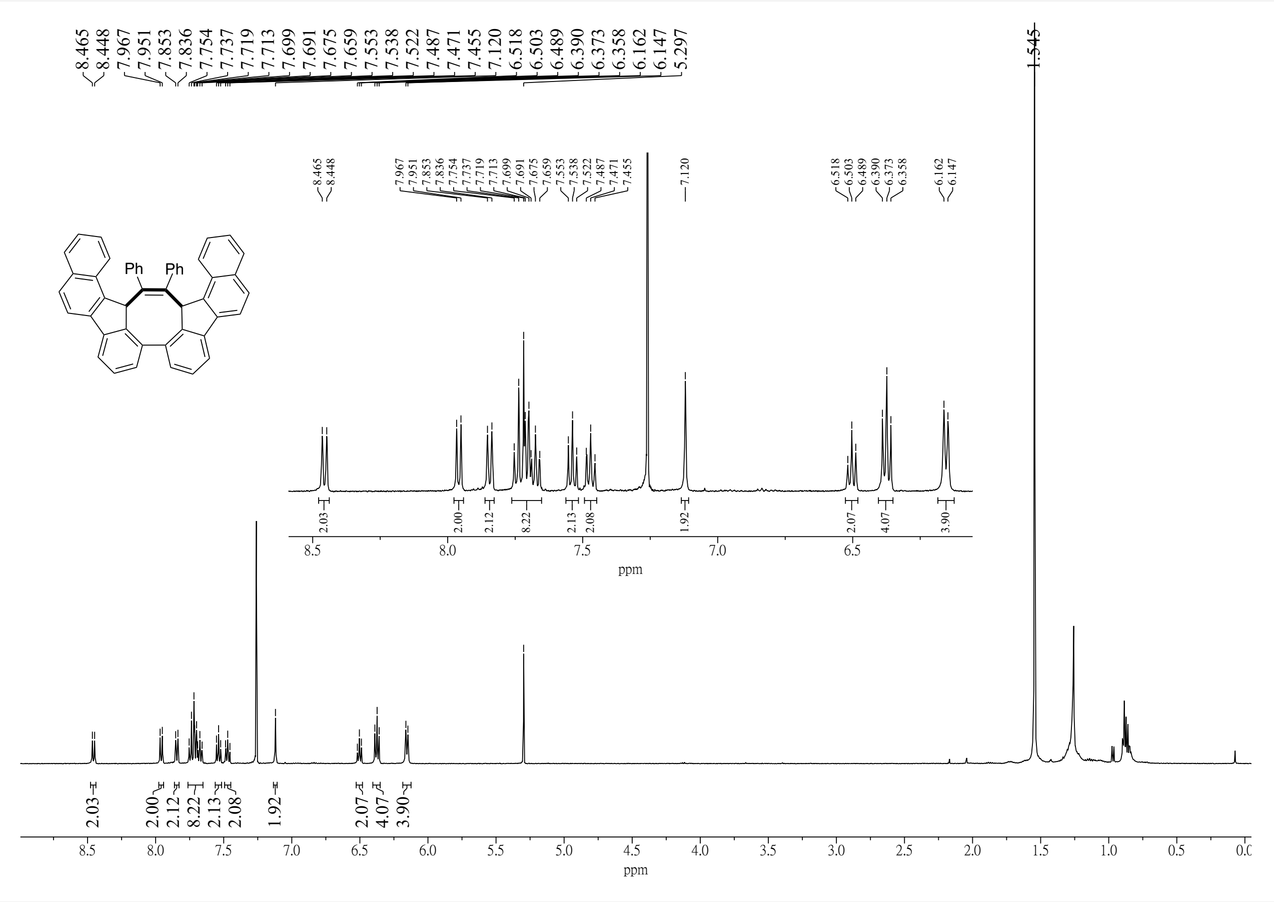

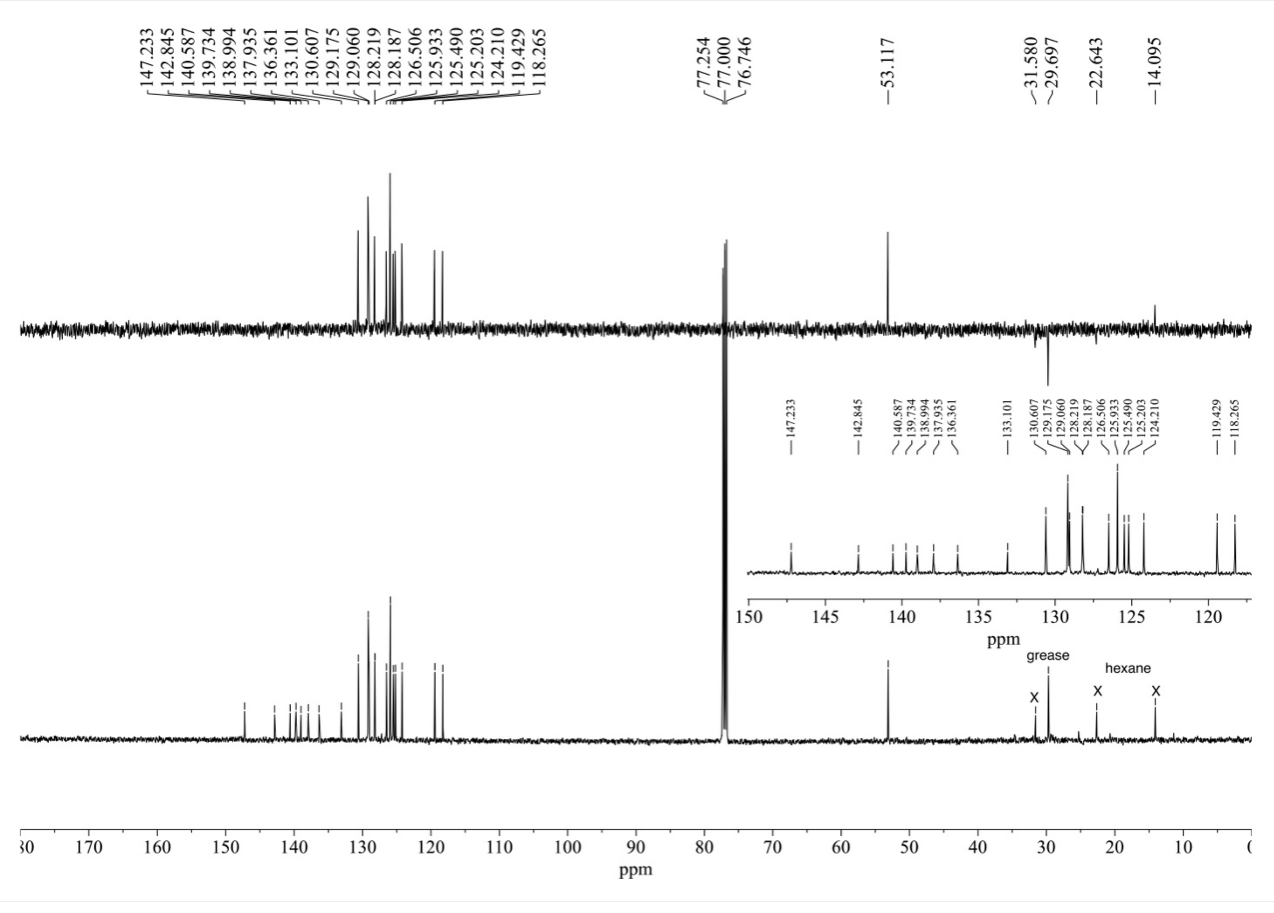

^{1}H (500 MHz) and ^{13}C (125 MHz) NMR spectra of **6** in $CDCl_3$

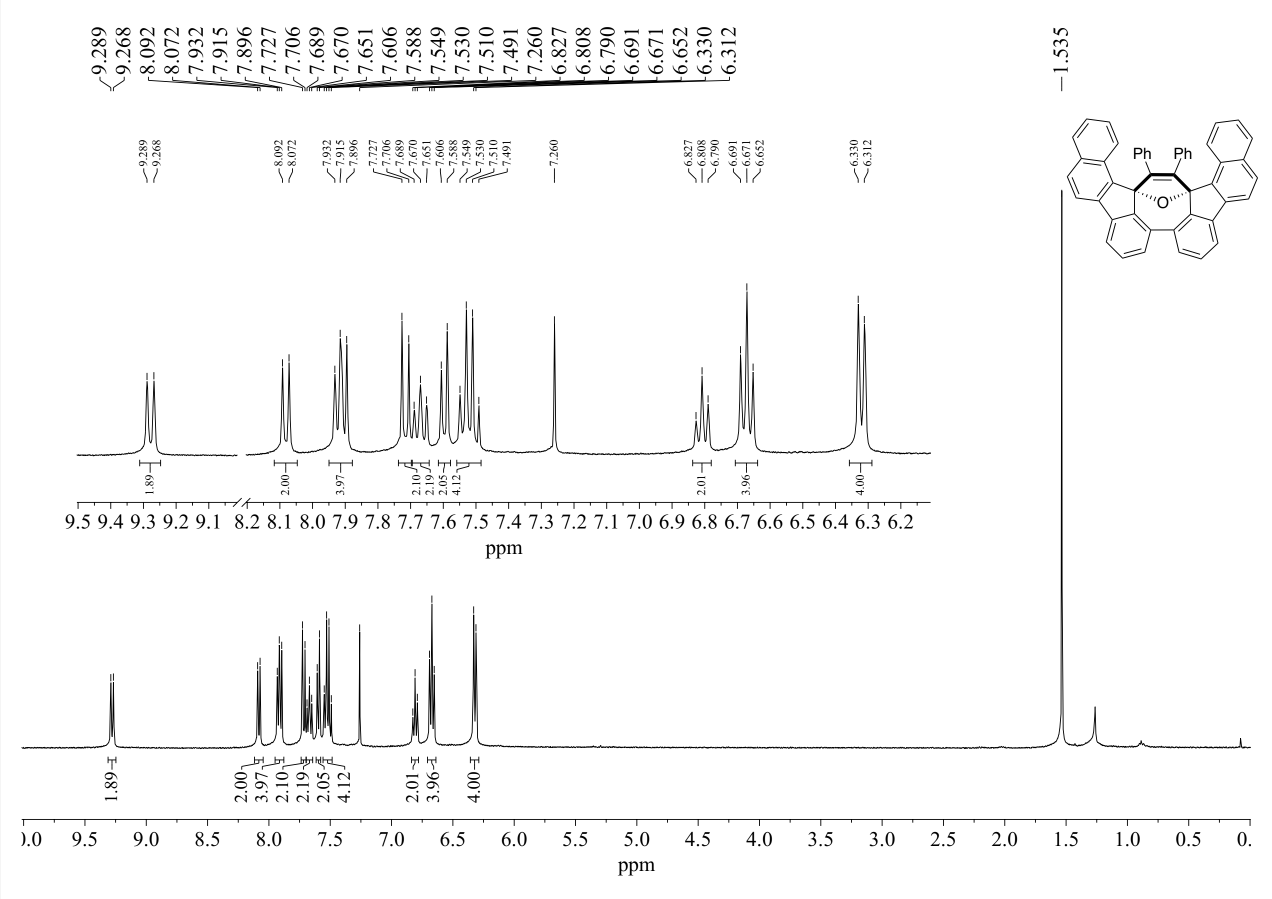

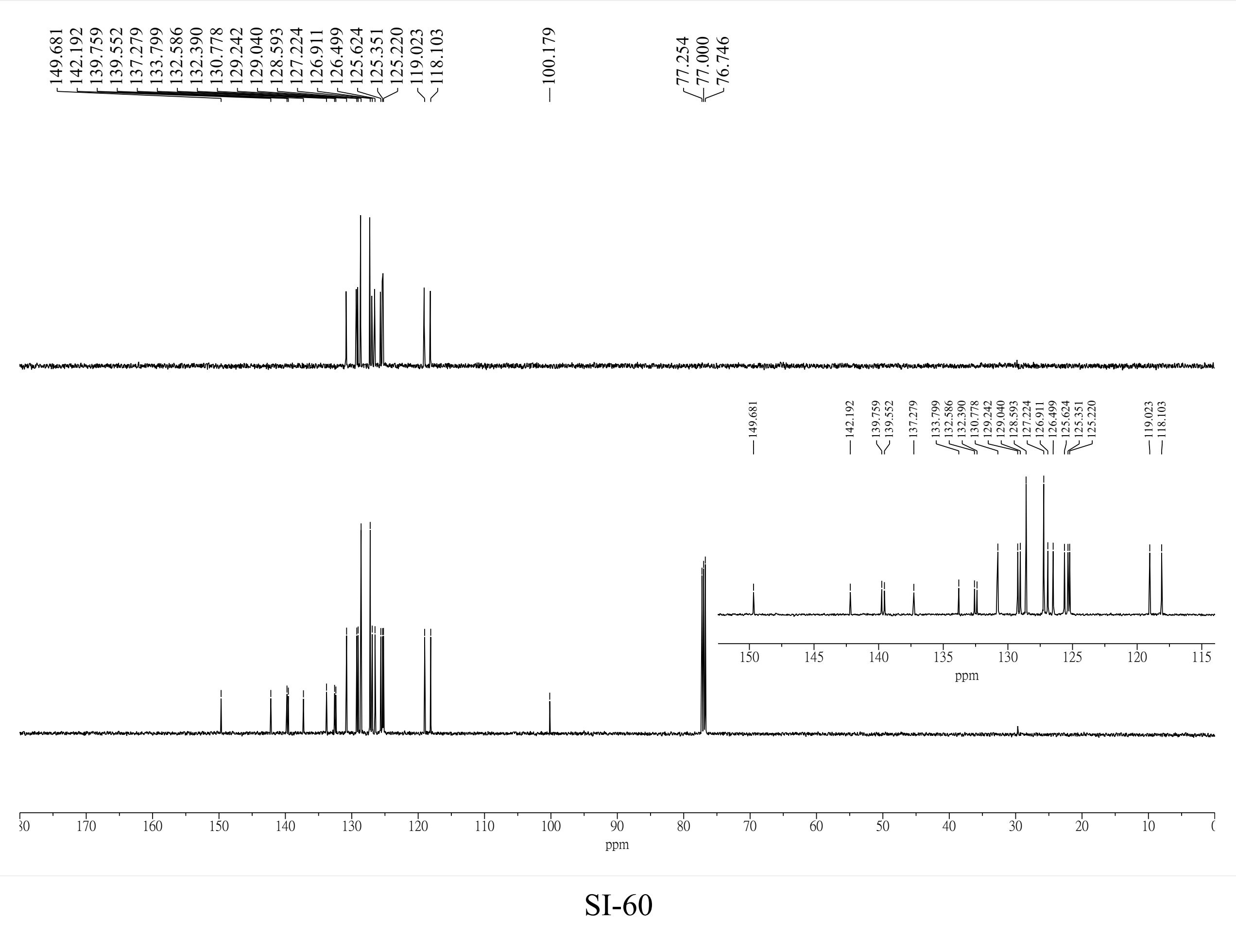

^{1}H NMR spectrum of compound **10** in $CDCl_3$ (400 MHz)

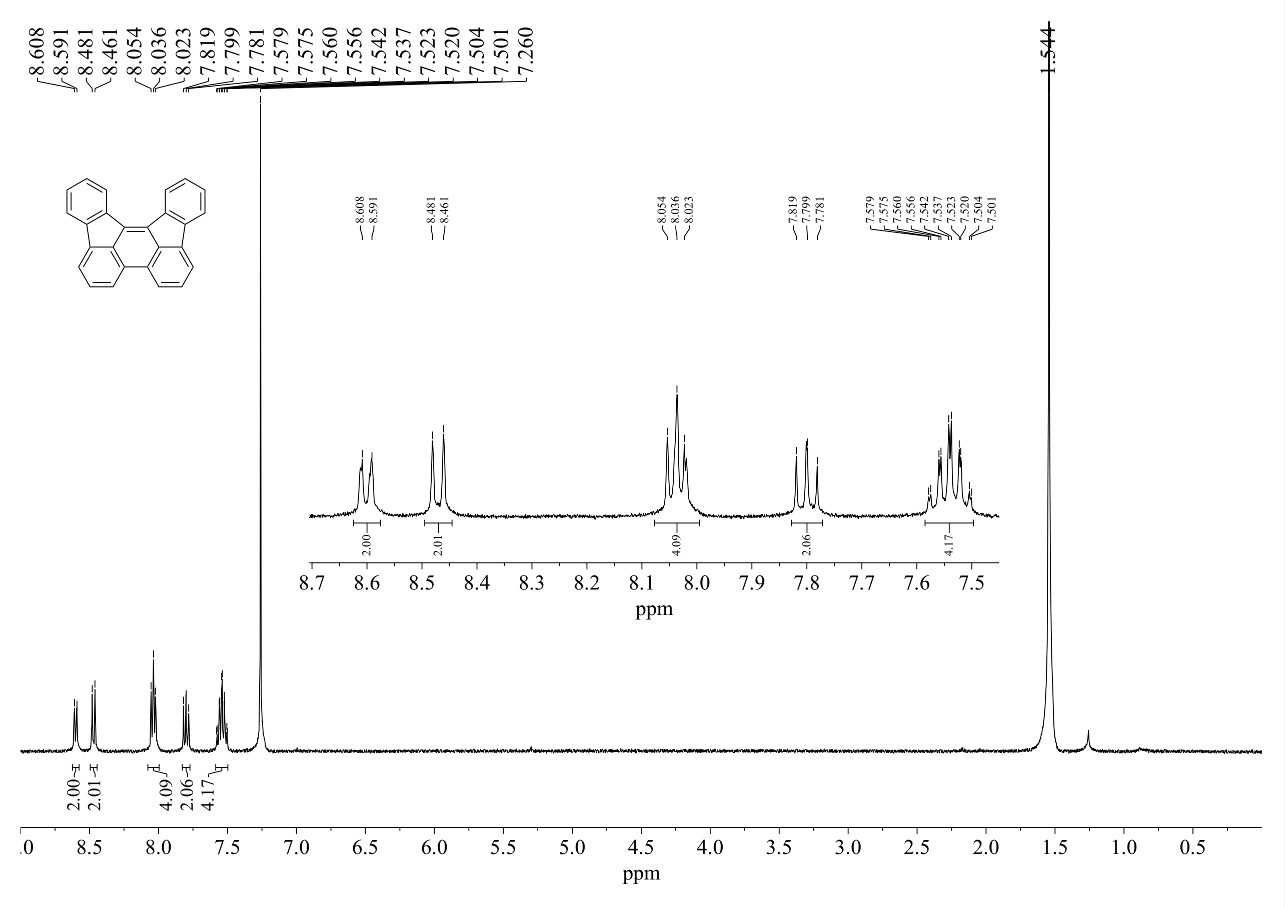

Supplement: Supplementary file 1 [file ol5c04302_si_001.pdf]
